# Unraveling the Effects of Fe Incorporation on High-Performance Water-Splitting Photoanodes

**DOI:** 10.1021/jacs.5c19704

**Published:** 2026-01-27

**Authors:** Kanokwan Klahan, Gilles Patriarche, Stephan Steinmann, Laureline Treps, Gilles Pécastaings, Osmane Camara, Rüdiger-A. Eichel, Sylvain Chambon, Patrick Garrigue, Cécile Bossy, Sareeya Bureekaew, Gabriel Loget, Pichaya Pattanasattayavong

**Affiliations:** † Department of Materials Science and Engineering, School of Molecular Science and Engineering, Vidyasirimedhi Institute of Science and Technology (VISTEC), Rayong 21210, Thailand; ‡ University of Bordeaux, Bordeaux INP, ISM, UMR CNRS 5255, 33607 Pessac, France; § Centre de Nanosciences et de Nanotechnologies, 27048 Université Paris-Saclay, CNRS, Bld Thomas Gobert, 91120 Palaiseau, France; ∥ ENS de Lyon, CNRS, Laboratoire de Chimie, 69342 Lyon, France; ⊥ University of Bordeaux, CNRS, CRPP, UMR 5031, F-33600 Pessac, France; # Institute of Energy Technologies, Fundamental Electrochemistry (IET-1), Forschungszentrum, Jülich 52425, Germany; ∇ University of Bordeaux, CNRS, Bordeaux INP, IMS, UMR 5218, F-33400 Talence, France; ○ University of Bordeaux, CNRS, Bordeaux INP, EPOC, UMR 5805, F-33600 Pessac, France; ◆ School of Energy Science and Engineering, Vidyasirimedhi Institute of Science and Technology (VISTEC), Rayong 21210, Thailand

## Abstract

Although it is known that iron (Fe) significantly alters the electrocatalytic activity of nickel (Ni)-based materials, little attention has been paid to the effects of Fe impurities on the photoelectrochemical (PEC) properties of solar-driven water-splitting photoanodes. Herein, we elucidate the crucial role of Fe in model metal–insulator–semiconductor (MIS) Si photoanodes decorated with Ni nanoparticles (NPs), known for their high performance in photoinduced water splitting. Our results demonstrate that residual Fe strongly influences the photoanodes’ junction energetics and photovoltaic properties. We show that the synergistic effects (electrocatalytic/photovoltaic) caused by Fe doping explain the high performance previously reported for these model photoanodes. Crucially, Fe incorporation into the outer shell of Ni NPs and the electrolyte is essential to achieve the reported photovoltage up to 500 mV. Our investigations emphasize the importance of Fe in PEC devices, which has always been neglected in the past.

## Graphical abstract

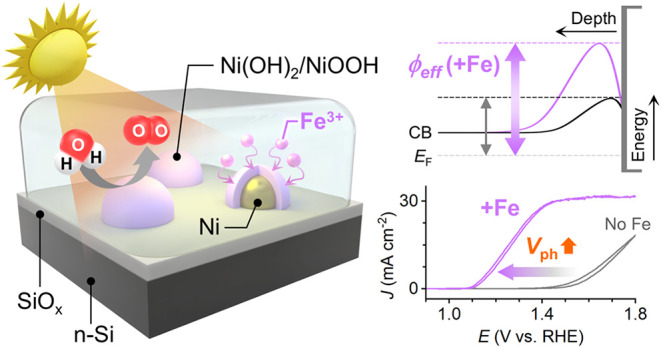

## Introduction

To meet the growing demand for clean and renewable energy sources, the development of sustainable hydrogen (H_2_) fuel production is being intensively developed.^1,2^ Solar water splitting is one of the promising technologies (e.g., via photocatalysis, photoelectrocatalysis, or coupled photovoltaic-electrolysis) to produce green H_2_ along with O_2_ by utilizing sunlight energy.^3,4^ The development of photoelectrodes and, particularly, photoanodes for driving water oxidation or oxygen evolution reaction (OER) is crucial to achieve stable and high-performance photoelectrochemical (PEC) devices.^5–7^ n-Type silicon (n-Si) has become an attractive choice as photoanodes due to its abundance, availability of large-scale production infrastructure, and appropriate band gap for optimal solar light absorption.^8,9^ Unfortunately, Si suffers from degradation/corrosion during PEC operation.^10^ Numerous efforts have been devoted to improving n-Si photoanodes.^11–13^ To this end, Si-based photoanodes with an inhomogeneous metal–insulator-semiconductor (MIS) junction have been thoroughly studied over the past decade as a promising candidate due to their robust, high PEC performance. These Si photoanodes are typically modified with transition metal nanoparticles (NPs) having a large work function and high electrocatalytic activity, e.g., Ni,^14^ Co,^15^ or Fe.^16^ Despite their simple and low-cost fabrication, these photoanodes often exhibit an exceptional PEC performance for solar water oxidation, i.e., large photovoltage (above 400 mV), low onset potential (*E*_on_), high current density, and long operational stability,^8,14^ which makes them highly attractive for the manufacturing of sun-to-H_2_ conversion systems. The enhanced photovoltage of these inhomogeneous MIS Si photoanodes, when compared to their planar MIS junction counterparts, has been initially ascribed to the pinch-off effect, where the high-energy barrier area surrounding metal NPs facilitates hole (h^+^) transport to the surface for water oxidation.^10,17–19^ This effect occurs when NP sizes are small enough, and most studies employed Ni NPs as the standard model for this system. Boettcher et al. reported that the formation of the high work function NiOOH shell covering Ni NPs was crucial in achieving a large effective barrier height and photovoltage.^18^ Recently, different approaches have been proposed to explain this particular phenomenon, e.g., the presence of the insulating interfacial SiO_*x*_ layer^20^ and the interfacial potential drop.^21^

For Ni-based catalysts, Fe impurities, which are unintentionally present in alkaline electrolytes, have been found to greatly improve the electrocatalytic activity toward water oxidation.^22,23^ This has led to intensive studies that have shed light on how the presence of adventitious Fe affects the electrochemical performance even at the sub-ppm level.^24,25^ In the field of electrocatalysis, this phenomenon has long been acknowledged, and NiFe-based catalysts [e.g., Fe-doped NiOOH or Ni­(Fe)­OOH] are widely recognized as one of the most active non-noble electrocatalysts for water oxidation in alkaline electrolyzers.^26–28^

Undoubtedly, Fe incorporation also occurs in the aforementioned Si photoanodes modified with Ni NPs since these photoelectrodes are mostly operated in alkaline electrolytes. This was early reported by Loget et al., who detected the presence of Fe in the hydroxide/oxyhydroxide [Ni­(OH)_2_/NiOOH] shell that was generated *in situ* around Ni NPs present on Si photoanodes,^14^ suggesting that the as-created Ni­(Fe)­OOH would confer different electrocatalytic properties compared to pure Ni NPs. Despite this, the effects of Fe impurities on this PEC system have yet to be explored from a holistic point of view. The incorporation of Fe into Ni­(OH)_2_/NiOOH was also overlooked in previous mechanistic studies as it was thought to only contribute to the electrocatalytic improvement.^18,20,21,29,30^ However, the electronic properties of the transition metal oxide-based semiconductors can be greatly altered through the doping of different metals and the changes in the local structure,^31,32^ which also likely occur in the case of Ni­(Fe)­OOH.^33–35^ Because PEC systems can be affected by both the electrocatalytic and surface energetics perspectives, it is vital to understand how Fe incorporation occurs in PEC systems and determine its influence on solar-driven water-splitting performance.

In this work, we present how Fe incorporation has a strong effect on the performance of Ni NP-modified n-Si photoanodes (denoted as n-Si/SiO_*x*_/NiNPs). We found that the photoanodes having the best PEC performance in the regular electrolyte performed poorly when the amount of Fe in the electrolyte was too low. Our systematic study elucidates the relationship between Fe concentration and photovoltage, highlighting how Fe plays a crucial role in both the electrochemical and *photo*electrochemical performance of the photoanodes. The results also showed that changes in the hydroxide/oxyhydroxide properties only affected the photoanodes modified with a specific NP size, as predicted by the pinch-off effect.

## Results and Discussion

### Effects of Residual Fe in the Electrolyte

n-Si/SiO_*x*_/NiNPs photoanodes, prepared by electrodeposition of randomly dispersed Ni NPs on n-Si, have shown unexpectedly high PEC performance and stability toward water oxidation despite their low Ni coverage.^14^ The general photoelectrochemical studies of this type of photoanode are provided in Section 1 of the Supporting Information. From an electrocatalytic point of view, it is well-known that Fe impurities, unintentionally present in the alkaline electrolytes (e.g., KOH-based), strongly alter the behavior of Ni species toward OER.^22,23^ For photoanodes, a previous report pointed out only the impact of Fe on the electrocatalytic properties,^36^ but its effects, specifically on PEC properties, have not been elucidated. In the following, we aim to discriminate the electrocatalytic effects induced by Fe from its electronic activity. To purify the KOH electrolytes, Fe impurities were adsorbed and removed by treating the electrolytes with a Ni­(OH)_2_ powder—a method that has been proven efficient for electrolyte purification.^22,37^ Inductively coupled plasma mass spectrometry (ICP-MS) confirmed that the nonpurified electrolyte contained Fe at a concentration of 85 μg L^–1^ (0.085 ppm), and such purification method effectively lowered it to 5 μg L^–1^ (0.005 ppm).

We first investigated the “dark” electrocatalytic properties of Ni NPs on degenerate, nonphotoactive p^+^-Si substrates (Figure 1a). Note that all of the results discussed in this section are based on NiNPs-5 (prepared with an electrodeposition charge density or ECD of 5 mC cm^–2^) since this sample showed the highest PEC performance as described in the Supporting Information. The electrodes were “activated” through 100 scans of cyclic voltammetry (CV, at 100 mV s^–1^), as regularly done in related OER literature.^36,38,39^ The voltammograms (Figure 1b) show that the electrocatalytic activity of Ni NPs decreased during CV cycling in the purified electrolyte and displayed a low current density or *j* (<1 mA cm^–2^) and a high *E*_on_ (∼1.79 V). Note that in this article, all potentials are reported versus the reversible H_2_ electrode (RHE). Conversely, for the nonpurified electrolyte, it was found that the electrocatalytic activity improved (Figure 1c) during potential cycling due to the incorporation of Fe, as previously reported.^23,25,38^ Not only did Fe affect the electrocatalytic activity toward OER but also the electrochemical response of the Ni­(OH)_2_/NiOOH layer covering Ni NPs, as shown by the change in the Ni^III^/Ni^II^ quasi-reversible redox wave,^40,41^ studied here by tracking the Ni^II^ oxidation peak (*E*_peak_) (Figure 1i). The incorporation of Fe into the Ni­(OH)_2_/NiOOH electrocatalyst is known to shift *E*_peak_ anodically (i.e., to a higher potential) due to the change in the electrochemical and electronic properties.^22,25,42^ Therefore, our observations on the Ni NPs electrocatalyst are perfectly consistent with previous literature on the OER at Ni materials. Notably, apart from the changes in electrocatalytic properties, the activation process also introduces the evolution of Ni NPs morphology revealed by the phase images from atomic force microscopy (AFM) as shown in Figure S25. The surface of Ni NPs became rougher, which could be related to the formation of the Ni­(OH)_2_/NiOOH layer. Another interesting feature observed clearly on the NiNPs-5 sample with low surface coverage was the emergence of small NPs on the Si surface after the activation. The origin of these additional NPs is ascribed to the dissolution-redeposition of Ni during the potential cycling.^18^

**Figure 1:**
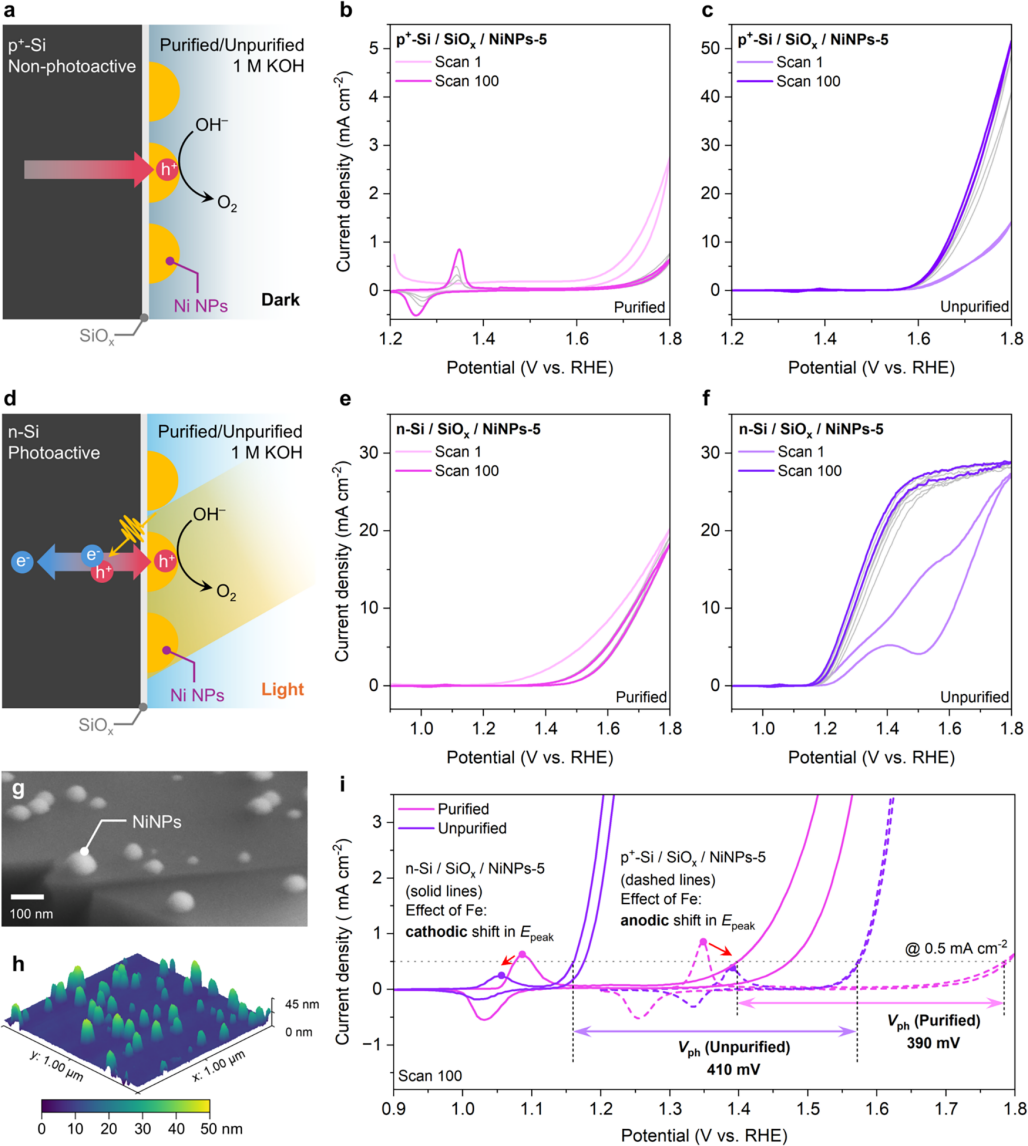
(a) Scheme of the electrocatalytic study on p^+^-Si. Sequential cyclic voltammetry (CV) scans of p^+^-Si/SiO_*x*_/NiNPs-5 in (b) purified and (c) unpurified electrolytes in the dark. (d) Scheme of the photoelectrocatalytic study on n-Si. Sequential CV scans of n-Si/SiO_*x*_/NiNPs-5 in (e) purified and (f) unpurified electrolytes under 1-sun illumination. Intermediate scans are shown in gray. (g) Tilted-view SEM and (h) 3D-AFM images of n-Si/SiO_*x*_/NiNPs-5. (i) Comparisons of CVs of p^+^-Si/SiO_*x*_/NiNPs-5 in the dark (dashed lines) and n-Si/SiO_*x*_/NiNPs-5 under 1-sun illumination (solid lines) in the purified (pink) and unpurified (violet) electrolytes after 100 scans of potential cycling. Scan rate = 100 mV s^–1^. Electrolyte: 1 M KOH. *V*_ph_: photovoltage. *E*_peak_: Ni^II^ oxidation peak.

Similar experiments were conducted on n-Si/SiO_*x*_/NiNPs photoanodes under simulated sunlight illumination (1-sun, power density = 100 mW cm^–2^, AM1.5G) as shown in Figure 1d. The PEC performance of the photoanodes was poor in the purified electrolyte, with an *E*_on_ of ∼1.4 V (Figure 1e). With Fe impurities in the unpurified electrolyte, we observed that the PEC performance improved during potential cycling, as indicated by a lower *E*_on_ and a higher slope, as shown in Figure 1f. In contradiction with the observation for samples prepared on p^+^-Si, for Ni NPs coated n-Si photoanodes (Figure 1g,h), we systematically noticed a cathodic shift of *E*_peak_ (i.e., to a lower potential) (Figure 1i). Furthermore, the photovoltage (*V*_ph_) is observed to increase slightly from 390 mV in the purified electrolyte to 410 mV in the presence of Fe impurities. This observation led us to speculate that Fe incorporation alters the energetic landscape of the junction between n-Si and Ni NPs. It is highly probable that, in previous reports, the PEC performance of similar Si photoanodes was unknowingly affected by Fe impurities in nonpurified KOH electrolytes.

### Spectroscopic and Electron Microscopy Characterization of Fe Incorporation

To verify the incorporation of Fe in the photoanodes, we employed scanning transmission electron microscopy (STEM) on photoanode cross sections and performed energy dispersive X-ray spectroscopy (EDS) elemental mapping. Due to the difficulty in characterizing such low Fe content in small NPs, to facilitate the analyses, all samples used in this section were n-Si/SiO_*x*_/NiNPs-200 (i.e., prepared by applying a large ECD of 200 mC cm^–2^). The samples were activated (i.e., subjected to 100 CV scans) in a 1 M KOH electrolyte, either purified or containing intentionally added 1.0 ppm of Fe. STEM and EDS mapping images of the control photoanode, activated in the purified electrolyte, are shown in Figure S4a–f. The presence of oxygen at the surface of Si and Ni NPs, belonging to SiO_*x*_ and Ni oxide/hydroxide species [NiO, Ni­(OH)_2_ or NiOOH] was evident from the EDS map of O (Figure S4c). The results of the photoanode activated in the electrolyte containing 1.0 ppm of Fe are shown in Figure 2a–e. The cross-section observed in the high-angle annular dark field mode (HAADF, Figure 2a) and the high-resolution bright field mode (BF, Figure 2b) showed a well-defined Si/SiO_*x*_/NiNPs interface with a SiO_*x*_ thickness of ∼1.5–2.0 nm. Ni and O EDS maps (Figure 2c,d) were similar to those of the control sample. Combining EDS Fe mapping on cross-sectioned samples (Figures 2e and S4d) with EDS (Figures 2f and S5) and XPS analysis on planar samples (Figure 2g,h), we verified the incorporation of Fe in the Ni­(OH)_2_/NiOOH shell. Detailed discussion on the elemental analysis is provided in Section 2 in the Supporting Information. Additionally, Ni phase and composition analysis allowed us to conclude that NiOOH was formed after the activation process (Figure S6), in good agreement with previous reports.^43,44^ Taken together, our characterization data reveal that activation of the n-Si/SiO_*x*_/NiNPs photoanodes in 1 M KOH induces the formation of a Ni­(OH)_2_/NiOOH shell around the Ni NPs. Importantly, when the activation is performed in the presence of Fe, the shell is doped with Fe atoms, thereby generating Ni­(Fe)­(OH)_2_/Ni­(Fe)­OOH. This is also corroborated by elemental analysis (ICP-MS after acid digestion) of photoanodes prepared and activated under different conditions, as presented in Table S7.

**Figure 2:**
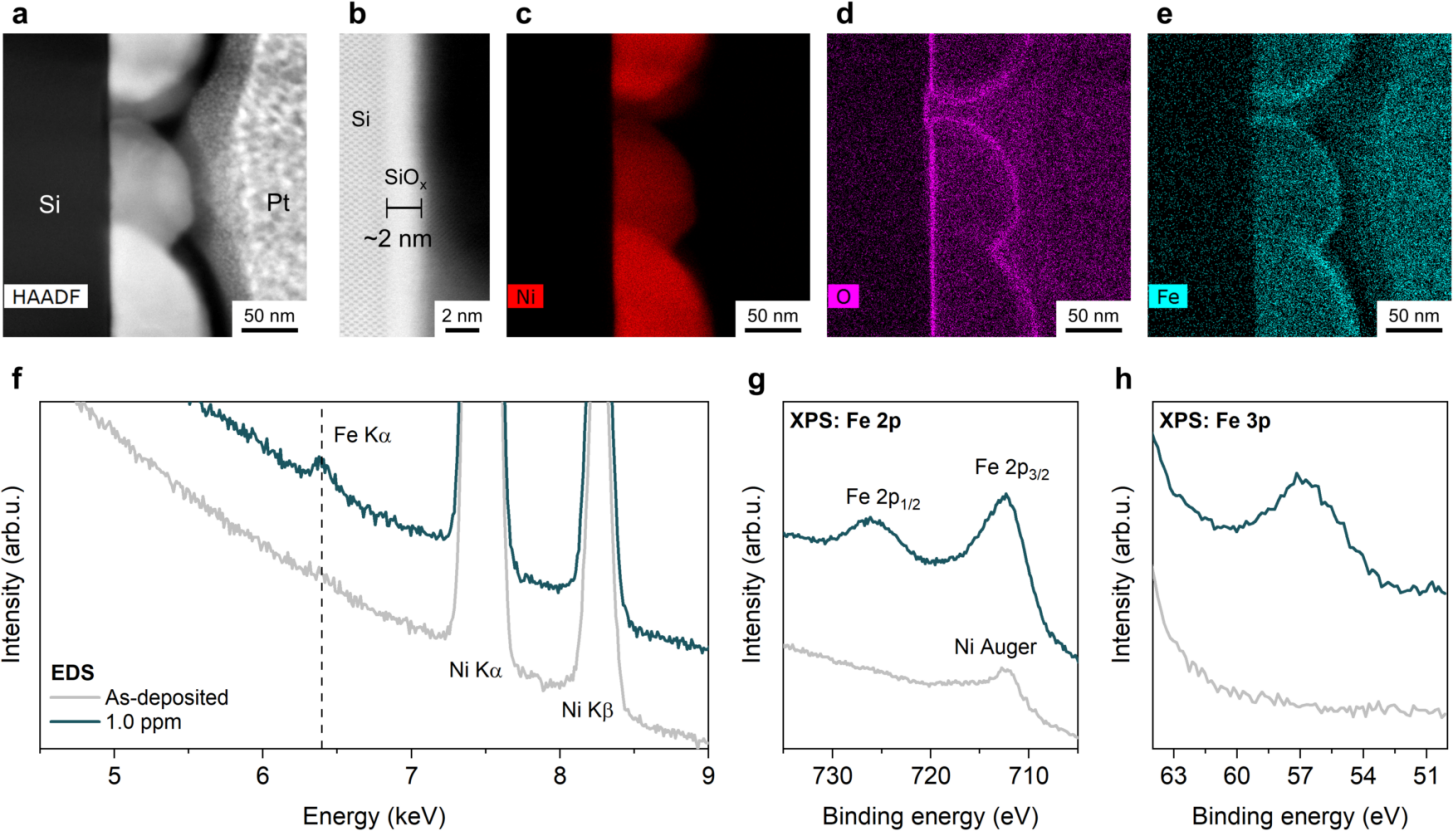
(a) HAADF-STEM, (b) high-resolution BF-STEM (displaying SiO_*x*_ thickness), and (c–e) EDS elemental mapping (Ni, O, and Fe, respectively) images of cross-sectioned n-Si/SiO_*x*_/NiNPs-200 activated in 1 M KOH electrolyte containing 1.0 ppm Fe. (f) EDS spectra and (g, h) XPS spectra of n-Si/SiO_*x*_/NiNPs-200 top surface: as-deposited (gray) and activated in the electrolyte containing 1.0 ppm Fe (dark green).

### Influence of Fe Concentration on Photoanode Properties

Next, the electrocatalytic properties of p^+^-Si/SiO_*x*_/NiNPs-5 anodes and the PEC properties of n-Si/SiO_*x*_/NiNPs-5 photoanodes were investigated as a function of Fe concentration in the electrolyte (0.1, 0.5, and 1.0 ppm). The “dark” OER behavior of Ni NPs was first studied. Figure 3a shows that *E*_on_ decreased with a higher Fe concentration, indicating that a higher density of OER active sites was generated. This result agrees well with previous reports on Ni­(OH)_2_/NiOOH OER electrocatalysts.^38,45^ A similar trend was observed for the PEC system (Figure 3b), which is naturally ascribed to better electrocatalytic activity of the Ni NPs. The effects of Fe incorporation on the bare Si electrodes (no Ni NPs, both p^+^- and n-Si) were also ruled out as there was no significant change in the electrochemical performance, as shown in Figure S26. This is also corroborated by the previously described EDS mapping of Fe, which did not indicate any Fe enrichment on the Si substrates (see Figures 2e and S4d).

**Figure 3:**
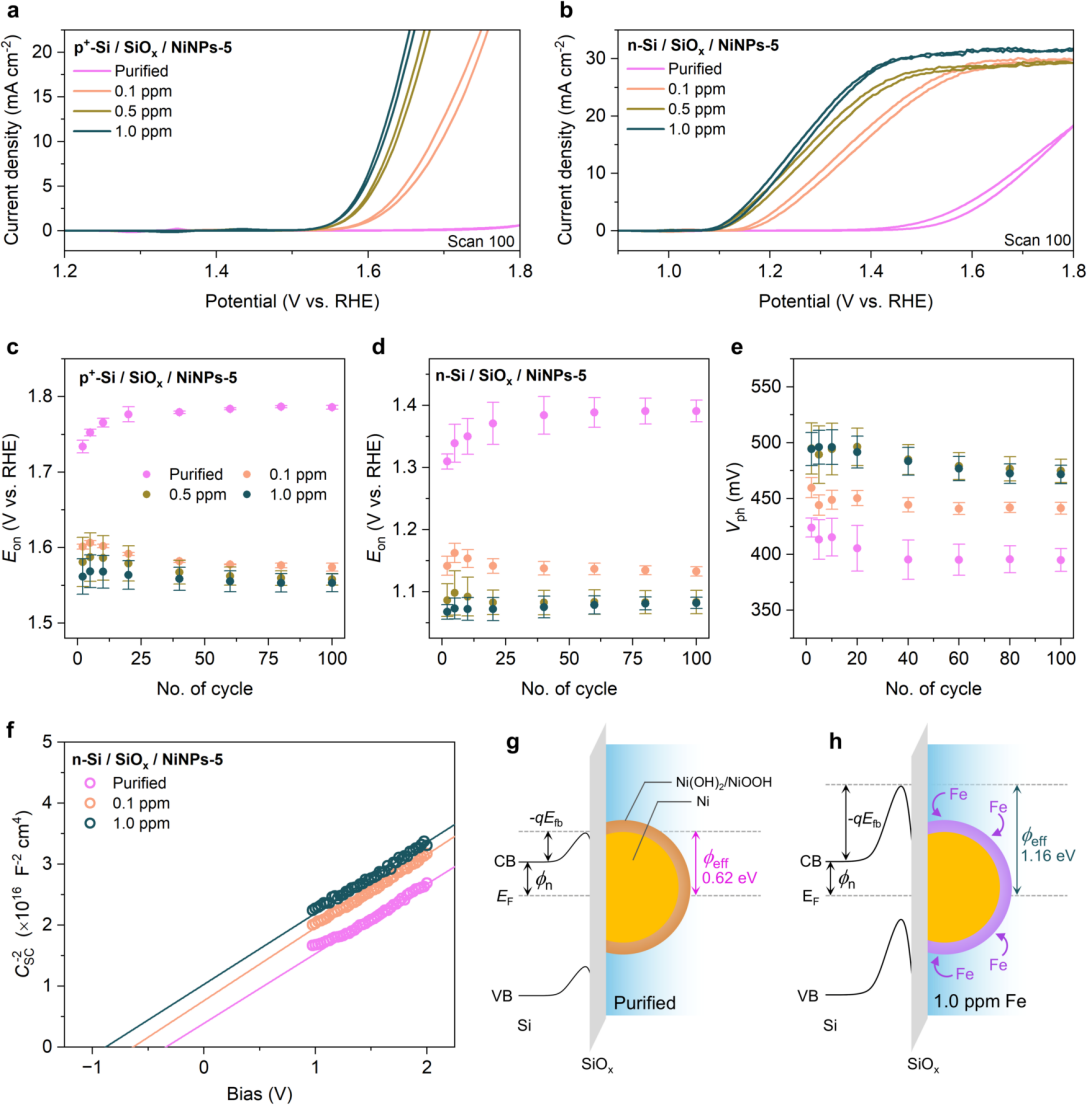
Cyclic voltammograms measured in different electrolytes for (a) p^+^-Si/SiO_*x*_/NiNPs-5 in the dark and (b) n-Si/SiO_*x*_/NiNPs-5 under 1-sun illumination. Scan rate = 100 mV s^–1^. Evolution of the onset potential during CV cycling for (c) p^+^-Si/SiO_*x*_/NiNPs-5 in the dark and (d) n-Si/SiO_*x*_/NiNPs-5 under 1-sun illumination in different electrolytes. (e) Evolution of photovoltage during CV cycling in different electrolytes. (f) Mott–Schottky plots of n-Si/SiO_*x*_/NiNPs-5 activated in different electrolytes. Schematic energy band diagrams of n-Si/SiO_*x*_/NiNPs-5 activated in (g) purified and (h) 1.0 ppm Fe-containing electrolytes displaying different barrier heights. Electrolytes: purified (pink), Fe 0.1 ppm (orange), Fe 0.5 ppm (khaki), and Fe 1.0 ppm (dark green) 1 M KOH. Symbols: *E*_on_ onset potential, *V*_ph_ photovoltage, *C*_SC_ space charge layer capacitance, *E*_fb_ flat-band potential, VB valence band, CB conduction band, *E*_F_ Fermi level, ϕ_n_ energy difference between CB and *E*_F_, and ϕ_eff_ effective barrier height.

To elucidate the effects of Fe incorporation on the PEC properties, we calculated the *V*_ph_ generated by n-Si/SiO_*x*_/NiNPs-5 photoanodes in the 1 M KOH electrolytes with varying Fe concentrations. It was found that *V*_ph_ systematically increased with the Fe concentration, as shown in Table S8. It has been previously reported that similar MIS Si photoanodes can provide more than 450 mV of photovoltage, with values as high as 500 mV, as summarized in Table S11, agreeing with our results of 480 mV obtained with the 1.0 ppm Fe-containing electrolyte.^18,20^ However, without the presence of Fe, the photovoltage we measured significantly dropped to ∼390 mV. This suggests that Fe incorporation into the Ni­(OH)_2_/NiOOH shell on Ni NPs affected both the electrocatalytic properties and the junction energetics, including photovoltaic properties, of the n-Si/SiO_*x*_/NiNPs-5 photoanodes. The synergy between these effects led to drastically improved PEC performance.

The CV evolution during the activation of n-Si/SiO_*x*_/NiNPs-5 photoanodes in electrolytes with varying Fe concentrations, displayed in Figure S27, presents different notable characteristics at an early stage. With a lower amount of Fe in the electrolyte (unpurified in Figure 1f and 0.1 ppm in Figure S27a), it appears that the progression of Fe incorporation could be observed for the first few potential scans, but this feature could not be noticed at higher Fe concentrations. We postulate that Fe is rapidly incorporated into Ni­(OH)_2_/NiOOH structure without the need for external bias if Fe concentration is sufficiently high.^22,23^ We also tracked *E*_on_ for the p^+^-Si/SiO_*x*_/NiNPs anodes and their photoactive n-Si/SiO_*x*_/NiNPs counterparts and calculated *V*_ph_ as a function of the scan number during activation (Figure 3c–e). In the Fe-containing electrolytes, it was found that *V*_ph_ was already high during the early scans, even for a low amount of Fe (0.1 ppm) when compared to that in the purified electrolyte. We thus conclude that the presence of Fe in the electrolyte has a quasi-immediate effect on the photovoltage even though the incorporation process might not have reached equilibrium, as discussed in Section 3 of the Supporting Information.

We then investigated the solid-state behavior of the photoanode with and without Fe incorporation via impedance spectroscopy and Mott–Schottky (MS) analysis. The method for determining the space charge layer capacitance (*C*_SC_) and the detailed analysis are presented in Section 4 of the Supporting Information. The MS plots, presented in Figure 3f, reveal that the effective barrier height (ϕ_eff_) systematically increased with the concentration of Fe in the electrolyte, from 0.62 to 1.16 eV (Figure 3g,h and Table S2). These results support that the junction energetic landscape changes upon the incorporation of Fe. Taken together with similar MS analysis on a homogeneous n-Si/SiO_*x*_/Ni film (Figure S10 and Table S3), it is further elaborated that Fe located near the proximity of the n-Si/SiO_*x*_ interface (i.e., in n-Si/SiO_*x*_/NiNPs-5) exerts a greater influence on ϕ_eff_ when compared to Fe located far from this interface (i.e., in n-Si/SiO_*x*_/Ni film).

The effects of Fe impurities on the photoanodes’ stability in operation were also investigated, as shown and discussed in Section 5 in the Supporting Information. In summary, we did not observe significant differences in the photoanodes’ stability with Ni NPs as catalysts. Conversely, when an ultrathin Ni thin film (5 nm) was used instead, the stability clearly became poorer in the purified electrolyte compared to that in the Fe-containing electrolyte. Because a large extent of ultrathin Ni likely exists in the form of NiOOH under PEC conditions, this result indicates that Fe stabilizes the NiOOH phase. However, for n-Si/SiO_*x*_/NiNPs, the long-term activity is likely controlled by the stability of the n-Si/SiO_*x*_ and SiO_*x*_/Ni interfaces under operating conditions. Strategically, a more chemically stable oxide layer could be employed instead of the native SiO_*x*_ or as an additional layer (e.g., TiO_2_) to further extend the photoanode stability as reported in the literature.^12,13,46^

### Effects of Ni NP Size and Coverage

In the previous experiments, the Fe incorporation effects were investigated for the smallest Ni NPs (with a diameter of ∼50 nm), which are expected to undergo the most pronounced pinch-off effect.^10,17,18^ However, we also analyzed larger and denser Ni NPs, prepared with ECDs of 50 and 200 mC cm^–2^. As pinch-off depends on the size of the metal contacts, these n-Si/SiO_*x*_/NiNPs would normally lead to lower *V*_ph_ and higher *E*_on_ due to the weaker pinch-off (or its absence). It is also worth noting that partial shading of n-Si caused by higher surface coverage at a higher ECD can potentially decrease the photovoltage due to lower photon flux reaching n-Si, thus leading to a lower density of photogenerated holes. This aspect is discussed in Section 6 of the Supporting Information. In short, partial shading can partly reduce the system photovoltage, but only to a small extent (<50 mV). Therefore, as pointed out in other works, other factors (i.e., pinch off,^18^ partial charge screening,^21^ or reverse current^20^) should account for the major change in photovoltage related to the NP size and surface coverage. Considering the “dark” electrocatalytic properties, Figures S28a and S29a show that similar trends were obtained for p^+^-Si/SiO_*x*_/NiNPs-50 and p^+^-Si/SiO_*x*_/NiNPs-200 when varying Fe concentration. As shown in Figure S30a (top panel), after activation in the purified electrolyte, *E*_peak_ (i.e., the Ni^II^ oxidation peak) was found at the same potential (∼1.35 V) for all p^+^-Si/SiO_*x*_/NiNPs anodes, showing that the composition of the Ni­(OH)_2_/NiOOH was electrochemically equivalent regardless of the NiNPs size and loading. As the amount of Fe increased, *E*_peak_ shifted anodically for all p^+^-Si/SiO_*x*_/NiNPs. The smallest NP size displayed the largest shift, suggesting a higher proportion of Fe incorporated compared to larger NPs. On the contrary, *E*_peak_ for the photoactive n-Si/SiO_*x*_/NiNPs electrodes was located at more cathodic potential (∼1.21 V or lower) (Figure S30b), due to the *V*_ph_ generation by these photoanodes (as previously shown in Figure 1i). Interestingly, while for n-Si/SiO_*x*_/NiNPs-5, both *E*_peak_ and *E*_on_ shifted cathodically with the increasing amount of Fe, for the larger particles in n-Si/SiO_*x*_/NiNPs-50 and n-Si/SiO_*x*_/NiNPs-200, the trend was different as *E*_peak_ shifted anodically whereas *E*_on_ shifted cathodically. *V*_ph_ values, calculated for all ECDs using data shown in Figures S28 and S29, are reported in Tables S9 and S10. These data show that, for ECDs of 50 and 200 mC cm^–2^, there was no significant change in the *V*_ph_ value over the change in Fe concentration.

From these results, we determine that the effects of Fe incorporation on PEC performance also depend on the size of the NPs and their dispersion. This can be explained by the pinch-off effect theory, where the changes caused by Ni­(Fe)­(OH)_2_/Ni­(Fe)­OOH modification only affect the photoanodes when the Ni NPs are below a certain size regime.^18,47^ As illustrated in Figure 4a, the photogenerated minority carriers are effectively pulled toward the surface (by the larger band bending) only when the NPs are smaller than the depletion region of the high barrier area arising from the high work function shell. This leads to higher ϕ_eff_ (Figure 4b) and *V*_ph_ (Figure 4e). For the large NPs (Figure 4c), the change in the electronic properties of Ni­(Fe)­(OH)_2_/Ni­(Fe)­OOH still exists; however, due to their size, pinch-off becomes significantly weaker, and only the change in the electrocatalytic properties can be experimentally observed. This is further supported by the Mott–Schottky analysis on n-Si/SiO_*x*_/NiNPs-200 (Section 3 in the Supporting Information, Figure S11, and Table S4), which revealed no changes in surface energetics. As summarized in Figure 4d,e, ϕ_eff_ and *V*_ph_ of the photoanodes with larger NPs did not significantly change when Fe was incorporated.

**Figure 4:**
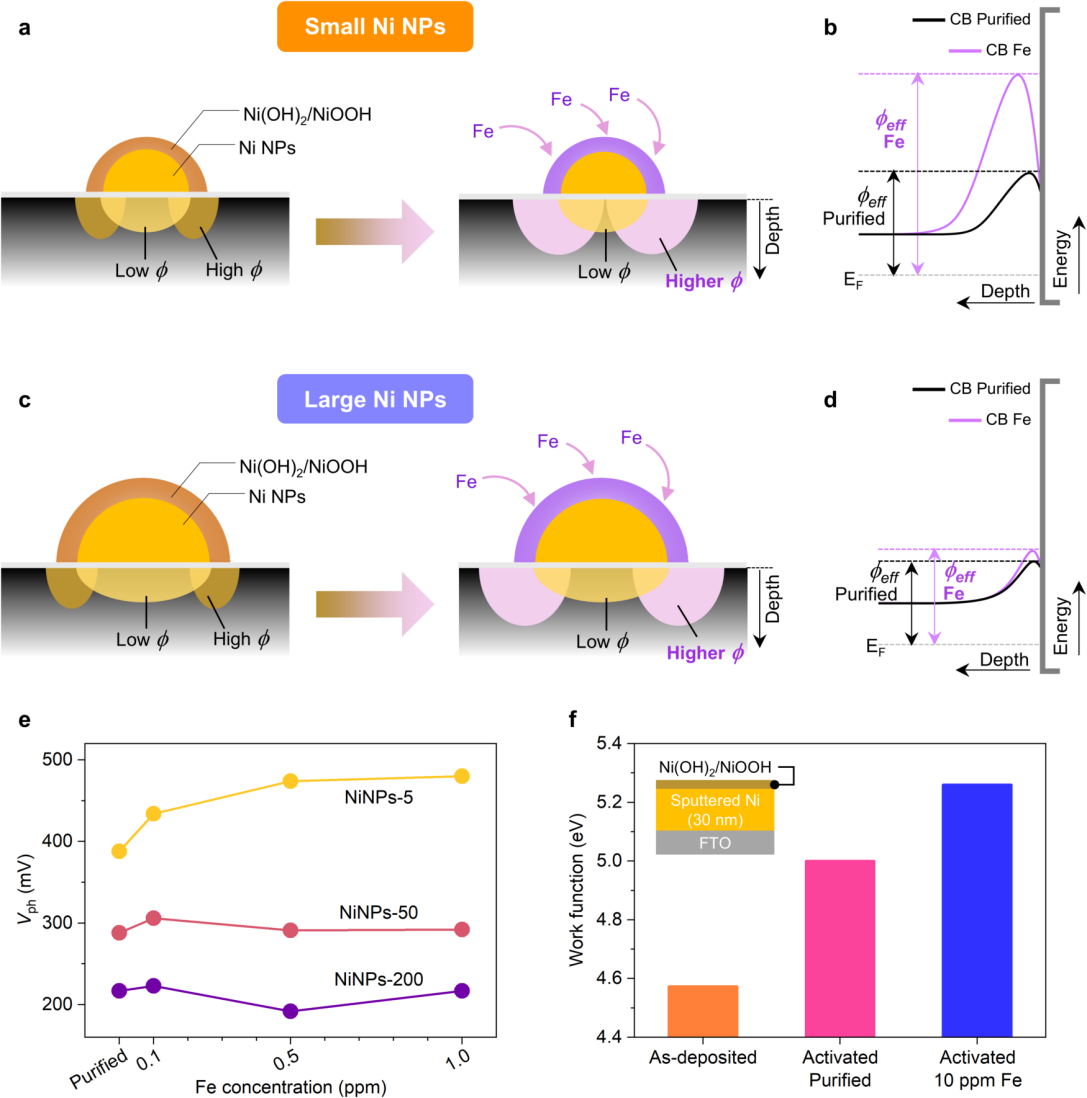
(a, c) Schemes illustrating depletion regions and (b, d) band bending (only depicting the conduction band CB) near the n-Si/SiO_*x*_/Ni interface for junctions in purified or Fe-containing electrolyte: (a, b) small Ni NPs and (c, d) large Ni NPs. (e) Photovoltage of different sizes of Ni NPs in electrolytes with varying Fe concentration. (f) Work functions measured on as-deposited and activated Ni films on FTO glass substrate. ϕ, ϕ_eff_, and *V*_ph_ refer to local barrier height, effective barrier height, and photovoltage, respectively.

It is known that the space charge layer or depletion width increases with a higher built-in potential (i.e., a higher barrier height).^48^ Thus, from our results, smaller Ni NPs possess a proportionately larger depletion width (relative to the particle size) when compared to larger Ni NPs. The incorporation of Fe into Ni­(OH)_2_/NiOOH effectively widens the depletion region only on small Ni NPs, leading to a better PEC performance. The latter can be attributed to the higher work function of Ni­(Fe)­OOH when compared with NiOOH (thus producing a larger ϕ_eff_ surrounding the NP and causing more band bending). To measure the work function of these coatings and validate our hypothesis, we deposited Ni thin films by magnetron sputtering onto fluorine-doped tin oxide (FTO) glass substrates (see Figure 4f, inset), subjected them to different activation conditions, and performed Kelvin probe measurements. The work function measured on the as-deposited sample was 4.57 eV. After activation in the purified electrolyte, the work function increased to 5.00 eV, attributable to the formation of Ni­(OH)_2_/NiOOH.^49^ Importantly, the activation in the presence of Fe impurities (10 ppm Fe) further raised the value to 5.26 eV. We have also performed a work function mapping of Ni NPs via Kelvin probe force microscopy (KPFM) as discussed in Section 7 of the Supporting Information. This series of measurements confirmed the trend in Figure 4f.

Based on the pinch-off effect, the increase in the work function caused by Fe incorporation into Ni­(OH)_2_/NiOOH could account for a ∼0.5 eV shift in ϕ_eff_ on n-Si/SiO_*x*_/NiNPs-5. To provide a deeper understanding of our findings, we performed an analysis based on the theoretical model introduced by Tung.^17,50^ The calculations, provided in Section 8 of the Supporting Information, corroborate that the change in the NiOOH work function induces a significant shift in ϕ_eff_. Nevertheless, we add that several recent works have suggested alternative explanations for rationalizing the performance of inhomogeneous MIS photoelectrodes. Hemmerling et al. have correlated the improvement in the photovoltage between the homogeneous and inhomogeneous junction to the presence of a thin insulator oxide (particularly SiO_*x*_) and the dark recombination current based on the metal–semiconductor contact.^20,51^ King et al. have rationalized the same morphology-photovoltage relationship based on the charge screening and potential drop at the interface.^21^ However, we remark that these contributions exclude the impact of the surrounding shell, whose presence and effects on the energetics and performance are clearly revealed by our study. It is still possible that there could be interrelationships among different models.

### Effects of Fe Impurities on the Work Function of the Hydroxide/Oxyhydroxide Layer

Combining our (1) (photo)­electrochemical study, (2) STEM-EDS mapping, (3) MS analysis, and (4) work function measurements, we conclude that Fe incorporation effectively alters the surface energetics of inhomogeneous MIS photoanodes. The larger ϕ_eff_, caused by the higher work function of Ni­(Fe)­(OH)_2_/Ni­(Fe)­OOH, leads to more pronounced band bending. To exploit this phenomenon in a solar-driven PEC system, small NPs are required, further lending support to the pinch-off theory. The elucidation of the surface energetics and electronic properties of Ni­(Fe)­OOH is rare in the literature due to the convoluted effects of Fe on both the electrocatalytic and photoelectrochemical properties, as highlighted in this work. However, the few available reports from other groups have also indicated that the intentional coating of NiFe oxy­(hydroxide) layer leads to a larger band bending on homogeneous MIS Si photoanodes.^52,53^ Shen et al. also measured the work functions of NiOOH and Ni­(Fe)­OOH on n-Si/SiO_*x*_/CoO_*x*_/NiO_*x*_ to be 6.21 and 6.41 eV, respectively, further corroborating our mechanism.^53^

In order to investigate the influence of Fe substitution in NiOOH at the atomic level, we performed a computational study at the spin-polarized DFT+*U* level of theory.^54^ The method and the related discussion are described in detail in Section 9 of the Supporting Information. While the computed work function of NiOOH was found to be 4.8 eV, we found that, at a potential of 1.5 V vs RHE and with the surface fully substituted by Fe, the work function of the oxyhydroxide could increase up to 6.5 eV. Considering an Fe surface concentration of ∼50% in the model and an overall Fe content of ∼25% as a model representative of the experimentally obtained phase, we found a work function increase of 1 eV compared to unsubstituted NiOOH. These calculations identified the origin of the increased work function of the oxyhydroxide to the combined effects of Fe present in the surface which, in turn, causes a decrease in the number of hydrogen atoms on the surface. This surface hydrogen coverage causes a substantial change in the work function observed experimentally. The higher increase found by the calculations compared with the experimental measurements suggests that either the average surface concentration of Fe is slightly lower or the hydrogen coverage is slightly higher in reality.

Importantly, it is worth emphasizing that the Fe-induced effects demonstrated in this work could be potentially applied to other MIS systems employing different metals or semiconductors. To elaborate on this perspective, we extended our study to Si photoanodes modified with another transition metal catalyst, i.e., Co NPs. Interestingly, similar effects found with Ni NPs were also observed with Co NPs. The detailed experiment and discussion can be found in Section 10 of the Supporting Information. Crucially, this result further supports the possible generalization of Fe effects to other systems.

### Fe Leaching: Reversing Fe Incorporation

To go a step further, we investigated whether the incorporation of Fe could be reversible. Both photoanodes and anodes prepared with different ECDs were activated in an iron-containing electrolyte and subsequently cycled in the purified electrolyte. Detailed analysis and discussion are given in Section 11 in the Supporting Information. Among all measured anodes, only the one containing the small Ni NPs deposited on n-Si (n-Si/SiO_*x*_/NiNPs-5) presented an anodic shift in *E*_peak_ upon Fe leaching as shown in Figure S22b, which is the reverse of the changes during Fe incorporation of this sample (cathodic shift of *E*_peak_, see Figure S31), further supporting our mechanism. Figure S32 shows the evolution of *E*_on_ and *V*_ph_ as a function of the CV scans during Fe leaching. This plot reveals that *V*_ph_ dropped significantly for n-Si/SiO_*x*_/NiNPs-5, with a decrease of 90 mV within the first 50 potential scans and then by 40 mV over the rest of the measurement (150 scans). Meanwhile, there were negligible changes in *V*_ph_ for n-Si/SiO_*x*_/NiNPs-50 and n-Si/SiO_*x*_/NiNPs-200 as Fe leached out. To summarize, these results support the mechanism (Figure 4) explaining how this type of Si photoanode behaves under the formation of Ni­(Fe)­(OH)_2_/Ni­(Fe)­OOH and the dependency on the Ni NPs size. Overall, our results point out that sufficiently small Ni NPs are required to exploit the synergetic enhancements of electrocatalytic properties and surface energetics caused by Fe doping. Furthermore, the presence of Fe is also necessary in the electrolyte during operation to prevent Fe leaching and sustain the high PEC performance.

## Conclusions

In summary, we have unraveled the effects of Fe incorporation on the PEC performance of inhomogeneous MIS Si photoanodes modified with Ni NPs. In addition to the improvement in the OER electrocatalytic properties of Ni­(OH)_2_/NiOOH, we conclusively demonstrated that the inclusion of Fe also strongly affects the photoelectrochemical properties, specifically, the junction energetics and the resulting photovoltage. Importantly, the high photovoltage of more than 450 mV, which has been previously reported in high-performance n-Si/SiO_*x*_/NiNPs, can only be achieved with the adventitious or intentional presence of Fe ions both in the outer shell of the NPs and in the electrolyte. Moreover, the PEC enhancement, caused by Fe incorporation, also depends on the size of the Ni NPs. We have shown that only sufficiently small NPs can benefit from it. Our characterizations showed that Fe is incorporated into the outer shell surrounding Ni NPs, forming Ni­(Fe)­(OH)_2_/Ni­(Fe)­OOH, which promotes a higher effective barrier and larger band bending, resulting in a higher photovoltage. The effects of Fe are also reversible; operating Fe-incorporated photoanodes in purified electrolyte results in Fe leaching and a drastic drop of ∼100 mV in photovoltage. In the future, it would be compelling to assess whether Fe leaches out from the bulk or only from the surface since it has been reported that Fe can still remain inside the bulk Ni­(OH)_2_/NiOOH during/after leaching.^24,38^ The last experiments also indicate that Fe is required in the electrolyte itself during the OER to maintain the high photovoltage. Our study emphasizes the crucial role of Fe in both the preparation and operation of high-performance PEC systems for solar-driven water-splitting applications. This work also highlights the fundamental importance of systematically evaluating the performance of semiconductor-based photoelectrodes in Fe-containing and purified electrolytes.

## Methods

### Materials and Reagents

All silicon (Si) wafers were purchased from Latech Scientific Supply and single-side polished. n-Si (⟨100⟩, phosphorus-dope) and p^+^-Si (⟨100⟩, boron-doped) have resistivities of 1–10 Ω cm and 0.001–0.009 Ω cm, respectively. Nickel nitrate hexahydrate (Ni­(NO)_3_·6H_2_O, 99.999%), iron nitrate nonahydrate (Fe­(NO_3_)_3_·9H_2_O, ≥99.95%), buffered oxide etchant (BOE, 6:1), eutectic gallium–indium alloy (EGaIn), boric acid (H_3_BO_3_, ≥99.5%), nickel chloride (NiCl_2_, 99.99%), sulfuric acid (H_2_SO_4_, 98%), cobalt sulfate heptahydrate (CoSO_4_·7H_2_O, ≥99.9%), and hydrogen peroxide solution (H_2_O_2_, 30%(w/w) in H_2_O) were purchased from Sigma-Aldrich. Potassium hydroxide (KOH, AR grade) was purchased from UNIVAR. Acetone (AR grade) and 2-propanol (AR grade) were purchased from Carlo Erba.

### Purification/Preparation of the Electrolytes

Fe removal process was done by using Ni­(OH)_2_ as the Fe adsorbent.^22^ Ni­(OH)_2_ was prepared by dissolving 2 mg of Ni­(NO_3_)_3_·6H_2_O in ultrapure Milli-Q water (18 MW cm) and then mixing it with 20 mL of a 1 M KOH solution (prepared from ultrapure water). The precipitate was then extracted by filtration. Ni­(OH)_2_ was then dispersed in the unpurified electrolyte by stirring for 3 h and left for ∼12 h undisturbed before the experiment. Ni­(OH)_2_ precipitate was then filtered out from the electrolyte using filter paper prior to usage. A hydrophilic-PTFE syringe filter (0.22 μm) was then used to further remove the remaining Ni­(OH)_2_ particles in the electrolyte and thus avoid the presence of Ni impurity in the electrolyte.^37^ The electrolyte that went through the Fe removal process is referred to as “purified electrolyte.” The untreated electrolyte containing Fe impurities from the KOH reagent is referred to as “unpurified electrolyte.” Fe­(NO_3_)_3_ was used as the source of Fe^3+^ in the purified electrolyte to intentionally control the concentration of Fe^3+^. These electrolytes are denoted by the concentration of Fe^3+^ added, i.e., 0.1, 0.5, and 1.0 ppm. All of the electrolytes used in this study for water oxidation were 1 M KOH (pH 14) unless otherwise specified.

### Electrodeposition of Ni Nanoparticles on Si Substrates

Si wafers were cleaned by sonicating them in Liquinox, deionized water, acetone, and isopropyl alcohol, each for 10 min in that order. Then, the Si wafer was cut into small pieces (∼0.5 × 0.5 cm^2^). The unpolished backside was scratched and attached to a copper wire with EGaIn and conductive silver paint. After that, the backside and the edges of each electrode were sealed with epoxy resin. The same procedure was applied to both n-Si and p^+^-Si wafers. The exposed surface was photographed, and the size of the active area was determined by using ImageJ software. Ni-plating electrolyte contained 20 mM NiCl_2_ and 0.1 M H_3_BO_3_. The deposition potential was −1.5 V versus saturated calomel electrode (SCE) with various electrodeposition charge densities (ECD). Prior to electrodeposition, the electrode was treated in BOE (6:1) for 30 s. Each electrode is denoted as ‘Si/SiO_*x*_/NiNPs-*X*’ where *X* is the ECD in mC cm^–2^.

### Electrodeposition of Co Nanoparticles on Si Substrates

Prior to electrodeposition, electrodes were prepared using the same procedure as that mentioned above. The plating electrolyte of Co contained 0.1 M CoSO_4_·7H_2_O and 0.1 M H_3_BO_3_. The deposition potential was −1.4 V vs SCE with various ECD values. Each electrode is also denoted as ‘Si/SiO_*x*_/CoNPs-X’ where X is the ECD in mC cm^–2^.

### Preparation of the Sputtered Ni Film

Si and FTO surfaces were cleaned using the procedure described previously. Si was then oxidized in piranha solution (3:1 v/v concentrated H_2_SO_4_:30% H_2_O_2_) at 105 °C for 30 min. The Ni film was deposited on the substrate by sputtering with a Leica EM ACE600 coating system. (The thickness was monitored *in situ* by a quartz crystal microbalance). During sputtering, the Ar gas pressure was 2 × 10^–2^ mbar and the current was 100 mA.

### Microscopy and Elemental Characterizations

Morphology of the Si photoanodes was studied by scanning electron microscopy (SEM) using a JEOL JSM-7610F system operated at a low landing energy (1.0–2.0 keV, in gentle beam mode). The energy dispersive X-ray spectroscopy (EDS) spectra were acquired using a long acquisition time (approximately 45 min for each sample) at low magnification (500×). This long acquisition time was required to enable the detection of iron on the surface.

For the preparation of the samples for cross-sectional scanning transmission electron microscopy (STEM), lamellae were prepared using a Xe plasma FIB instrument (Tescan Amber X). Prior to ion beam exposure, a protective Pt layer was deposited via electron beam to preserve the integrity of the photoanodes’ surfaces. Subsequently, standard ion beam Pt deposition and lift-out procedures were carried out. The samples were then thinned to electron transparency to enable STEM-EDS and STEM imaging.

STEM studies were performed on Titan Themis 200 TEM/STEM microscopes, equipped with a spherical aberration corrector on the probe and working at 200 kV. This microscope was equipped with the ″super X″ four-detector system (0.8 sr detection angle). For these experiments, the probe current was about 70 pA with a half angle of convergence of 17 mrad. To achieve sufficient resolution on Fe and lower the detection limit of this element, STEM-EDS chemical maps were carried out over very long acquisition times of more than 1 h (up to 2 h). Acquisition was sequential, with correction of any possible drift every 5 s by cross correlation.

X-ray photoelectron spectroscopy (XPS) was performed by using a K-Alpha apparatus (ThermoFisher Scientific) with a monochromatized Al–Kα source (1486.7 eV) and a 400 μm X-ray spot size. The full spectra (0–1150 eV) were obtained with a constant pass energy of 200 eV while high-resolution spectra were recorded with a constant pass energy of 40 eV. Charge neutralization was applied during the analysis.

Iron concentrations were determined using a triple quadrupole inductively coupled plasma mass spectrometer (iCAP TQ-ICP-MS, ThermoFisher Scientific). The ^57^Fe isotope (natural abundance 2.12%) was selected in Kinetic Energy Discrimination (KED) mode to minimize polyatomic interferences. Rhodium (1 μg L^–1^, single-element standard solution, PlasmaCAL, SCP Science) was used as an internal standard to monitor instrumental stability and to correct for potential signal drift. Quality control was verified using the certified reference material SLRS-6 (River Water for Trace Metals, National Research Council Canada),^55^ with a certified Fe concentration of 84.3 ± 3.6 μg L^–1^ and a measured value of 82.6 ± 1.29 μg L^–1^, thus confirming method accuracy and precision. For the quantification of Fe concentration in the electrolyte, the solutions (1 M KOH matrix) were acidified to pH 1 using HNO_3_, and then diluted 40-fold with ultrapure water (Milli-Q IQ 7000, Merck Millipore) prior to analysis. Fe concentrations were quantified by the standard-addition method to ensure matrix-matched calibration. Additions of 0, 0.1, 0.3, 0.5, and 0.7 μg L^–1^ Fe (PlasmaCAL, SCP Science) were made for the purified electrolyte; and 0, 1, 2, 3, and 4 μg L^–1^ for the unpurified electrolyte.

For the quantification of Fe content on Si substrates, the samples were dissolved by immersion for 3 h at 70 °C in 10 mL of 1 M HNO_3_ (sub-boiling distilled, Savillex) using 15 mL polypropylene tubes (DigiTUBEs, Analytichem) that had been precleaned with high-purity HNO_3_, obtained by sub-boiling distillation (DST-1000 Acid Purification System, Savillex). Fe concentrations were determined by external calibration using reference solutions of 0, 0.45, 0.9, 1.5, and 2 μg L^–1^ Fe.

Atomic force microscopy (AFM) was done on a Bruker MultiMode 8 with the ScanAsyst air method. The AFM probe was a Bruker model SCANASYST-AIR (70 kHz, 0.4 N m^–1^, 115 μm length). Gwyddion program was used to analyze AFM data.

### Electrochemical and Photoelectrochemical Measurements

All electrochemical (EC) and photoelectrochemical (PEC) experiments were conducted in a single-compartment PTFE PEC cell (Ossila) fitted with a quartz window and measured with a PalmSens4 potentiostat. The reference electrode and the counter electrode were Hg/HgO (1 M KOH) and Pt wire, respectively. The potential was converted to the reversible hydrogen electrode (RHE) scale using eq 1. The light illumination was provided by a Sciencetech SF150B solar simulator equipped with an AM1.5G filter. The light intensity was calibrated to 100 mW cm^–2^ (1 sun) using an InfinityPV CalCell photodiode reference device before the experiments. All reported potentials were intentionally not corrected for the *iR* drop. The scanning rate for all cyclic voltammetry (CV) experiments was set at 100 mV s^–1^ unless otherwise specified.

1 $$E_{\mathrm{RHE}}=E_{\mathrm{Hg}/\mathrm{HgO}}+0.098+\left(0.059^{\;}\times \mathrm{pH}\right)$$

### Solid-State Impedance Measurements

The exposed area of the photoanode was covered with Ag paste, attached to a Cu wire, and used as the front contact. The front and back contacts were connected to the counter/reference and working leads, respectively. The impedance measurements were conducted with a BioLogic SP-300 potentiostat. The impedance was measured from 0–2 V in the dark. At each potential, the impedance spectrum was recorded between 400 Hz and 4 MHz with a modulation amplitude of 10 mV. EC-lab (BioLogic) was employed to fit and analyze Nyquist plots to obtain the capacitance of the space charge layer (*C*_sc_). The detailed analysis for Mott–Schottky plots is discussed in Section 3 of the Supporting Information.

### Work Function Measurements

A sputtered Ni metal thin film with a thickness of 30 nm deposited on FTO was employed as a model system to investigate the effect of Fe incorporation. The samples were FTO/Ni surfaces: as-deposited, activated in the purified electrolyte, and activated in the electrolyte containing 10 ppm of Fe (high Fe concentration to maximize the effect on the planar structure). The contact potential difference measurement was conducted using a Kelvin probe setup from Besocke Delta Phi including a Kelvin probe made of a gold grid of 2.5 mm diameter (Kelvin probe S) and the Kelvin control 07. The work functions of the different samples were determined by comparing with highly oriented pyrolytic graphite (HOPG) used for calibration with a reference work function of 4.5 eV.

Kelvin probe microscopy (KPFM) measurements were obtained using a Bruker Dimension Icon AFM instrument in amplitude modulation (AM) mode. Topography and contact potential difference (CPD) were acquired by using the dual-pass technique. During the first pass, the topography was recorded in tapping mode, while during the second pass (lift mode), the CPD was collected by applying an AC voltage between the tip and the sample at the resonance frequency of the cantilever. The images (5 μm × 5 μm, 1024 pixels × 1024 pixels) were acquired by using a silicon probe with a thin layer of Pt–Ir coating (SCM-PIT-V2, Bruker) at a scan rate of 0.1 Hz and a lift height of 50 nm. The CPD images were then converted to work function images using the following equation: *V*_DC_ = *V*_CPD_ = (Φ_sample_ – Φ_tip_)/*e*, where *V*_DC_ is the DC voltage applied by the system to nullify the CPD voltage (*V*_CPD_) between the sample and the tip, Φ_sample_ and Φ_tip_ are the work functions of the sample and the tip, and *e* is the elementary charge. The work function of the tip was obtained with a standard calibration sample of patterned Au–Si–Al structures (Bruker).

## Supplementary Material



## Data Availability

All relevant geometries used in the computational analysis can be consulted on nomd-lab.eu.^56^

## Notes

Supplementary notes on preparation of silicon MIS photoanodes by Ni electrodeposition, elemental analysis, evolution of onset potential and photovoltage, Mott–Schottky analysis, detail on Fe leaching experiment, long-term stability of photoanodes, theoretical photovoltage calculation, spatial work function mapping, analysis on Tung’s model and pinch-off effect, computational details and discussion on NiOOH and Ni­(Fe)­OOH properties, Fe effects on Co NPs modified n-Si photoanode, supplementary tables, and supplementary figures. (PDF)
