# Correction to “Unraveling the Effects of Fe Incorporation on High-Performance Water-Splitting Photoanodes”

**DOI:** 10.1021/jacs.6c11698

**Published:** 2026-06-30

**Authors:** Kanokwan Klahan, Gilles Patriarche, Stephan Steinmann, Laureline Treps, Gilles Pécastaings, Osmane Camara, Rüdiger-A. Eichel, Sylvain Chambon, Patrick Garrigue, Cécile Bossy, Sareeya Bureekaew, Gabriel Loget, Pichaya Pattanasattayavong

There were some typographical errors in the original article, which should be corrected as follows.

In the Methods: Materials and Reagents section, “Ni­(NO)_3_·6H_2_O” should read “Ni­(NO_3_)_2_·6H_2_O”.

In the Methods: Purification/Preparation of the Electrolytes section, “by dissolving 2 mg of Ni­(NO_3_)_3_·6H_2_O in ultrapure Milli-Q water (18 MW cm)” should read “by dissolving 2 g of Ni­(NO_3_)_2_·6H_2_O in 4 mL of ultrapure Milli-Q water (18 MΩ cm)”.

The experiments were performed using the correct reagent and quantity. These corrections do not affect the results, discussion, or conclusions of the original article.

