# Supplementary material for: Unraveling the Effects of Fe Incorporation on High-Performance Water-Splitting Photoanodes
Source: J Am Chem Soc. 2026 Jan 27;148(5):5508–19. doi: 10.1021/jacs.5c19704 (PMC12903853; doi:10.1021/jacs.5c19704)
Supplement: Supplementary file 1 [file ja5c19704_si_001.pdf]

Supporting Information

Unraveling the Effects of Fe-Incorporation on High-Performance Water-Splitting Photoanodes

Kanokwan Klahan,[a,b] Gilles Patriarche,[c] Stephan Steinmann,[d] Laureline Treps,[d] Gilles Pécastaings,[e] Osmane Camara,[f] Rüdiger-A. Eichel,[f] Sylvain Chambon,[g] Patrick Garrigue,[b] Cécile Bossy,[h] Sareeya Bureekaew,[i] Gabriel Loget,[b,*] and Pichaya Pattanasattayavong[a,*]

[a] Department of Materials Science and Engineering, School of Molecular Science and Engineering, Vidyasirimedhi Institute of Science and Technology (VISTEC), Rayong 21210, Thailand

[b] University of Bordeaux, Bordeaux INP, ISM, UMR CNRS 5255, Pessac 33607, France

[c] Centre de Nanosciences et de Nanotechnologies, Université Paris-Saclay, CNRS, Bld Thomas Gobert, 91120 Palaiseau, France

[d] ENS de Lyon, CNRS, Laboratoire de Chimie, 69342 Lyon, France

[e] University of Bordeaux, CNRS, CRPP, UMR 5031, F-33600 Pessac, France

[f] Institute of Energy Technologies, Fundamental Electrochemistry (IET-1), Forschungszentrum, Jülich, 52425, Germany

[g] University of Bordeaux, CNRS, Bordeaux INP, IMS, UMR 5218, Talence, F-33400, France

[h] University of Bordeaux, CNRS, Bordeaux INP, EPOC, UMR 5805, F-33600 Pessac, France

[i] School of Energy Science and Engineering, Vidyasirimedhi Institute of Science and Technology (VISTEC), Rayong 21210, Thailand

*Corresponding authors: gabriel.loget@cnrs.fr and pichaya.p@vistec.ac.th

Table of contents

		Page
Section 1	Silicon MIS photoanodes prepared by Ni electrodeposition (with Figures S1-S3 and Table S1)	S3
Section 2	Elemental analysis (with Figures S4-S6)	S6
Section 3	Evolution of onset potential and photovoltage (with Figure S7)	S9
Section 4	Mott-Schottky analysis (with Figures S8-S11 and Tables S2-S4)	S10
Section 5	Effects of Fe impurities on the stability of photoanodes (with Figure S12)	S15
Section 6	Analysis of the effects of partial shading on photovoltage (with Figure S13)	S16
Section 7	Spatial mapping of work function on Ni NPs via Kelvin probe force microscopy (KPFM) (with Figure S14)	S18
Section 8	Tung's model and pinch-off effect (with Figures S15-S17)	S20
Section 9	Computational study on NiOOH work function (with Figures S18-S20 and Table S5)	S23
Section 10	Effects of Fe on Co NPs modified n-Si photoanode (with Figure S21 and Table S6)	S29
Section 11	Fe leaching experiment (with Figures S22-S24)	S30
Section 12	Supplementary Tables S7-S11	S32
Section 13	Supplementary Figures S25-S32	S34
Section 14	References	S39

Section 1 - Silicon MIS photoanodes prepared by Ni electrodeposition

n-Si/SiO_x/NiNPs photoanodes, prepared by electrodeposition of randomly dispersed Ni NPs on n-Si, have shown unexpectedly high PEC performance and stability toward water oxidation despite their low Ni coverage.[1,2] With our Ni electrodeposition method, imposing the final consumed electrodeposition charge density (ECD) allows precise control of the Ni loading over the Si surface, and, herein, samples are designated as NiNPs-X, where X is the ECD value: 5, 50, or 200 mC cm^{-2}. Because potentiostatic Ni electrodeposition follows a nucleation-growth mechanism, higher ECD led to larger NPs and higher surface coverage (**Figure S1** and **S2**).

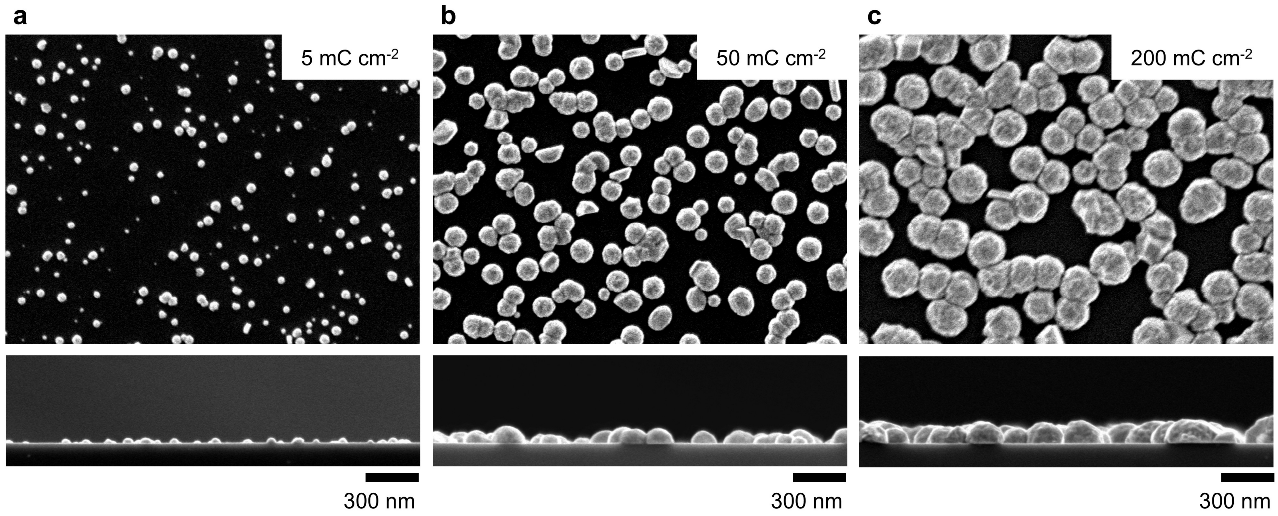

Figure S1. Plan-view (top) and cross-sectional (bottom) SEM images of n-Si/SiO_x/NiNPs deposited with an electrodeposition charge density (ECD) of (a) 5, (b) 50, and (c) 200 mC cm^{-2}.

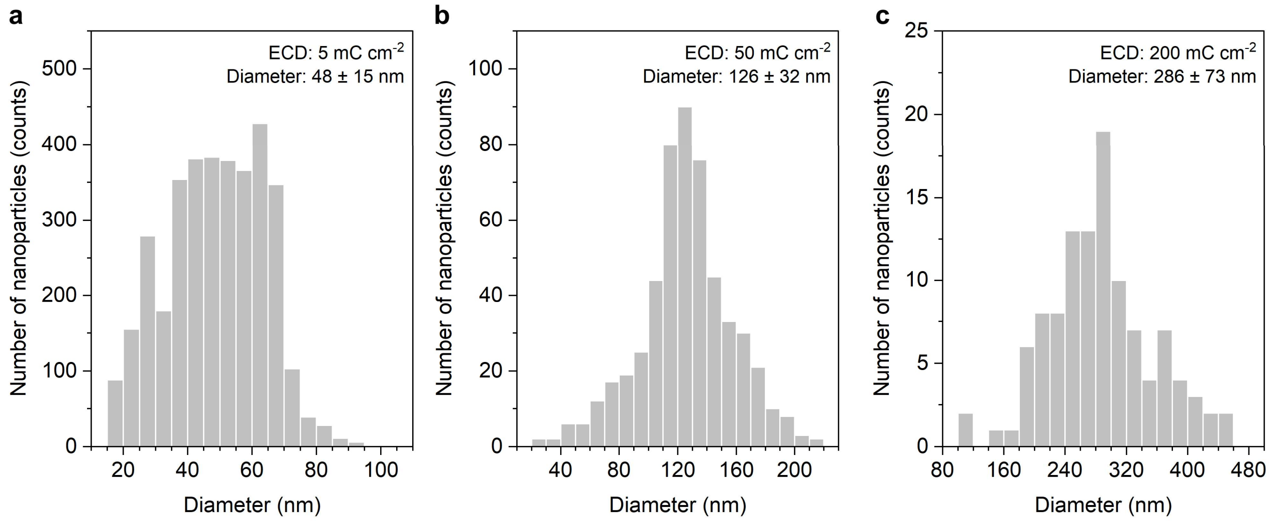

Figure S2. Size distributions of Ni NPs on n-Si electrodeposited with an ECD of (a) 5, (b) 50, and (c) 200 mC cm^{-2}.

Well in line with recent literature reports,[1,3,4] **Figure S3a** shows that our photoanodes with an optimal Ni NPs loading, NiNPs-5, exhibited an onset potential (E_{on}) for OER of ~1.1 V and a current density (j) of 5-10 mA cm^{-2} at 1.23 V. Note that all potentials are reported versus the reversible H_2 electrode (RHE). The PEC performance dropped with increasing ECD: larger E_{on} and lower j. In addition to photoactive n-Si, employing degenerate non-photoactive p$^+$-Si as a substrate (electrode structure: p$^+$-Si/SiO$_x$/NiNPs) allowed us to study the "dark" electrocatalytic performance of Ni NPs, excluding any light-induced effects.[5–8] In contrast with the large variation in PEC performance on n-Si as mentioned above, the electrocatalytic activity of Ni NPs on p$^+$-Si for OER (**Figure S3b**) was relatively similar for the three employed ECD values, i.e., with an E_{on} value of 1.55 V. In this case, the small variations in j are attributed to the differences in Ni active area.

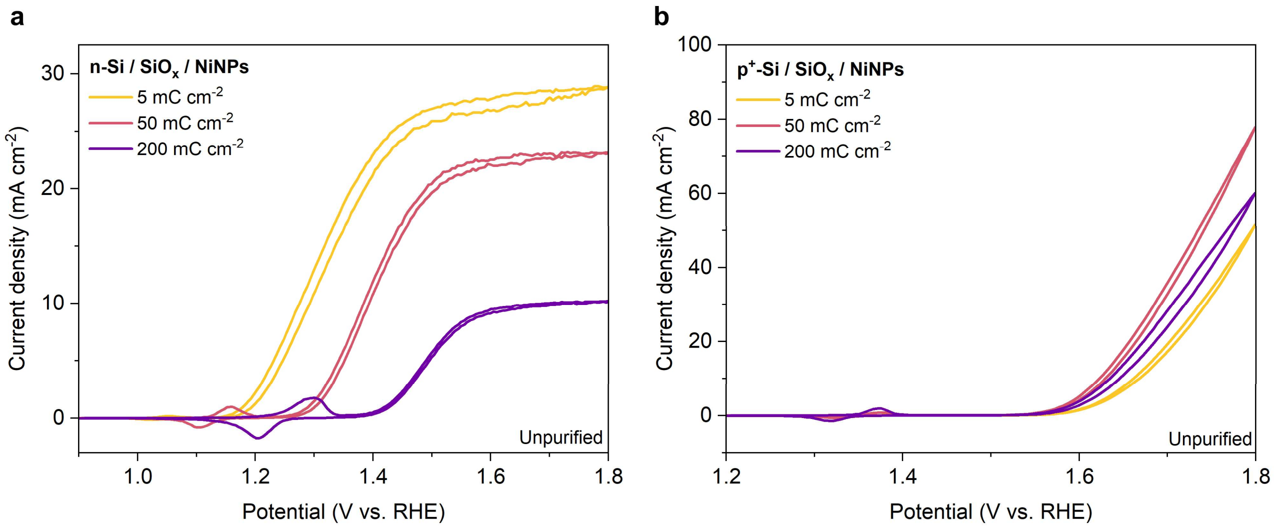

Figure S3. Cyclic voltammograms of (a) n-Si/SiO$_x$/NiNPs under 1-sun illumination and (b) p$^+$-Si/SiO$_x$/NiNPs in the dark. Ni NPs were electrodeposited with charge densities of 5, 50, or 200 mC cm^{-2} in 1 M KOH (unpurified).

The photovoltage (V_{ph}) generated by n-Si/SiO$_x$/NiNPs photoanodes was calculated using the E_{on} values measured from their non-photoactive p$^+$-Si/SiO$_x$/NiNPs counterparts, i.e., V_{ph} = E_{on} (EC, p$^+$-Si) – E_{on} (PEC, n-Si), for each sample condition. As shown in **Table S1**, V_{ph} increased with the decreasing Ni NP loading (lower ECD), as expected from previous reports.[1,4] According to numerous investigations,[4,9–11] this phenomenon can be explained by the pinch-off effect (the depletion region of the high-barrier height overlaps and dominates the lower-barrier height region), which occurs only if the diameter of the metal NPs is smaller than the depletion region of the high-barrier area surrounding them.[4,12] It is generally acknowledged that not only does the size of the NPs matter but also the formation of the oxidized species layer (particularly Ni(OH)$_2$/NiOOH) on the NPs.[1–4,6,13,14] The latter leads to a more intense band bending and a higher effective barrier height due to the higher work function of the NiOOH shell generated during photoanode operation.[4,15]

Table S1. Onset potential (E_{on}) of p^+-Si/SiO_x/NiNPs (EC, in the dark) and n-Si/SiO_x/NiNPs (PEC, under 1-sun illumination) systems, calculated photovoltage (V_{ph}), and saturation photocurrent density (J_{ph}) for Ni NPs deposited from different electrodeposition charge density (ECD). E_{on} was determined at a current density (j) of 0.5 mA cm^{-2} for ECD = 5 and 50 mC cm^{-2} and at j = 2 mA cm^{-2} for ECD = 200 mC cm^{-2}.

ECD / mC cm^{-2}	E_{on} (EC) / V vs. RHE	E_{on} (PEC) / V vs. RHE	V_{ph} / mV	J_{ph} / mA cm^{-2}
5	1.572	1.160	412	29
50	1.544	1.266	278	22
200	1.550	1.386	164	10

Section 2 - Elemental analysis

In scanning transmission electron microscopy (STEM) energy-dispersive X-ray spectroscopy (EDS) for Fe mapping of cross-sectioned samples, a background noise was observed due to an artifact signal caused by the Pt coating layer (see **Figure 2e** in the main text and **Figure S4d**). Despite this noise, **Figure 2e** shows a clear Fe enrichment on the Ni NPs surface for the photoanode activated in the presence of 1.0 ppm Fe. Conversely, this feature was not detected for the control photoanode activated in the purified electrolyte, shown in **Figure S4d**. This confirms the presence of Fe atoms in the $Ni(OH)_2$/NiOOH shell covering the Ni NPs after activation. We further corroborated the presence of Fe by measuring EDS and X-ray photoelectron spectroscopy (XPS) over a larger area on the top surface of n-Si/SiO_x/NiNPs-200. Two sample conditions were used: (1) as-deposited and (2) activated in the 1 M KOH electrolyte containing 1.0 ppm Fe. EDS revealed the presence of Fe only in the activated sample (**Figure 2f** and **Figure S5**), which was further confirmed by XPS. In XPS (with Al Kα source) Fe $2p_{3/2}$ and Ni Auger peaks are located in the same region, nevertheless, Fe $2p_{1/2}$ and Fe 3p peaks were identified at 726.1 eV and 56.8 eV (**Figure 2g** and **2h**), respectively, only in the activated photoanode.[16]

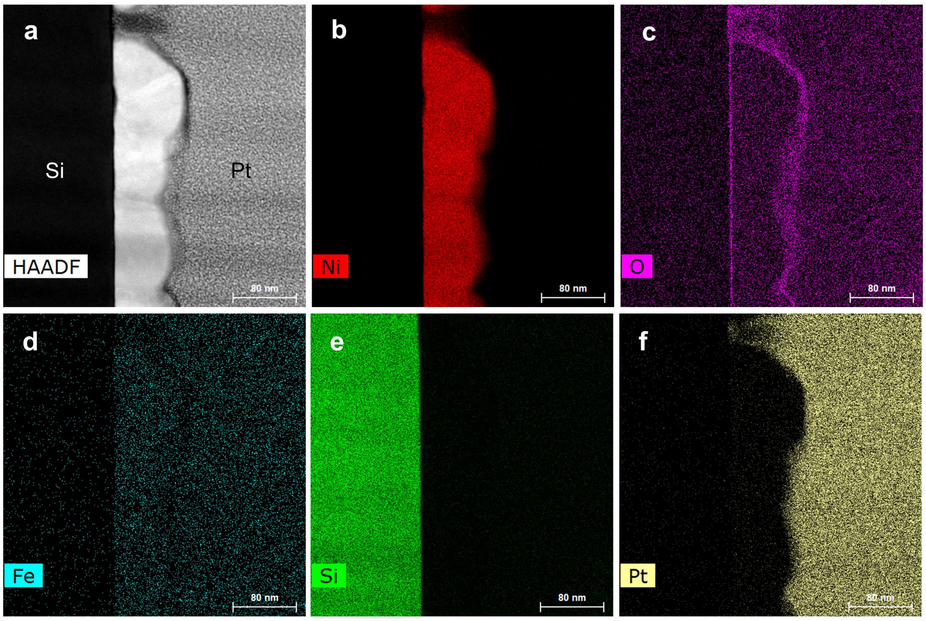

Figure S4. (a) High-angle annular dark-field (HAADF) scanning tunneling electron microscopy (STEM) image and corresponding energy-dispersive X-ray spectroscopy (EDS) elemental mapping of cross-sectioned n-Si/SiO_x/NiNPs-200 activated in the purified 1 M KOH electrolyte: (b) Ni, (c) O, (d) Fe, (e) Si, and (f) Pt.

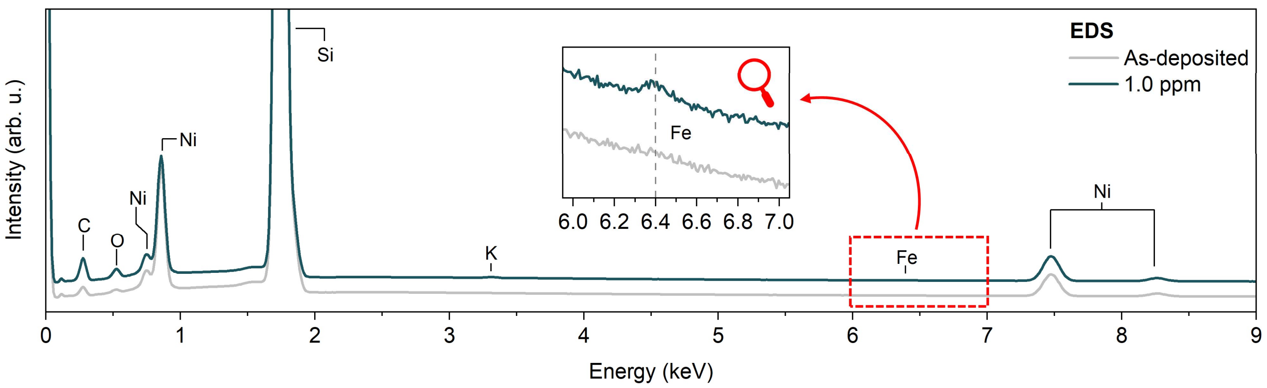

Figure S5. Full-scan EDS spectra of n-Si/SiO_x/NiNPs-200: as-deposited vs activated in the 1 M KOH electrolyte containing 1.0 ppm Fe.

Survey XPS spectra of the as-deposited and activated (in an electrolyte containing 1.0 ppm Fe) photoanode are shown in **Figure S6a**. Ni 2p spectra (**Figure S6b**) show the change of Ni NPs composition and phase after the activation. The deconvolutions at Ni $2p_{3/2}$ were done on both spectra to reveal the phase of Ni species on the photoanode before and after the activation. As shown in **Figure S6c**, the spectrum contained three main peaks located at 852.7 eV, 854.2 eV and 856.6 eV, belonging to Ni metal, NiO, and $Ni(OH)_2$/NiOOH, respectively.[15,17] This result could be easily interpreted as there was a shell of NiO and $Ni(OH)_2$ covering the Ni^0 in the Ni NPs. After the activation of the photoanode, the intensity of $Ni(OH)_2$/NiOOH increased, indicating a larger shell of $Ni(OH)_2$/NiOOH on Ni^0. However, the deconvolution of Ni $2p_{3/2}$ spectra cannot distinguish between the phases of $Ni(OH)_2$ vs NiOOH because the peaks of these two species are found at similar binding energies.[17]

To investigate whether the sample contained one or both species, we did the deconvolution of the O 1s spectra (**Figure S6e-g**). The peaks appearing around 532.7 eV - 532.9 eV could belong to oxygen from the adventitious carbon, SiO_x, or adsorbed oxygen.[18,19] The as-deposited photoanode had relatively lower amounts of Ni oxide species when compared to the activated photoanode. The peaks observed at 529.7 eV and 531.6 eV on the as-deposited photoanode could be assigned to the oxygen of NiO and $Ni(OH)_2$, respectively.[15,18] From this result, we can rule out the presence of NiOOH on the as-deposited sample. In addition to the higher intensity of the O 1s from $Ni(OH)_2$, as expected, we also found an additional peak at 530.6 eV belonging to the oxygen in the hydroxyl group from NiOOH.[20] However, the peak of oxygen not bonded to a hydrogen atom is known to be located at the same binding energy of that of oxygen in NiO.[18] In **Figure S6g**, it can be seen that the intensity of the O 1s peak at 529.5 eV is higher when compared to that of the as-deposited sample (**Figure S6f**). If this peak originated from NiO, its intensity should be similar for both samples, due to the relatively comparable peak intensity and area of NiO extracted from the Ni 2p fits. As seen from **Figure S6c** and **d**, the values of the NiO peak areas were 22000 and 18000 for as-deposited and activated samples, respectively, indicating similar amounts of NiO. With all these results, we conclude that the increased intensity observed with the activated sample on the O 1s peak at 529.5 eV originated from oxygen bonded to Ni in NiOOH.

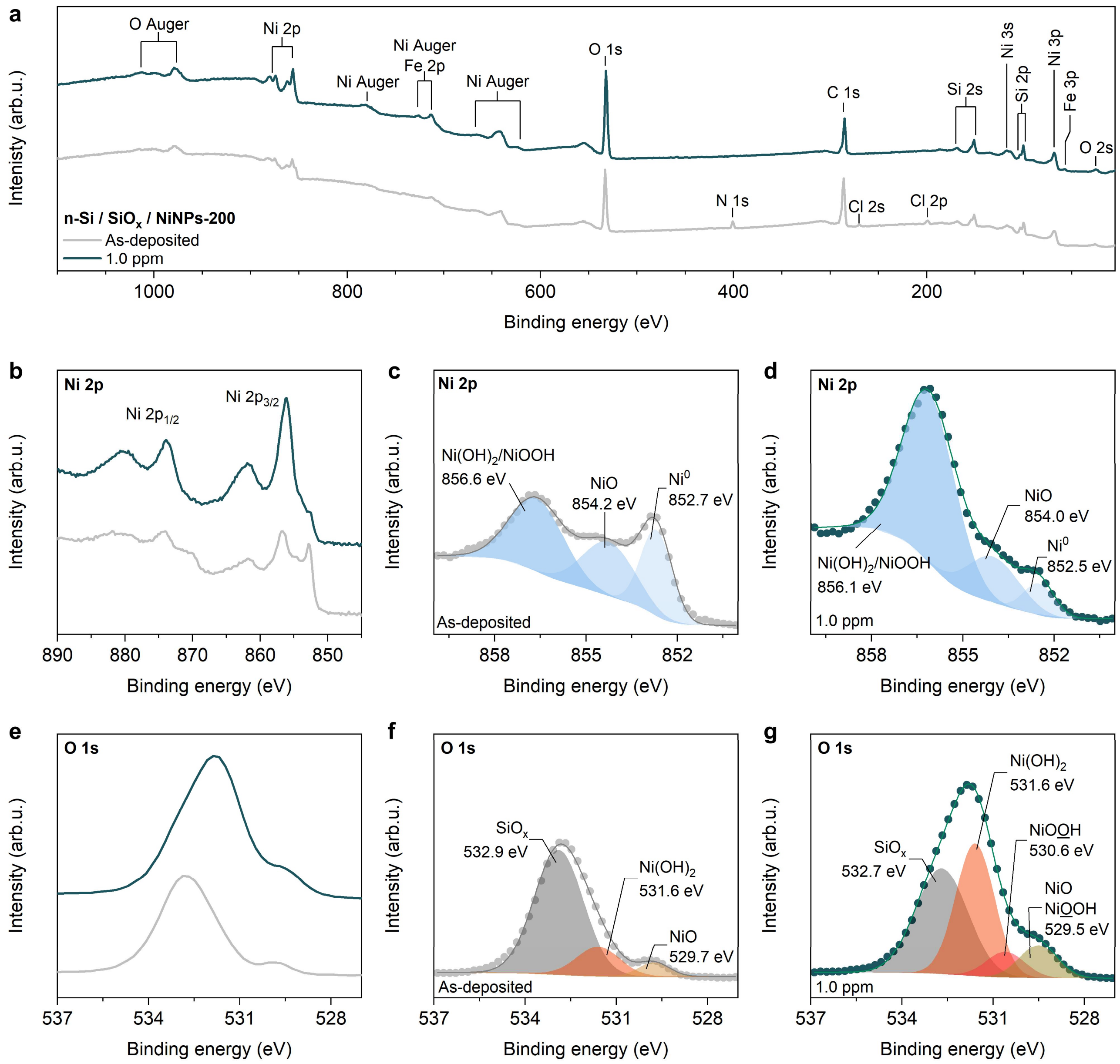

Figure S6. XPS spectra of as-deposited and activated (1.0 ppm Fe) n-Si/SiO_x/NiNPs-200: (a) Survey spectra, (b)-(d) high-resolution Ni 2p spectra, and (e)-(g) high-resolution O 1s spectra. Note that the intensity scale is the same for (c) vs (d) and (f) vs (g).

Section 3 - Evolution of onset potential and photovoltage

NiNPs-5:

In the purified electrolyte (**Figure S7a**), E_{on} increased in both EC and PEC systems while there was a slight change in V_{ph} (ΔV_{ph} = 10 mV). In the 0.1 ppm electrolyte (**Figure S7b**), V_{ph} was considerably higher compared to the purified electrolyte (~434 mV vs ~388 mV). Interestingly, V_{ph} started off at a higher value immediately at the early potential scan and remained relatively constant while drastic changes in E_{on} were observed. It appears that Fe incorporation has an immediate impact on the photovoltage even though the optimal electrocatalytic activity has not been reached. In the case of the high Fe concentration of 1.0 ppm (**Figure S7c**), both E_{on} and V_{ph} were steady over the potential scans, indicating the enhanced incorporation of Fe onto the NiNPs without the need of an applied bias.

NiNPs-200:

In the purified electrolyte, a relatively similar trend was observed when compared to NiNPs-5 (**Figure S7d**). With 0.1 and 1.0 ppm electrolytes (**Figure S7e-f**), V_{ph} remained relatively constant while E_{on} evolved with the potential scans. It is worth noting that the high concentration of Fe at 1.0 ppm has a stronger effect on the electrocatalytic evolution of NiNPs-5 than that of NiNPs-200, for which we attribute to the higher Fe to Ni ratio of the former.

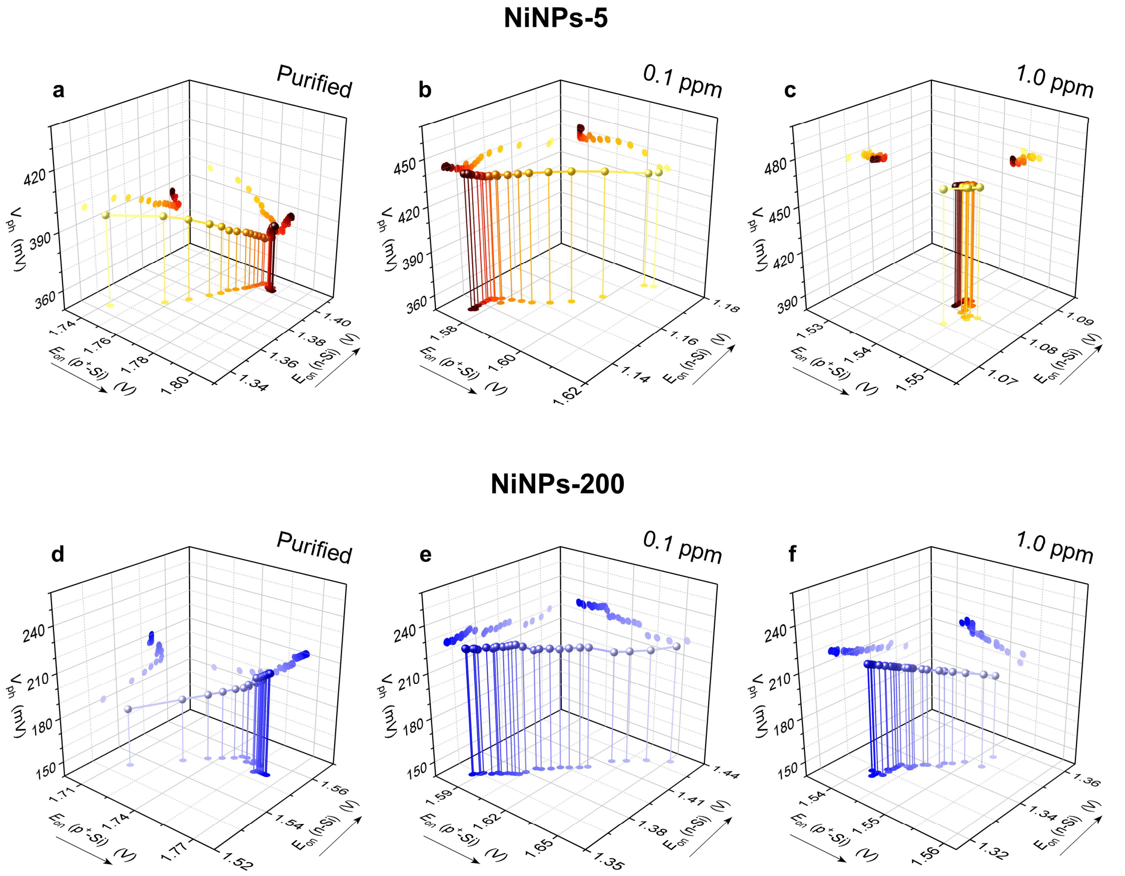

Figure S7. Evolution of the onset potential (E_{on} in V vs. RHE) obtained from p^+-Si and n-Si and photovoltage (V_{ph}) of (a)-(c) NiNPs-5 and (d)-(f) NiNPs-200 in different electrolytes. The colors start from the lighter shades of the early scans (from the 4th scan) to darker shades of the later scans (to the 100th scan) with a step of 4 scans for each point.

Section 4 - Mott-Schottky analysis

To obtain the capacitance of the space charge layer (C_{sc}), we used the equivalent circuits shown in **Figure S8** to fit the impedance spectra. In all circuits, R_1 is the series resistance originating from the electrical contact in the device and wire and C_2 is considered to consist of C_{sc} and the capacitance of SiO_x (C_{ox}) in series.[21,22] Thus, C_{sc} is calculated as shown in **Eq. (1)**.

$$C_{sc}= \frac{C_2 C_{ox}}{C_{ox}-C_2} \tag{1}$$

C_{ox} can be calculated as follows:[21,22]

$$C_{ox}= \frac{\varepsilon_0 \varepsilon_{ox}}{t_{ox}} \tag{2}$$

where ε_0 is the vacuum permittivity (8.85×10^{-14} F cm^{-1}), ε_{ox} is the relative permittivity of SiO_2 (3.9), and t_{ox} is the thickness of the SiO_x layer. The latter was measured to be 2 nm from the STEM image (**Figure 2b**, main text). The calculated C_{ox} is in the order of μF cm^{-2} while C_{sc} obtained from the fitting is in the order of nF cm^{-2}, in good agreement with the expected range for the space charge layer of Si. Thus, C_{sc} calculated using **Eq. (1)** is approximately equal to C_2. In Model 2, C_3 is considered to be the surface state capacitance.[23,24] On the other hand, C_3 in Model 3 can be attributed to other charge trapping processes.[24] As expected, C_3 in Models 2 and 3 are in the same order of magnitude or slightly higher compared to C_{SC} and relatively constant over the fitting range of the Mott-Schottky plot.

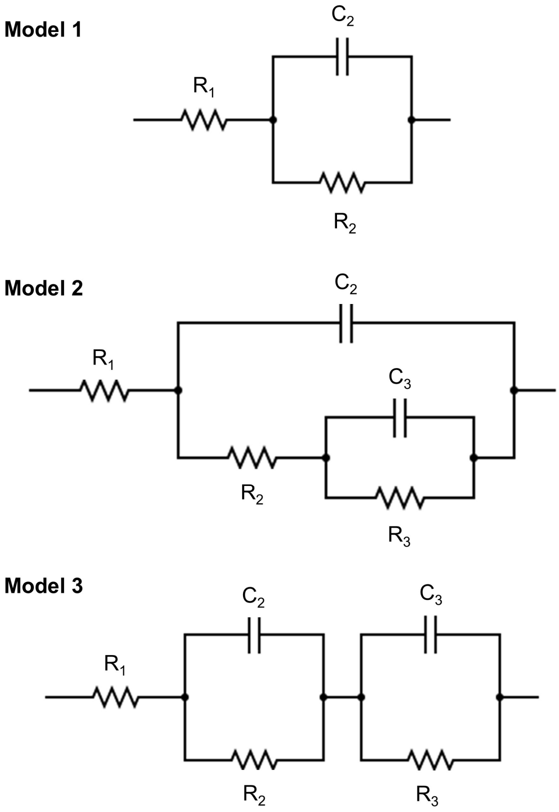

Figure S8. Equivalent circuit models used in the analysis and fitting of Nyquist plots.

In **Figure S9a-c**, Nyquist plots of each sample used for the fitting are shown. C_{sc} was obtained for each bias voltage and the Mott-Schottky curves were plotted as $1/C_{sc}^2$ versus the bias voltage. The current density-voltage curve of the solid-state samples activated in different electrolytes exhibited the expected diode properties, with a high *j* in the forward regime and a low *j* in the reverse regime, as shown in **Figure S9d**. After fitting the linear region of the plots (**Figure S9e-f**), the *x*-intercepts and slopes were obtained. The Mott-Schottky relationship can be written as:

$$C_{sc}^{-2} = \frac{2}{\varepsilon_0 \varepsilon_{Si} q N_D}\left(\text{Bias} - E_{fb} - \frac{kT}{q}\right) \tag{3}$$

Where ε_{Si} is the relative permittivity of Si (11.7), q is the elementary charge (1.6×10^{-19} C), N_D is the donor density in the semiconductor (Si), E_{fb} is the flat-band potential, k is Boltzmann's constant (1.38×10^{-23} m^2 kg s^{-2} K^{-1}), and T is the temperature (in K). From this relationship, E_{fb} is equal to the *x*-intercept minus kT/q, and N_D can be calculated from the slope with **Eq. (4)**:

$$N_D = \frac{2}{\varepsilon_0 \varepsilon_{Si} q \text{ (slope)}} \tag{4}$$

For all models (results of Model 1 shown in **Figure 3f**, Model 2 in **Figure S9e**, and Model 3 in **Figure S9f**), we found that the absolute value of E_{fb} increased with the amount of Fe. This indicates the systematic change in the junction energetics depending on the presence of Fe, as shown in **Table S2**. The calculated N_D gave the resistivity of the n-Si wafer in the range of 1-3 Ω cm, which complies with the manufacturer's specifications. The effective barrier height (ϕ_{eff}) can be calculated using **Eq. (5)**:

$$\phi_{eff} = -qE_{fb} + \phi_n \tag{5}$$

ϕ_n is calculated with the following equation:

$$\phi_n = kT\ln(N_c/N_D) \tag{6}$$

where N_c is the effective density of states in the conduction band, which is approximately 3×10^{19} cm^{-3} for Si at room temperature.[25]

Table S2. Parameters extracted from Mott-Schottky analysis of n-Si/SiO_x/NiNPs-5 activated in different electrolytes.

Model	Fe concentration ppm	*x*-Intercept V	Slope F^{-2} cm^4 V^{-1}	E_{fb} V	N_D cm^{-3}	ϕ_n eV	ϕ_{eff} eV
1	Purified	-0.340	1.14E+16	-0.364	1.46E+15	0.255	0.62
	0.1	-0.621	1.20E+16	-0.645	1.39E+15	0.256	0.90
	1.0	-0.882	1.16E+16	-0.906	1.43E+15	0.257	1.16
2	Purified	-0.513	1.50E+16	-0.537	1.11E+15	0.262	0.80
	0.1	-0.649	1.46E+16	-0.673	1.14E+15	0.261	0.93
	1.0	-1.130	1.50E+16	-1.154	1.11E+15	0.262	1.42
3	Purified	-0.296	1.25E+16	-0.320	1.33E+15	0.257	0.58
	0.1	-0.400	1.30E+16	-0.424	1.28E+15	0.258	0.68
	1.0	-0.779	1.28E+16	-0.803	1.30E+15	0.258	1.06

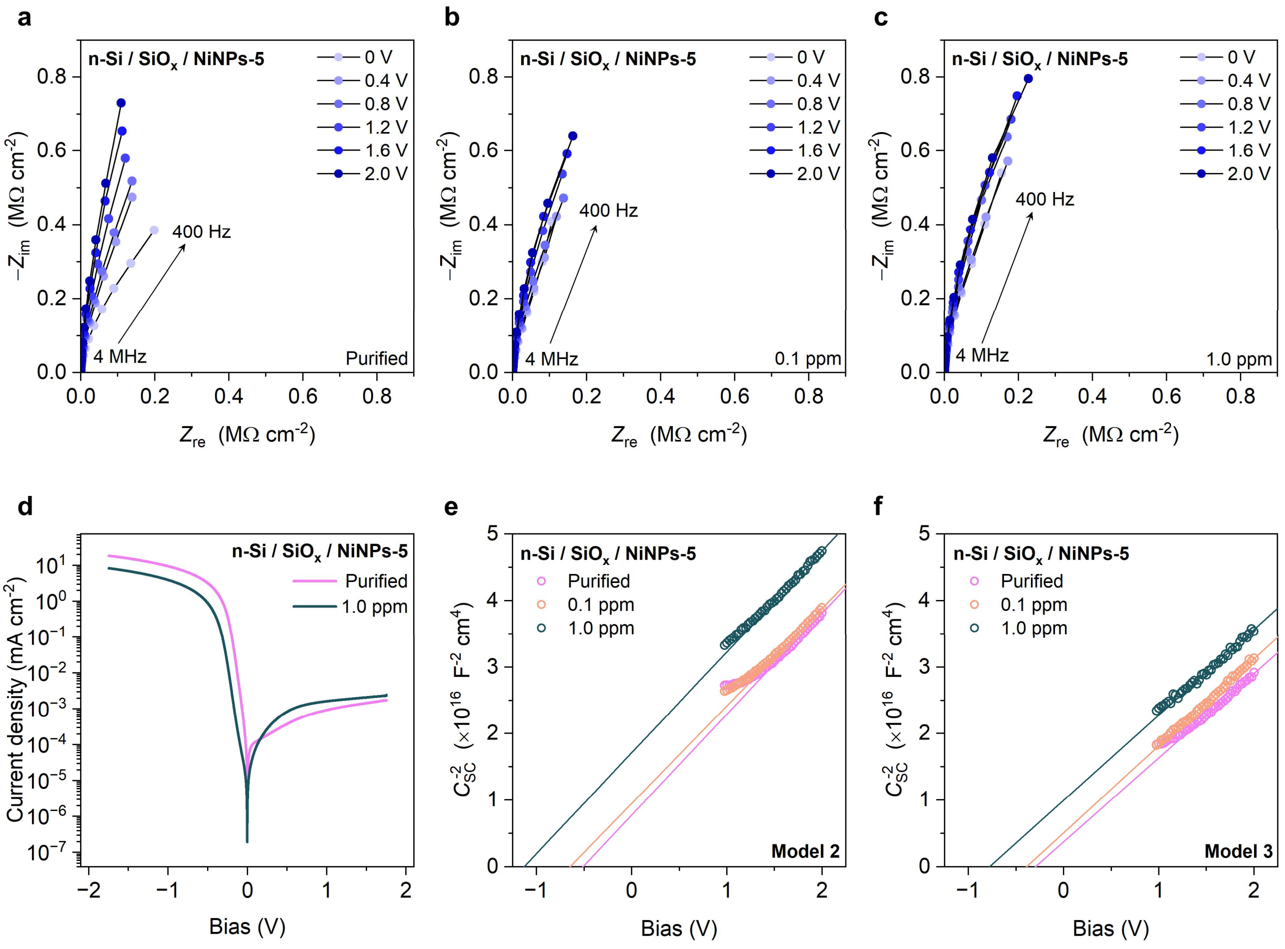

Figure S9. Nyquist plots recorded with n-Si/SiO_x/NiNPs-5 activated in different 1 M KOH electrolytes: (a) purified, (b) 0.1 ppm Fe, and (c) 1.0 ppm Fe. (d) Current density–voltage characteristics (solid-state) of n-Si/SiO_x/NiNPs-5 activated in purified and 1.0 ppm Fe electrolytes. Mott-Schottky plots of n-Si/SiO_x/NiNPs-5 activated in different electrolytes with C_{sc} obtained from (e) Model 2 and (f) Model 3. (Analysis based on Model 1 is included in the main text, **Figure 3f**).

We further investigated the effects of Fe incorporation on Si photoanode modified with a homogeneous sputtered Ni thin film (thickness of 20 nm), denoted as n-Si/SiO_x/NiFilm. The photoanodes were activated in purified and 1.0 ppm Fe electrolytes. As shown in **Figure S10a**, improved PEC performance was recorded when the photoanode was activated in the solution containing higher Fe concentration. Then, the current-voltage curve and impedance spectra were recorded (**Figure S10b-d**). Mott-Schottky plots of the photoanodes are shown in **Figure S10e**. Note that, in this case only the Model 3 equivalent circuit (**Figure S8**) fit satisfactorily the Nyquist data. All parameters obtained and calculated from the Mott-Schottky analysis of n-Si/SiO_x/NiFilm are summarized in **Table S3**. In contrast to the NiNPs-5 sample, the calculated effective barrier height of NiFilm barely changed with the higher Fe concentration despite the large improvement in the PEC performance. Thus, it was concluded that Fe content had a much weaker effect on this junction behavior, despite a considerable change in the PEC performance.

Similar measurements were also carried out on n-Si/SiOx/NiNPs-200, as shown in **Figure S11,** and the calculated parameters are included in **Table S4**. Similar to the Ni Film sample, only Model 3 gave a reasonable fit to the Nyquist data.

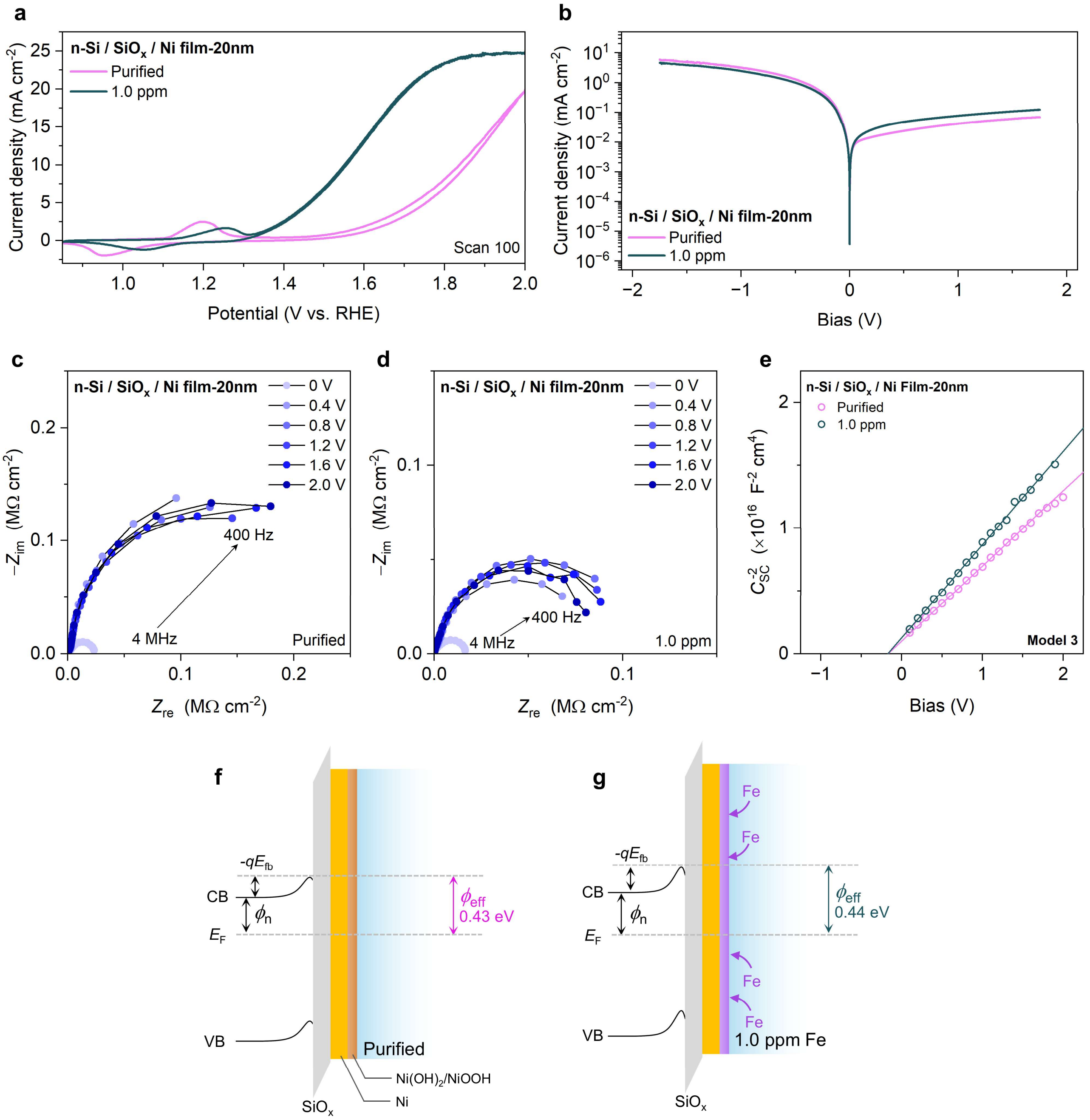

Figure S10. (a) Cyclic voltammograms recorded in 1 M KOH under 1-sun illumination and (b) current density–voltage characteristics (solid-state) of n-Si/SiOx/NiFilm-20nm activated in purified and 1.0 ppm Fe electrolytes. Nyquist plots of n-Si/SiOx/NiFilm-20nm activated in (c) purified and (d) 1.0 ppm Fe electrolytes. (e) Corresponding Mott-Schottky plots of n-Si/SiOx/NiFilm-20nm activated in two different electrolytes. Schematic energy band diagrams of n-Si/SiOx/NiFilm-20nm activated in the (f) purified and (g) 1.0 ppm Fe electrolytes displaying the barrier height (ϕ_{eff}) of each system.

Table S3. Parameters extracted from Mott-Schottky analysis of n-Si/SiO_x/NiFilm-20nm activated in purified and 1.0 ppm Fe electrolytes.

Model	Fe concentration	X intercept V	Slope F^{-2} cm^4 V^{-1}	E_{fb} V	N_D cm^{-3}	ϕ_n eV	ϕ_{eff} eV
3	Purified	-0.167	6.00E+15	-0.191	2.77E+15	0.24	0.43
	1.0 ppm	-0.170	7.44E+15	-0.194	2.24E+15	0.24	0.44

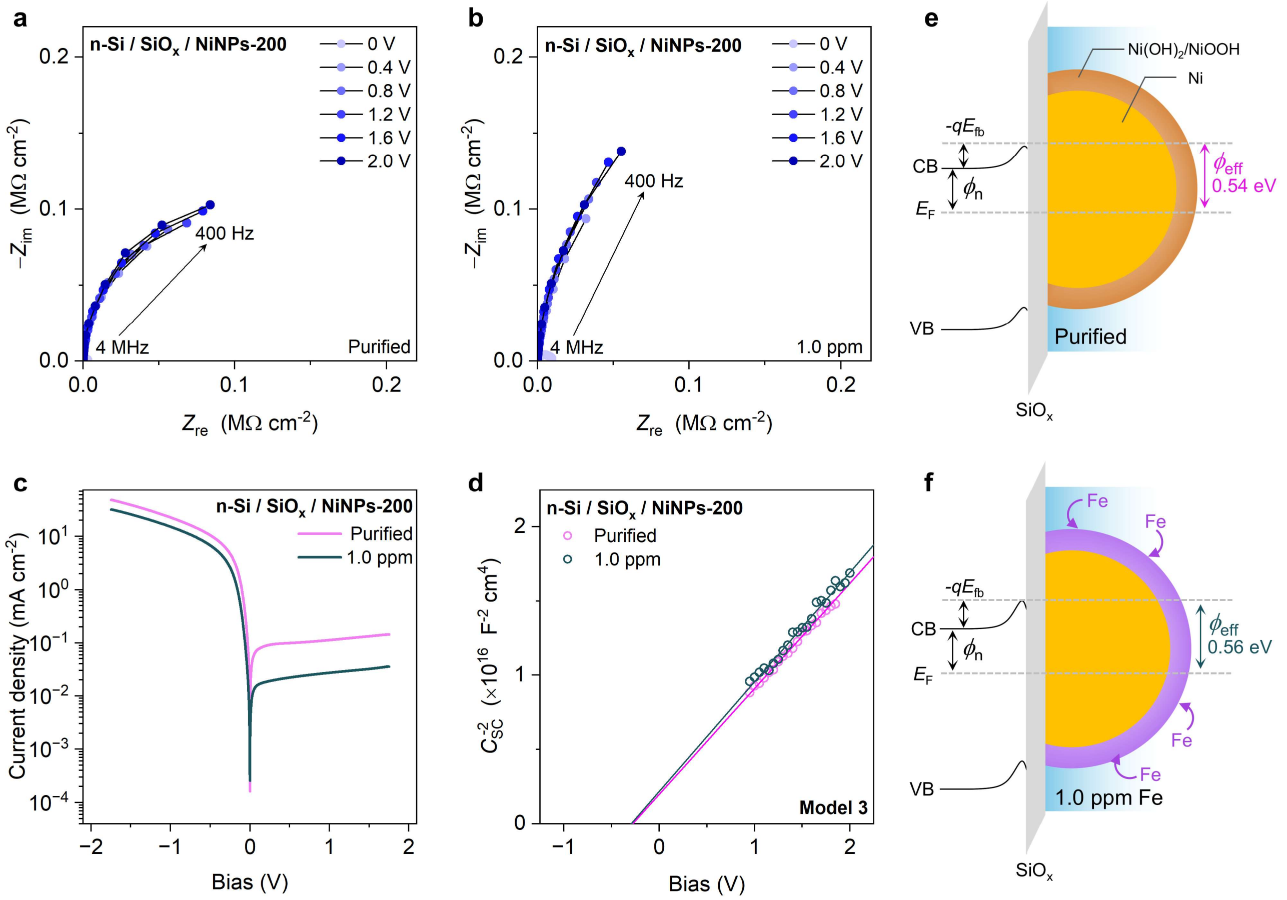

Figure S11. Nyquist plots of n-Si/SiO_x/NiNPs-200 activated in (a) purified and (b) 1.0 ppm Fe. (c) Current density–voltage characteristics (solid-state) of n-Si/SiO_x/NiNPs-200 activated in purified and 1.0 ppm Fe electrolytes. (d) Mott-Schottky plots of n-Si/SiO_x/NiNPs-200 activated in different electrolytes. Schematic energy band diagrams of n-Si/SiO_x/NiNPs-200 activated in the (e) purified and (f) 1.0 ppm Fe electrolytes displaying the barrier height (ϕ_{eff}) of each system.

Table S4. Parameters extracted from Mott-Schottky analysis of n-Si/SiO_x/NiNPs-200 activated in purified and 1.0 ppm Fe electrolytes.

Model	Fe concentration	X intercept V	Slope F^{-2} cm^4 V^{-1}	E_{fb} V	N_D cm^{-3}	ϕ_n eV	ϕ_{eff} eV
3	Purified	-0.278	7.12E+15	-0.302	2.34E+15	0.24	0.54
	1.0 ppm	-0.294	7.38E+15	-0.318	2.25E+15	0.24	0.56

Section 5 - Effects of Fe impurities on the stability of photoanodes

The long-term stability of Ni NPs-modified Si photoanode (n-Si/SiO_x/NiNPs-5) in the presence/absence of Fe impurities is shown in **Figure S12a**. From these curves, it was found that Fe did not have significant effects on the stability of these photoanodes. Interestingly, when a thin Ni film (5 nm) was used as the coating layer, the photoanode drastically degraded in the purified electrolyte (i.e. without Fe impurities), as indicated by a drastic decrease in PEC performance after only 60 CV scans (**Figure S12b**). On the contrary, in the Fe-containing electrolyte, after 100 CV scans, the photoanode could still perform efficiently (**Figure S12c**). We infer that the stability of the photoanode with Ni NPs might depend predominantly on the stability of n-Si/SiO_x and SiO_x/Ni interfaces and not on the Ni NPs co-catalyst. This is based on the fact that most of the Si surface is exposed to electrolyte and is prone to degradation in the highly alkaline environment. The fast degradation seen in the case of thin Ni film was not observed on Ni NPs presumably because the NPs height (>25 nm) was at least 5 times larger than the film (5 nm). For photoanodes covered by the Ni thin film, the stability seems to rely heavily on the co-catalyst properties. The fast degradation of Ni film in the absence of Fe impurities is thought to originate from both phase/structural changes according to other studies in the literature. For instance, Mattinen *et al.* have shown that $Ni(OH)_2$/NiOOH of various Ni-based electrocatalysts undergo different pathways of the phase change regarding the presence of Fe impurities.[26] This might induce further changes in the film structure differently during the operation. In addition, Huang *et al.* observed that the stability of anodized Ni foams is also higher in Fe-containing electrolytes.[27]

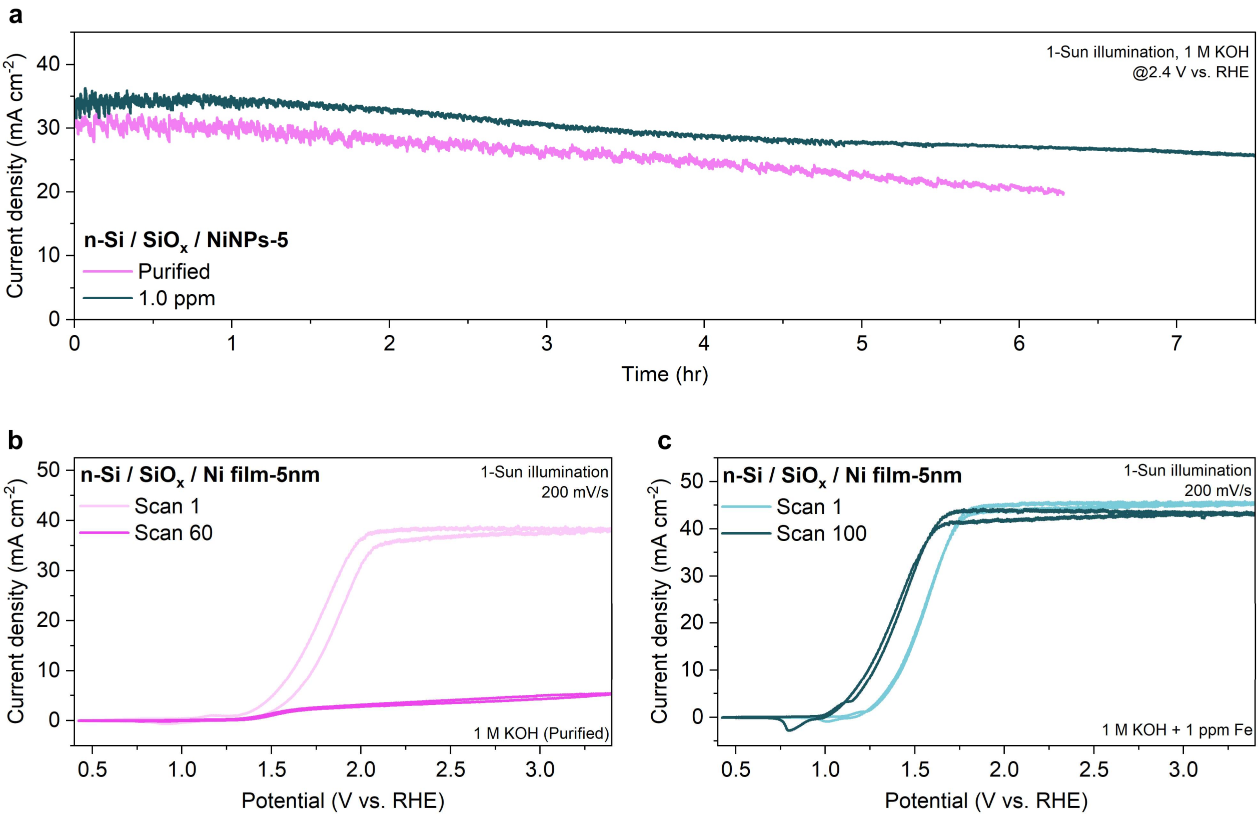

Figure S12. (a) Long-term stability test of n-Si/SiO_x/Ni NPs-5 in purified and Fe-containing 1 M KOH electrolyte under 1-sun illumination. CVs recorded on n-Si/SiO_x/Ni film-5nm in (b) purified and (c) Fe-containing electrolyte under 1-sun illumination.

Section 6 - Analysis of the effects of partial shading on photovoltage

To investigate the impact of the partial shading on the system photovoltage, we refer to the PEC analog of the diode equation:[28]

$$J = J_s\left[\exp\left(\frac{qV}{nkT}\right) - 1\right] - J_{ph} \tag{7}$$

Where J is the total current density through the semiconductor/electrocatalyst interface, J_s is the dark saturation current density, J_{ph} is the saturation photocurrent density related to the generation of the carriers by light illumination, n is the ideality factor, and V is the applied bias.

At $J = 0$, the photovoltage, V_{ph}, is obtained from:

$$V_{ph} = \frac{nkT}{q}\ln\left(\frac{J_{ph}}{J_s} + 1\right) \tag{8}$$

According to the literature, J_s relates strongly to the electron recombination current and can be written with the correction term for tunneling in the case of an MIS junction as follows:[9]

$$J_s = A^{*}T^2 T_t \exp\left(-\frac{q\phi_b}{kT}\right) \tag{9}$$

A^* is the Richard's constant, ϕ_b is the barrier height, and T_t is the tunneling probability of the electron through the barrier, which subsequently recombines in the metal. For a direct Si/Ni contact, T_t equals to 1.

Herein, we investigated V_{ph} as a function of J_{ph} which would indicate how the photovoltage evolves with the charge generation. ϕ_b was set to 0.8 eV based on the values measured from our photoanodes. n was chosen to be 1.4 based on the measurements done by Laskowski *et al.*[4] For simplicity, we first neglected the T_t term (set to 1) as a variation in this term would have the same effects regardless of the lighting/shading conditions. The photovoltage profile as a function of J_{ph} is shown in **Figure S13a**.

To select a suitable range of the saturation photocurrent density for our analysis, we used directly the current density in the saturated regime measured on the photoanode since the dark current was negligible, as demonstrated in **Figure S13b**. This photocurrent density was the direct indication of the amount of photogenerated charge carriers, and the values of J_{ph} of *n*-Si/SiO_x/NiNPs-x are summarized in **Table S1**. Focusing on the change of J_{ph} between 10 and 30 mA cm^{-2} (which corresponds to the photocurrent plateaus observed in **Figure S3a**), the photovoltage difference attributed to partial shading (i.e., reduced J_{ph}) was only around ~45 mV. This change is considered to be much smaller than the value we observed between n-Si/SiO_x/NiNPs-5 and n-Si/SiO_x/NiNPs-200 which was around 200-250 mV.

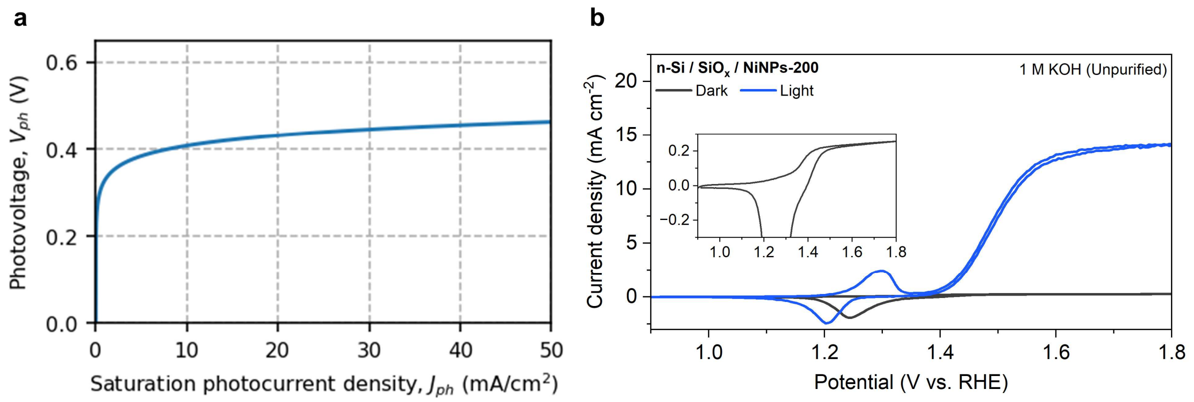

Figure S13. (a) Photovoltage as a function of saturation photocurrent density calculated based on the diode equation. (b) CVs recorded on n-Si/SiO_x/NiNPs-200 in unpurified 1 M KOH under dark conditions and 1-sun illumination. The scan rate was 100 mV s^{-1}.

Section 7 - Spatial mapping of work function on Ni NPs via Kelvin probe force microscopy (KPFM)

We attempted to spatially probe the junction properties of the interface on photoactive n-Si/SiO_x/NiNPs following the work done by Lechaptois *et al.*, in which the Schottky barrier of Au NPs on Si substrate was visualized through KPFM.[29] Unfortunately, we were not able to obtain a reliable junction analysis on the semiconducting n-Si substrate. This issue appeared to be related to the nature of our samples and the limited resolution of available probes. Through further geometrical optimization of the samples and more in-depth studies, the visualization of the barrier properties could be possible in future work.

Nevertheless, we were able to perform KPFM on Ni NPs deposited on the conductive p^+-Si substrates . **Figure S14** shows AFM topography images and their corresponding work function maps for Ni NPs-100 on degenerate, non-photoactive p^+-Si substrates. In **Figure S14b**, a very small difference in the measured work function can be observed between Ni NPs and the Si substrate for all conditions (on the order of ~0.1 eV). We remark that the work function probed on p^+-Si was different for each sample. This was unexpected since the work function of the highly doped p^+-type Si should not be affected by Ni NPs and, indeed, was measured to be around 5.0 eV on a bare surface (data not shown). However, based on the high density of the particles, the KPFM tip was likely disturbed by the surrounding Ni NPs while probing on the Si surface. In **Figure S14c,** the work function maps were rescaled to the same range for all samples to visualize the work function trend of the overall surface. It can be clearly seen that the work function exhibited the same trend as the result obtained with the Kelvin probe. The average work functions were 4.60, 4.82, and 5.07 eV for the as-deposited, activated in the purified electrolyte, and activated in the Fe-containing electrolyte, respectively.

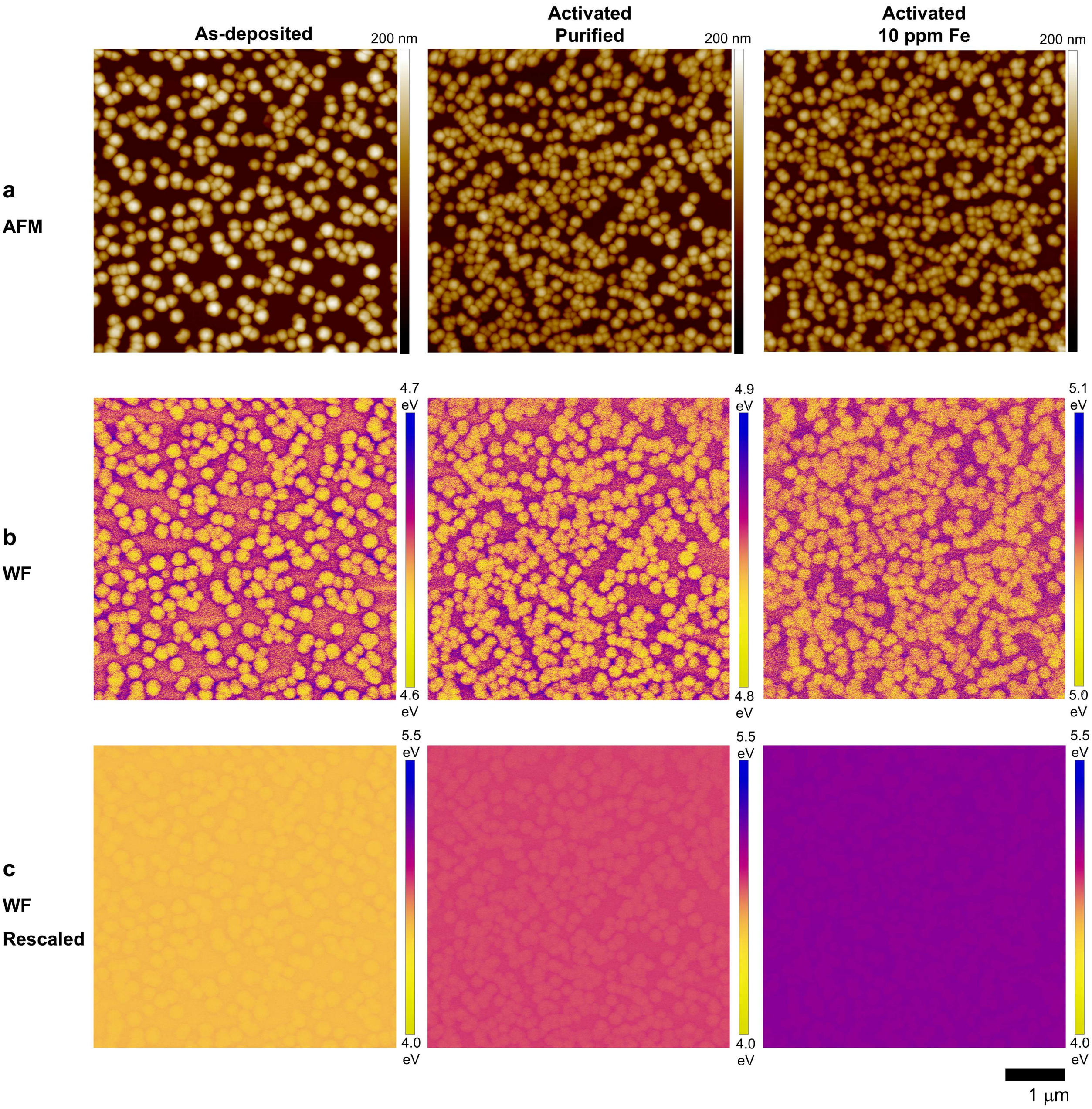

Figure S14. (a) AFM topography images and (b),(c) their corresponding work function (WF) maps of p^+-Si/SiO_x/NiNPs-100 (as-deposited, activated in the purified electrolyte, and activated in the 10 ppm Fe electrolyte.

Section 8 - Tung's model and pinch-off effect

We employed Tung's model as a basis for the junction analysis upon the change in the surrounding environment around a metal patch (i.e., the shell surrounding Ni NPs on Si in our case), as shown in **Figure S15**.[30,31] It should be noted that this analytical model considers the high-barrier region surrounding the metal patch to be semi-infinite and neglects the effect of the SiO_x layer. The model describes the variation of the conduction band minimum, CBM, (with the Fermi level as the reference point) inside the semiconductor. The CBM as a function of the semiconductor depth at the center of the metal patch can be written as follows:

$$V(0,z) = V_{\mathrm{bb}}\left(1 - \frac{z}{W}\right)^2 + V_{\mathrm{n}} + V_{\mathrm{a}} - \Delta\left(1 - \frac{z}{\sqrt{z^2 + R_0^2}}\right) \quad (10)$$

$$V_{\mathrm{bb}} = \phi_{\mathrm{B}}^0 - V_{\mathrm{n}} - V_{\mathrm{a}} \quad (11)$$

$$W = \sqrt{\frac{2\varepsilon_{\mathrm{Si}}\varepsilon_0 V_{\mathrm{bb}}}{qN_{\mathrm{D}}}} \quad (12)$$

where V_{bb} is the band bending in the depletion region within the semiconductor, z is the semiconductor depth, W is the width of the depletion region, V_n is the difference between Fermi level and conduction band in the bulk and calculated to be 0.25 eV at room temperature, V_a is the applied potential, ϕ_{B}^0 is the barrier height for the region surrounding metal patch, Δ is the difference in the barrier height between the metal patch and the surrounding region, and R_0 is the particle/patch radius. N_D, donor density, is chosen to be 1×10^{15} cm^{-3}, which roughly corresponds to the resistivity of the Si wafer used in this work (1-10 Ω·cm).

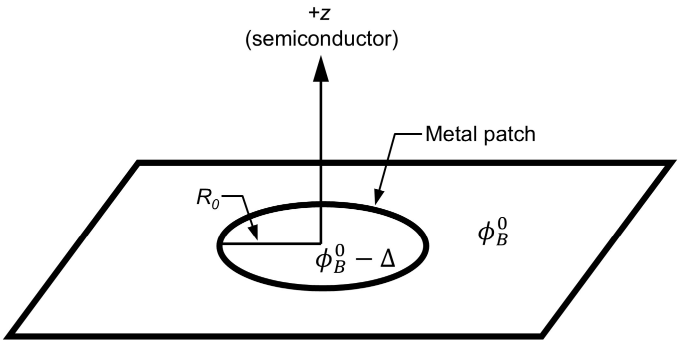

Figure S15. Scheme displaying the geometries and coordinates employed in the calculation. Adapted from R. T. Tung.[30]

As determined by the Mott-Schottky analysis, the barrier height of Ni film on n-Si was around 0.45 eV. We thus assigned this value as the barrier height between Ni patch and Si in this calculation. The work functions (4.82 and 5.07 eV for Ni NPs activated in the purified and Fe-containing electrolytes, respectively) measured by KPFM, WF_{exp}, were used to calculate the barrier height of the surrounding region:

$$\phi_b^0 = WF_{exp} - c \quad (13)$$

χ is electron affinity of Si which is around 4.05 eV (the difference between vacuum level and conduction band minimum).[32] The calculated barrier heights were approximately ~0.8 and ~1.0 eV for the purified and Fe-incorporated cases, respectively. It should be noted that these are estimated values since the presence of thin SiO_x might alter the actual barrier height. In **Figure S16**, at zero bias (equilibrium), the CBM profiles inside Si at the center of Ni NPs having two different sizes are shown with varying barrier heights from 0.7 eV to 1.1 eV of the surrounding area. It can be clearly seen that the influence of the surrounding region is stronger when the particle is smaller, especially near the interface (near z = 0 nm). In the case of small Ni NPs, the band edge is shifted by ~0.4 eV if the barrier height induced by the surrounding region changes from 0.7 eV to 1.1 eV.

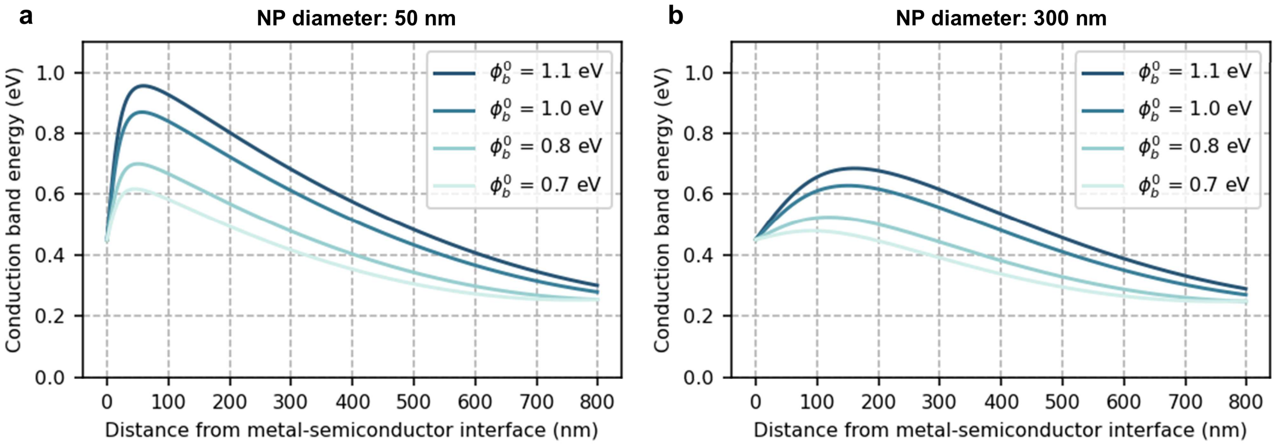

Figure S16. CBM profiles into Si at the center of an Ni particle/patch with varying ϕ_B^0 when the nanoparticle's diameter is (a) 50 nm or (b) 300 nm. The applied bias is set to 0 V.

We also performed the calculations of the spatial distribution of the CBM across the metal patch at varying semiconductor depth using **Eq. (14)**:[31]

$$V(\rho,z) = V_{bb}\left(1 - \frac{z}{W}\right)^2 + V_n + V_a - \frac{V_{bb}\Gamma^3 z W^2}{(\rho^2 + z^2)^{3/2}} \quad (14)$$

$$\Gamma^3 = \frac{\Delta R_0^2}{2V_{bb}W^2} \quad (15)$$

The coordinates used originally in the equation are polar coordinates which can be converted to Cartesian coordinates by replacing ρ^2 with x^2+y^2. Since both x and y directions would give the same results for a circular patch, we thus performed the calculations along the x-axis (y set to 0). These results are plotted in **Figure S17** and demonstrate that the large variation in the CBM leading to the pinch-off effect is concentrated near the center of the particle and that the surrounding region with a larger ϕ_B^0 raises the overall effective barrier.

It should be mentioned that the limitations of this analytical model are: (1) the higher barrier region surrounding the metal patch is assumed to be semi-infinite; and (2) the effect of the SiO_x layer is neglected.

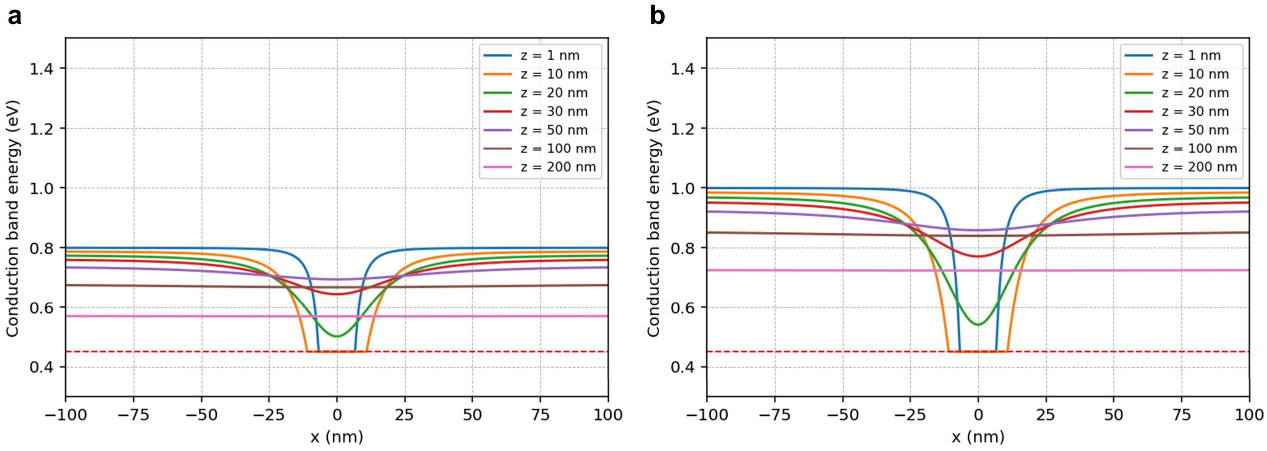

Figure S17. Spatial variation of CBM profiles across the metal patch (50 nm diameter) at varying semiconductor depths when (a) ϕ_B^0 = 0.8 eV and (b) ϕ_B^0 = 1.0 eV. The red dashed line is the cut-off region, which equals the barrier height at the metal patch. The center of the metal patch is used as the origin of *x*-axis.

Section 9 - Computational study on NiOOH work function

Computational method/details and general properties of bulk NiOOH and Ni(Fe)OOH

All computations were performed with the *Vienna Ab Initio* Simulation Package (VASP)[33] based on periodic spin-polarized density functional theory (DFT), using the projector-augmented-wave (PAW)[34] formalism to represent the core-valence electron interaction. The Perdrew-Burke-Ernzerhof (PBE)[35] exchange-correlation functional was used, supplemented by the dDsC dispersion correction[36] to account for van der Waals (vdW) interactions.

2×4×2 Bulk cell calculations were performed with a cutoff energy of 500 eV and a Monkhorst-Pack mesh of 3×3×2 *k*-point grid. H_2 was computed within a cubic box of 10 Å^3. The energy convergence criterion for the self-consistent-field (SCF) cycles was set to 10^{-6} eV per cell. As in our previous work,[37] the Hubbard correction as developed by Dudarev *et al.*[38] was set to a U-J value of 5.5 eV on Ni and Fe 3d orbitals in order not to introduce further energy differences for electron transfer reactions between metal atoms.[39,40] A Fermi smearing of 0.025 eV (290 K) was applied.

The computational hydrogen electrode (CHE)[41] was used to calculate the standard Gibbs free energy of the proton-electron couple in bulk models. The combined energy (H^+ + e^-) was replaced with half the Gibbs energy of H_2 in the gas phase at 0 V vs. RHE: $\mu_{H^+} + \mu_{e^-} = \frac{1}{2}\, G_{H_2}$.

The reaction Gibbs energy ΔG under standard conditions (pH = 0, T = 298 K, P = 1 bar) is expressed as:

$$\Delta G_x\ (U) = \Delta G_x + n\ (\mu_{H^+} + \mu_{e^-} - eU) \tag{16}$$

where ΔG_x represents the Gibbs energy of the reaction determined through DFT computation (including electronic energy (E), entropy (S), zero-point energy (ZPE) and an enthalpic correction ($\Delta H_{0\rightarrow T}$): $G = E + ZPE + \Delta H_{0\rightarrow T} - TS$), and n$eU$ is introduced when the potential differs from 0 V versus RHE (n the number of electron is strictly positive for an oxidation and strictly negative for a reduction, e the elementary charge, and U the electrode potential).

The pH of the solution controls the proton activity and the following relationship between reversible (RHE) and standard hydrogen electrode (SHE) is defined by:

$$U_{RHE} = U_{SHE} + k_B T \cdot \mathrm{pH} \ln(10) \tag{17}$$

The $M_xNi_{(1-x)}OOH$ slabs were modeled using a 2×4 supercell of the (0001) facet. We applied a symmetrization process to avoid surface dipole moments and to obtain unambiguous work functions. The final symmetric slabs were composed of four layers. To account for solvation effects, we used the implicit solvation model as implemented in VASPsol.[42] The model is based on the linearized Poisson-Boltzmann equation. It integrates an idealized electrolyte distribution into electronic structure calculations and neutralizes surface charge imbalances. Harmonic vibrational and thermal corrections to the Gibbs energy at 300 K have been determined using the VASPKIT package[43], where H_2 is considered as an ideal gas in the rigid rotor approximation. The electrochemical electrode potential was

taken into account for surface models with the grand-canonical DFT (GC-DFT) method. This method changes the number of electron in the computational unit-cell and determines the corresponding electrode potential via the Fermi-level.[44–47] The electrode's Gibbs energy (G) is computed as a function of the electrochemical potential (U):

$$G(U) = E(U) - q_{\mathrm{surf}}(U) \cdot U \approx E(U_0) - \frac{1}{2} C \cdot (U - U_0)^2 \tag{18}$$

where $E(U)$ is the corresponding electronic energy at a potential U, q_{surf} is the surface charge (positive when electrons are removed and negative when electrons are added), U_0 is the work function of the system at zero charge, and $E(U_0)$ is the energy used in the CHE framework. The second equivalence is used for metallic (gap-less) systems: the grand canonical energy $G(U)$ can be expanded to the second order with the capacitance of the system (C) as a coefficient.

In our previous work,[37] theoretical models of doped bulk structures of β-NiOOH and $Ni(OH)_2$ were investigated in order to assess the influence of transition metal substitution (including Fe) on the redox potential. The redox process was described by the reaction:

$$H^+ + e^- + Ni^{III}_{(1-x)}M^{III}_x(OOH) \rightarrow Ni^{III}_{(1-x)}M^{II}_x(OH)_2 \tag{19}$$

where x indicates the molar fraction of foreign metal M between 0.0625 to 0.25.

For the non-doped system, corresponding to the oxidation of $Ni(OH)_2$ into β-NiOOH, a redox potential of 1.46 V vs RHE was computed. The corresponding values for Fe-doped systems are summarized in **Table S5**. These results suggest that the redox potential decreases with an increasing Fe content, in contrast with experimental reports.[48] Our hypothesis to explain the discrepancy was that the assumption of a simultaneous oxidation of Fe and Ni was invalid. Therefore, we have here turned to sequential oxidation reactions, where Fe and Ni oxidations are decoupled. In particular, one expects Fe to be oxidized at lower potentials than Ni.

The free energy difference as a function of the electrochemical potential was computed using the following general equation "step-wise":

$$\Delta G(U) = G_{MO_2H_{1-n}} - G_{MOOH} + n(\mu_{H^+} + \mu_{e^-} - eU_{RHE}) \tag{20}$$

In particular, redox potentials have been calculated for:

25% Fe-doped bulk (**Figure S18**):

$[Ni(OH)_2]_{12}[Fe(OH)_2]_4 \rightarrow [Ni(OH)_2]_{12}[Fe(OH)_2]_2[FeOOH]_2 \rightarrow [Ni(OH)_2]_{12}[FeOOH]_4 \rightarrow [NiOOH]_{12}[FeOOH]_4$

50% Fe-doped bulk (**Figure S19**):

$[Ni(OH)_2]_8[Fe(OH)_2]_8 \rightarrow [Ni(OH)_2]_8[FeOOH]_8 \rightarrow [NiOOH]_8[FeOOH]_8$

For the 25% Fe-doped system, the reduced bulk $[Ni(OH)_2]_{12}[Fe(OH)_2]_4$ is the most stable below 0.6 V vs RHE. Between 0.6 and 1.48 V vs RHE, the most stable configuration is $[Ni(OH)_2]_{12}[FeOOH]_4$, where Fe is oxidized while Ni remains reduced. Above 1.48 V vs RHE, complete oxidation of all metal centers occurs. The full oxidation of the bulk therefore takes place at 1.48 V vs RHE, slightly higher than in the pure Ni system, and in qualitative agreement with experimental data.

For the 50% Fe-doped bulk, the stepwise oxidation indicates that Fe is oxidized at 0.78 V vs RHE, followed by Ni oxidation at 1.39 V vs RHE. In this case, the overall redox potential is decreased compared to pure Ni, in disagreement with experimental observations. At these high Fe concentrations, there is a significant interaction between the Fe and Ni metal centers, rationalizing the smaller difference between the redox potential of the individual metal centers. These results suggest that a 25% Fe doping level provides the best agreement with experimental data.

Table S5. Calculated redox potentials for Ni bulk doped with different amounts of iron using **Eq. (19)**.[37]

**Fe percentage (%)**	**0**	**6.25**	**12.5**	**18.75**	**25**
**Redox potential (V vs RHE)**	1.46	1.42	1.36	1.31	1.26

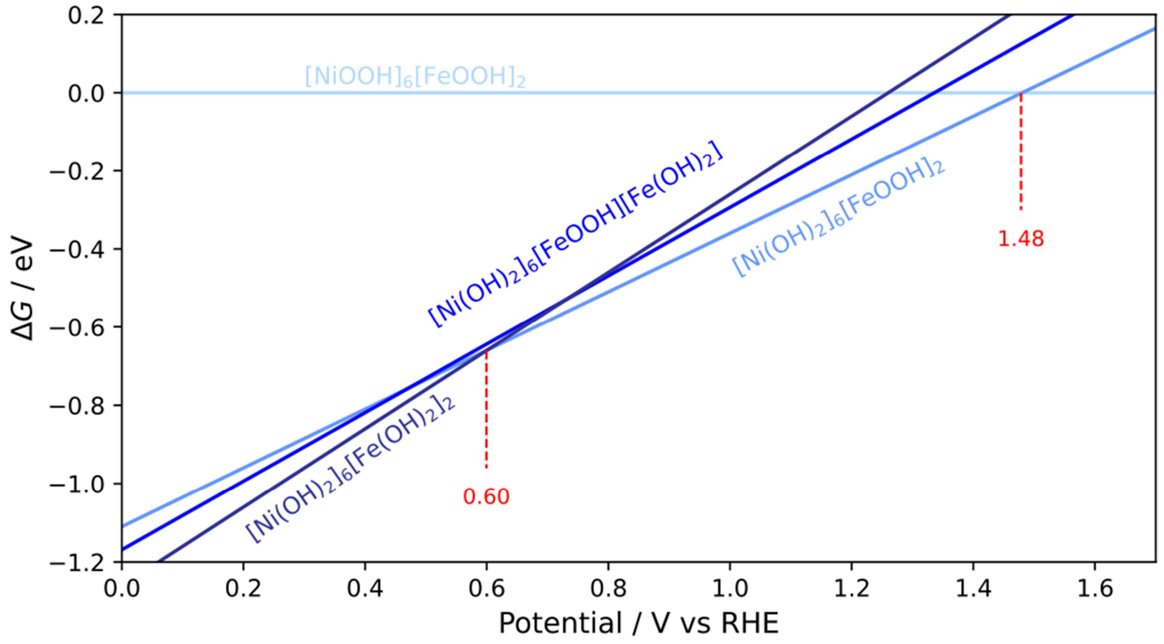

Figure S18. Relative stability of the oxidation states of 25% Fe-doped Ni bulk as a function of the electrochemical potential, calculated using **Eq. (20)**. The color scale ranges from dark blue (most reduced form) to light blue (all metal atoms oxidized). The lowest graph at each potential corresponds to the most stable bulk configuration.

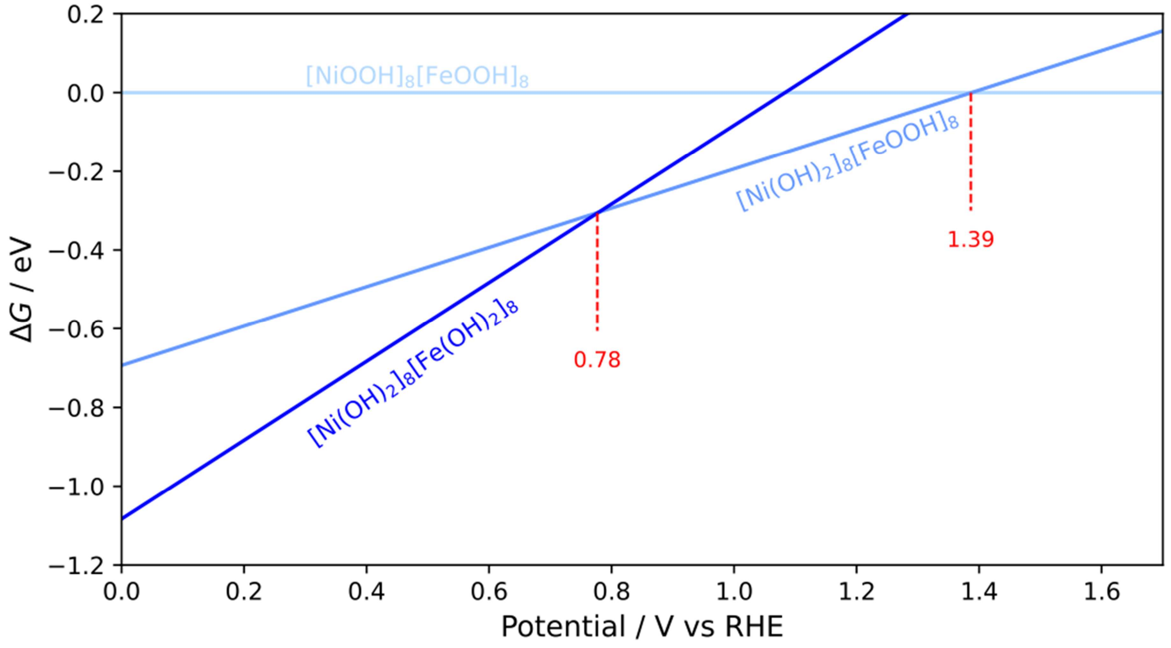

Figure S19. Relative stability of the oxidation states of 50% Fe-doped Ni bulk as a function of the electrochemical potential, calculated using **Eq. (20)** The color scale ranges from dark blue (most reduced form) to light blue (all metal atoms oxidized). The lowest curve at each potential corresponds to the most stable bulk configuration.

Work function calculations of NiOOH under the influence of Fe incorporation

In order to investigate the influence of Fe substitution in NiOOH at the atomic level, we have performed a computational study at the spin-polarized DFT+U level of theory. Starting from our validated methodology for NiOOH and its surface state,[37] we first assessed the bulk redox properties of Fe-substituted NiOOH. In contrast to our earlier work, we have studied the sequential redox-properties of Fe (usually easier to oxidize) and Ni centers (as discussed in the above section). At our level of theory, the pure $Ni(OH)_2$ oxidation occurs at 1.46 V vs RHE. In the presence of 25% Fe in the bulk, Fe is oxidized at 0.60 V and the Ni atoms only at 1.48 V vs RHE, in qualitative agreement with the experiments reported here and earlier.[48] A comparison with higher (50%) bulk Fe content reveals that Fe is, as expected, still easily oxidized. Nevertheless, at this high Fe content, the coupling between Fe and Ni centers is stronger (Fe oxidation at 0.78 V and Ni oxidation at 1.39 V vs RHE). This indicates that the Fe content in experimental samples should be around 25%, which was also suggested based on stability measurements.[49] However, a change of ~20 mV in the bulk redox potential does not explain the observed increase in the work function of ~0.4 eV. Hence, we have also performed an extensive study of the surface state of Fe-substituted NiOOH.

Given the operando enrichment of Fe, the surface concentration of Fe might be higher than in the bulk. Therefore, we have tested various surface models with 25% to 62.5% Fe, see **Figure S20**. As we have observed a significantly easier oxidation of Fe compared to Ni in the bulk, we have also investigated the oxidation of the surface as a function of the electrochemical potential. In these studies, the electrochemical potential is explicitly modelled via grand-canonical DFT, which also allows to distinguish oxidation from deprotonation reactions.[50] All surfaces are built considering a NiOOH-like bulk phase. The stoichiometric MOOH surfaces are systematically taken as the reference in order to compare their relative propensity to get oxidized. Furthermore, we have opted to focus the comparison on a potential of 1.5 V vs RHE. At this potential the MOOH bulk phase is stable for all considered cases and it is close to the working conditions of the materials. Therefore, this potential is representative of the formation of the active phase. The most stable surface terminations at this potential are slightly oxidized (37 to 25% hydrogen instead of 50% hydrogen when compared to the number of oxygen site), with one exception (8FeB model, where the surface is iron rich, but the sub-surface also contains some iron atoms). When the surface is fully substituted by Fe, the hydrogen coverage is lowest (25%) and the work function can increase up to 6.5 eV. The high work function and the relative energies of 8FeA vs 8FeB model (0.7 to 1.4 eV in favor of 8FeB, with the energy difference increasing with increasing hydrogen coverage) indicate that the surface Fe concentration should not exceed 75%. Interestingly, the work functions of the most stable surface state at 1.5 V vs RHE are indistinguishable for Fe concentrations of 25-37%. Even at these lower Fe contents, Fe is more stable in the sub-surface than in the surface. However, the enrichment process makes surface enrichment kinetically much more credible. Hence, we conclude that the surface concentration of ~50% in the model with an overall Fe content of ~25% is most representative of the obtained material. The increase in work function upon Fe incorporation is 1 eV (NiOOH has a computed work function of 4.8 eV), overestimating the experimentally measured values. This suggests that either the average surface concentration of Fe is slightly lower or the hydrogen

coverage slightly higher. Nevertheless, the origin of the increased work function of Fe-substituted NiOOH surfaces is unambiguously identified as the combined effects of Fe present in the surface, which in turn causes a decrease in the number of hydrogen atoms on the surface. It is this surface hydrogen coverage that causes the substantial change in work functions observed experimentally.

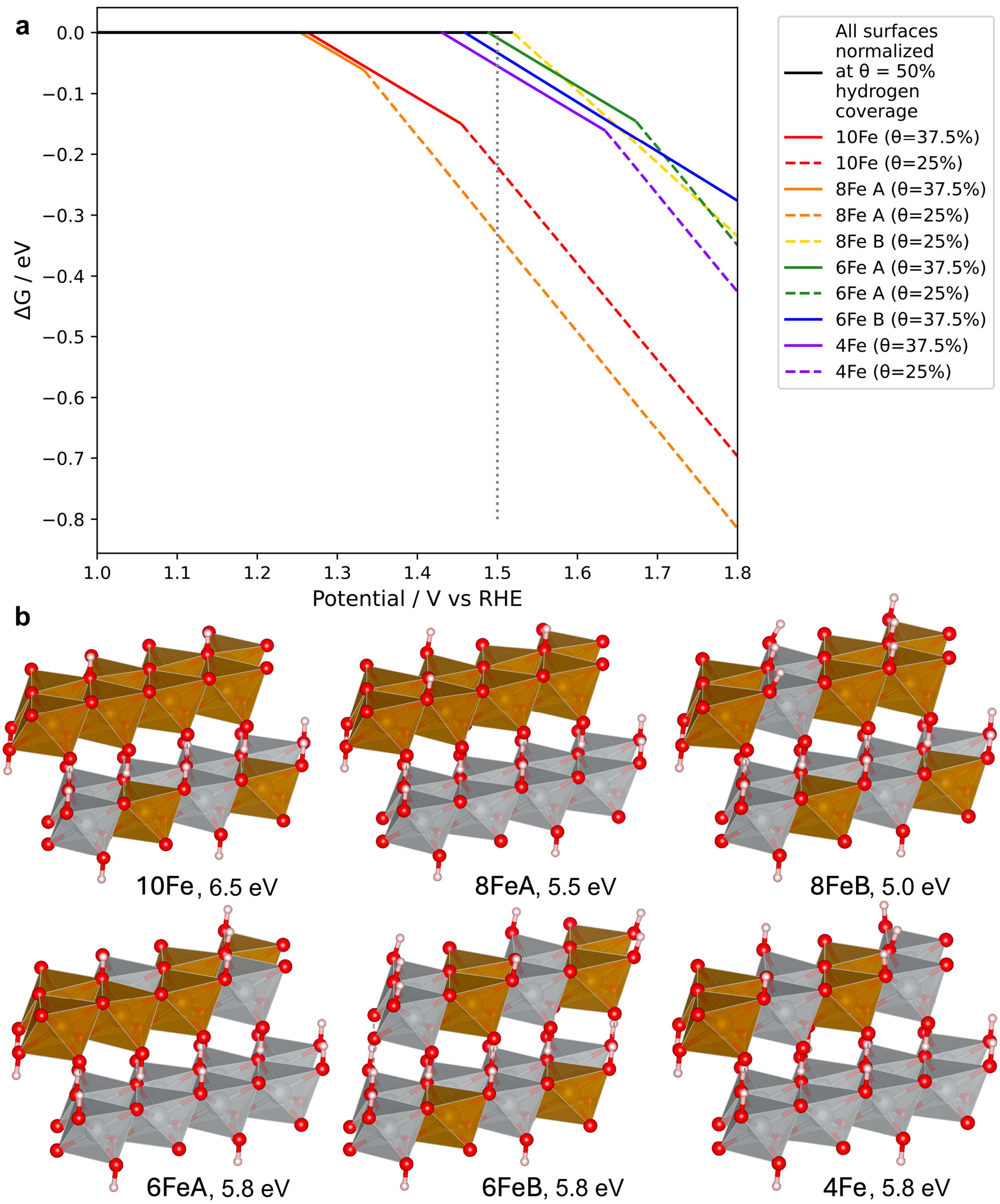

Figure S20. (a) Relative stability of iron-doped surfaces as a function of hydrogen coverage (θ) and the electrochemical potential at pH = 14. Fe contents range from 25% (4 Fe) to 62.5% (10 Fe) The stoichiometric MOOH surface (θ = 50%) is taken as the reference. (b) Three dimensional representations of the different relevant configurations depending on the location of iron atoms at a potential of 1.5 V vs RHE [vertical dotted line in panel (a)] in the (sub-)surface layer; Ni in grey, Fe in brown, O in red, and H in white. The work function of the corresponding surface is given after its label.

Section 10 - Effects of Fe on Co NPs modified n-Si photoanode

We have performed the analysis of Fe incorporation effect on Co NPs-modified Si photoanode to further generalize our studies and partly demonstrate that it could apply to different metals. Co NPs are deposited on n-Si surface through electrodeposition following the literature.[51] ECD is varied to obtain different NP size and density. n-Si/SiO_x/CoNPs-x, where x is ECD in mC cm^{-2}, with different morphology were successfully fabricated, as shown in the inset of **Figure S21**. For the electrolyte purification, $Co(OH)_2$ was utilized as the Fe adsorber/remover instead of $Ni(OH)_2$ to avoid the potential effects of Ni impurities on Co-based materials.[52] As shown in **Figure S21**, the electrocatalytic performance of Co NPs on conductive p^+-Si improves in the presence of Fe impurities, coinciding with previous literature.[52–54] We also notice that the intrinsic electrocatalytic activity of pure Co NPs toward water oxidation is better than that of pure Ni NPs. The PEC performance of n-Si photoanode was found to also greatly increase in the Fe-containing electrolyte and depend on the morphology of Co NPs. The calculated photovoltages, reported in **Table S6,** increased when Fe was intentionally added, especially in the case of smaller nanoparticles (70 mV difference between purified and 1.0 ppm Fe-containing electrolytes). The results obtained in this experiment are consistent with the studies on Ni NPs and crucially suggest that the effects we are observing might be potentially applicable to other metals or junctions.

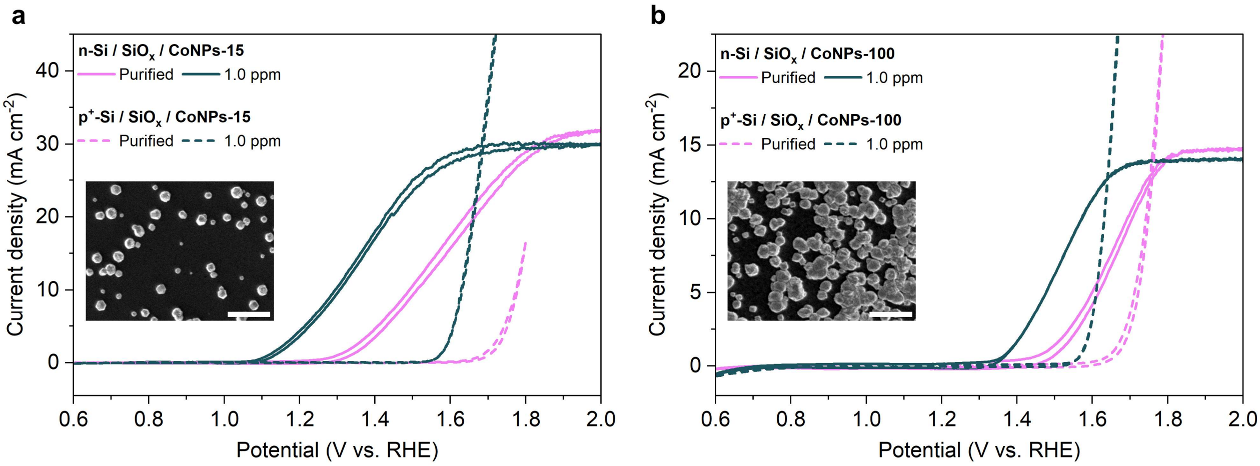

Figure S21. CVs recorded on (a) n-Si/SiO_x/CoNPs-15 under 1-sun illumination and p^+-Si/SiO_x/CoNPs-15 in the dark and (b) n-Si/SiO_x/CoNPs-100 under 1-sun illumination and p^+-Si/SiO_x/CoNPs-100 in the dark, either in purified or 1.0 ppm Fe-containing 1 M KOH electrolyte. The inset pictures show top view SEM images of Co NPs electrodeposited with an ECD of 15 and 100 mC cm^{-2}. The scale bar equals 500 nm.

Table S6. Onset potential (E_{on}) of p^+-Si/SiO_x/CoNPs-x (EC, in the dark) and n-Si/SiO_x/CoNPs-x (PEC, under 1-sun illumination) and calculated photovoltage (V_{ph}) from 1 M KOH electrolytes, either purified or containing 1.0 ppm Fe.

ECD (mC cm^{-2})	Electrolyte	E_{on} (EC) / V vs. RHE	E_{on} (PEC) / V vs. RHE	V_{ph} / V
15	Purified	1.683	1.299	0.384
	1.0 ppm Fe	1.572	1.116	0.456
100	Purified	1.655	1.480	0.175
	1.0 ppm Fe	1.574	1.379	0.195

Section 11 - Fe leaching experiment

Each n-Si/SiO_x/NiNPs photoanode and the non-photoactive p^+-Si/SiO_x/NiNPs counterpart were first activated in an electrolyte containing 0.5 ppm Fe^{3+} and subsequently cycled in the purified electrolyte for 200 cycles. It was found that the electrochemical and PEC performance (indicated by E_{on} and j) of the electrodes decreased drastically over the first 20 potential scans when Fe^{3+} was absent in the electrolyte as shown in **Figure S22a** and **b** for p^+-Si/SiO_x/NiNPs-5 and n-Si/SiO_x/NiNPs-5, respectively. After that, the performance continuously decreased over several potential scans. When compared to the reference anode (see **Figure 1b-c**), this indicates that the incorporation of Fe is reversible. In the close-up regions of **Figure S22** (bottom panels), it can be observed that E_{peak} gradually shifted back (i.e., cathodically for the p^+-Si anode and anodically for the n-Si photoanode), approaching the value measured for similar electrodes activated in the purified electrolyte (indicated here as pink dashed lines). Based on our proposed mechanism for Fe incorporation discussed in the main section, this can be explained based on Fe leaching from the Ni(Fe)$(OH)_2$/Ni(Fe)OOH shell when the electrodes were operated in the purified electrolyte.

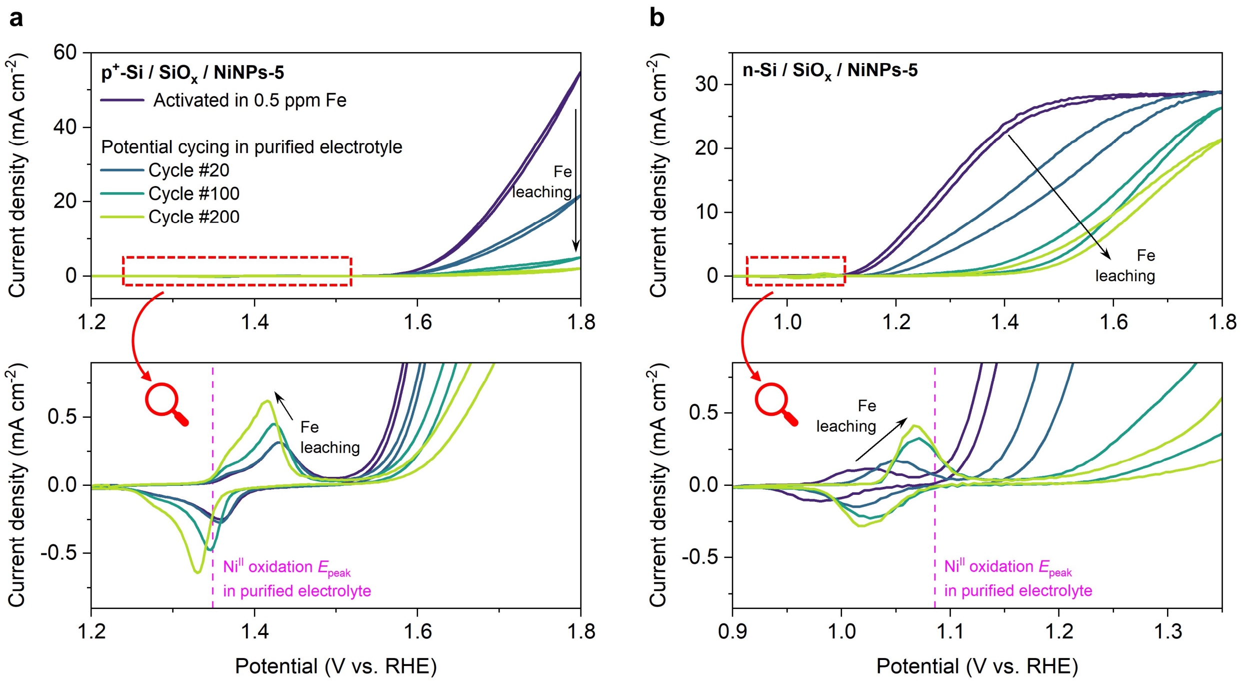

Figure S22. CV scans measured for (a) p^+-Si/SiO_x/NiNPs-5 in the dark and (b) n-Si/SiO_x/NiNPs-5 under 1-sun illumination after activation in 0.5 ppm Fe electrolyte and then cycled in purified electrolyte (1 M KOH) for 200 scans. The bottom panels show the close-up of the regions marked with dashed boxes. The pink dashed lines indicate the position of the Ni^{II} oxidation peak (E_{peak}) for the respective electrodes activated in the purified electrolyte. Scan rate = 100 mV s^{-1}.

From **Figures S23** and **S24**, it can be noted that Fe incorporation process appeared less reversible for NiNPs-50 and NiNPs-200 (larger and denser Ni NPs). In any case, OER was strongly affected, and as Fe leached out, E_{peak} varied in the cathodic direction for all cases of the large NPs.

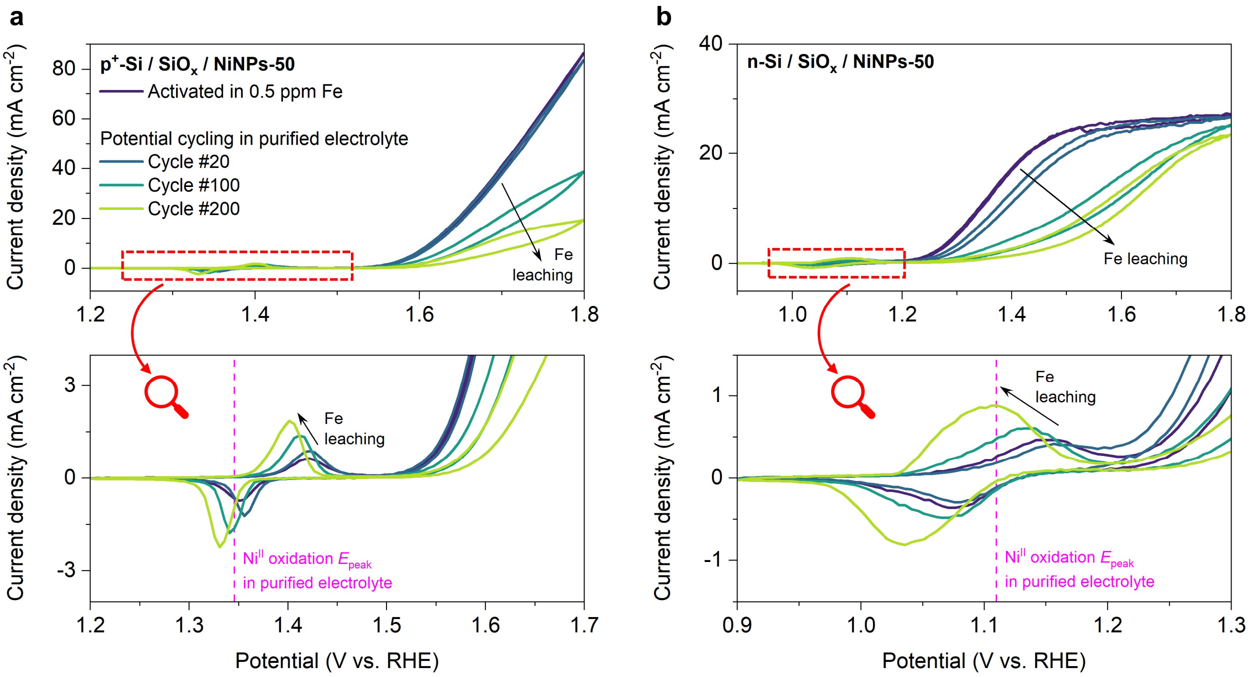

Figure S23. CV scans measured for (a) p⁺-Si/SiOₓ/NiNPs-50 in the dark and (b) n-Si/SiOₓ/NiNPs-50 under 1-sun illumination after activation in 0.5 ppm Fe electrolyte and then cycled in purified electrolyte (1 M KOH) for 200 scans. The bottom panels show the close-up of the regions marked with dashed boxes. The pink dashed lines indicate the position of the Ni^{II} oxidation peak (E_{peak}) for the respective electrodes activated in the purified electrolyte. Scan rate = 100 mV s^{-1}.

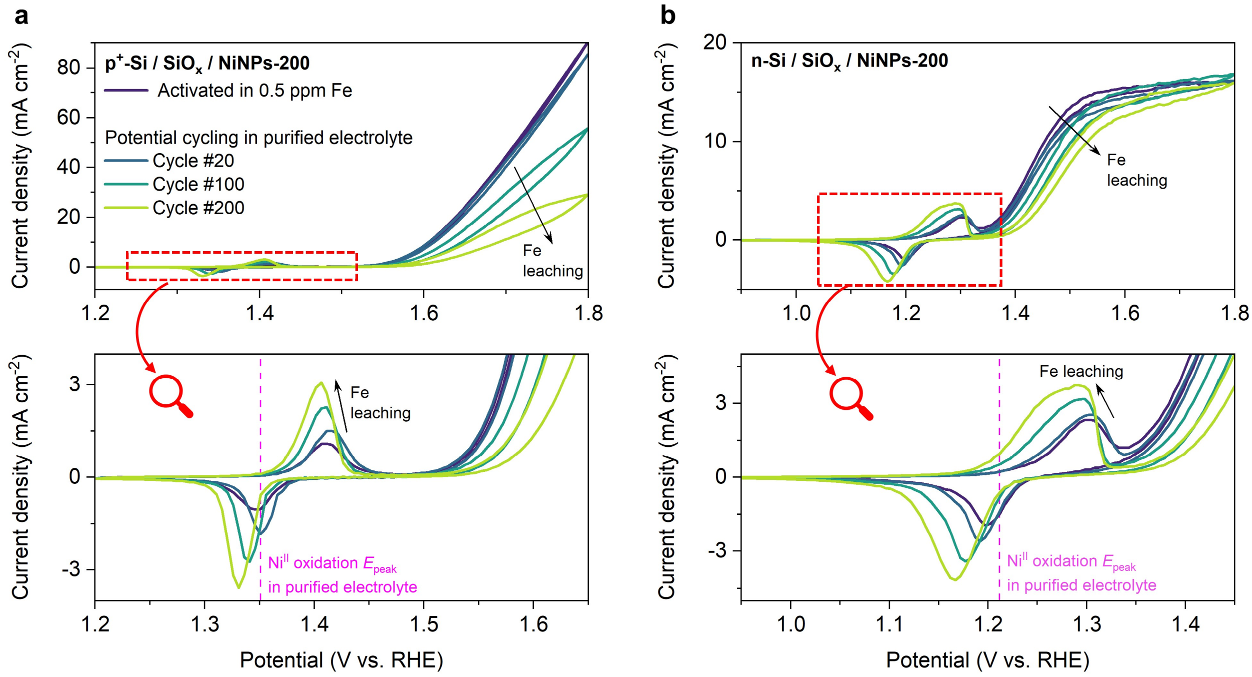

Figure S24. CV scans measured for (a) p⁺-Si/SiOₓ/NiNPs-200 in the dark and (b) n-Si/SiOₓ/NiNPs-200 under 1-sun illumination after activation in 0.5 ppm Fe electrolyte and then cycled in purified electrolyte (1 M KOH) for 200 scans. The bottom panels show the close-up of the regions marked with dashed boxes. The pink dashed lines indicate the position of the Ni^{II} oxidation peak (E_{peak}) for the respective electrodes activated in the purified electrolyte. Scan rate = 100 mV s^{-1}.

Section 12 - Supplementary tables

Table S7. Sample mass, Fe content quantified by ICP-MS, and their ratios of different samples. The substrates were n-Si for all samples.

Sample	Sample mass (g)	Fe content (μg L^{-1})	Fe content/sample mass (μg L^{-1} g^{-1})
Bare n-Si	0.1368	0.19	1.389
NiNPs-5 (As-deposited)	0.1781	0.50	2.807
NiNPs-5 (Activated in 1.0 ppm Fe)	0.2026	1.42	7.009
NiNPs-5 (Activated in 10 ppm Fe)	0.2206	3.84	17.407
NiNPs-200 (Activated in 10 ppm Fe)	0.1913	5.20	27.182

Table S8. Onset potential (E_{on}) of p^+-Si/SiO_x/NiNPs-5 (EC, in the dark) and n-Si/SiO_x/NiNPs-5 (PEC, under 1-sun illumination) and calculated photovoltage (V_{ph}) from 1 M KOH electrolytes containing varying Fe concentrations. The units of E_{on} are [V vs. RHE].

Sample No.	Purified		0.1 ppm		0.5 ppm		1.0 ppm	
	E_{on} (EC)	E_{on} (PEC)	E_{on} (EC)	E_{on} (PEC)	E_{on} (EC)	E_{on} (PEC)	E_{on} (EC)	E_{on} (PEC)
1	1.787	1.399	1.576	1.137	1.551	1.095	1.542	1.085
2	1.795	1.401	1.571	1.137	1.564	1.108	1.562	1.081
3	1.782	1.370	1.571	1.124	1.555	1.081	1.562	1.090
4						1.068		1.081
5						1.070		1.071
**V_{ph} / mV:**	**388 ± 21**		**434 ± 10**		**474 ± 23**		**480 ± 17**	

Table S9. Onset potential (E_{on}) of p^+-Si/SiO_x/NiNPs-50 (EC, in the dark) and n-Si/SiO_x/NiNPs-50 (PEC, under 1-sun illumination) and calculated photovoltage (V_{ph}) from different electrolytes.

Fe concentration / ppm	E_{on} (EC) / V vs. RHE	E_{on} (PEC) / V vs. RHE	V_{ph} / mV
Purified	1.714	1.426	288
0.1	1.551	1.245	306
0.5	1.523	1.232	291
1.0	1.522	1.230	292

Table S10. Onset potential (E_{on}) of p^+-Si/SiO_x/NiNPs-200 (EC, in the dark) and n-Si/SiO_x/NiNPs-200 (PEC, under 1-sun illumination) and the calculated photovoltage (V_{ph}) from different electrolytes.

Fe concentration / ppm	E_{on} (EC) / V vs. RHE	E_{on} (PEC) / V vs. RHE	V_{ph} / mV
Purified	1.763	1.546	217
0.1	1.585	1.362	223
0.5	1.542	1.350	192
1.0	1.541	1.324	217

Table S11. Summary of the photovoltage reported in the literature on Si photoanodes with Ni NPs.

Particle diameter / nm	Photovoltage / mV	Measured by	Ref.
60[a]	500	E_{on}[c]	[1]
50[a]	500	E_{on}[c]	[3]
40-80[b]	500-450	PS-EC-AFM[d]	[4]
35-50[b]	480	E_{on}[c]	[6]
35[a]	480	E_{on}[c]	[55]
35-55[b]	425-455	E_{on}[c]	[56]

[a] Particle diameter reported as an average value.

[b] Particle diameter reported as a diameter range.

[c] Photovoltage was calculated from the difference in the onset potential (E_{on}) obtained on p^+-Si and n-Si.

[d] Photovoltage was measured on individual particles using potential-sensing electrochemical AFM (PS-EC-AFM).

Section 13 - Supplementary figures

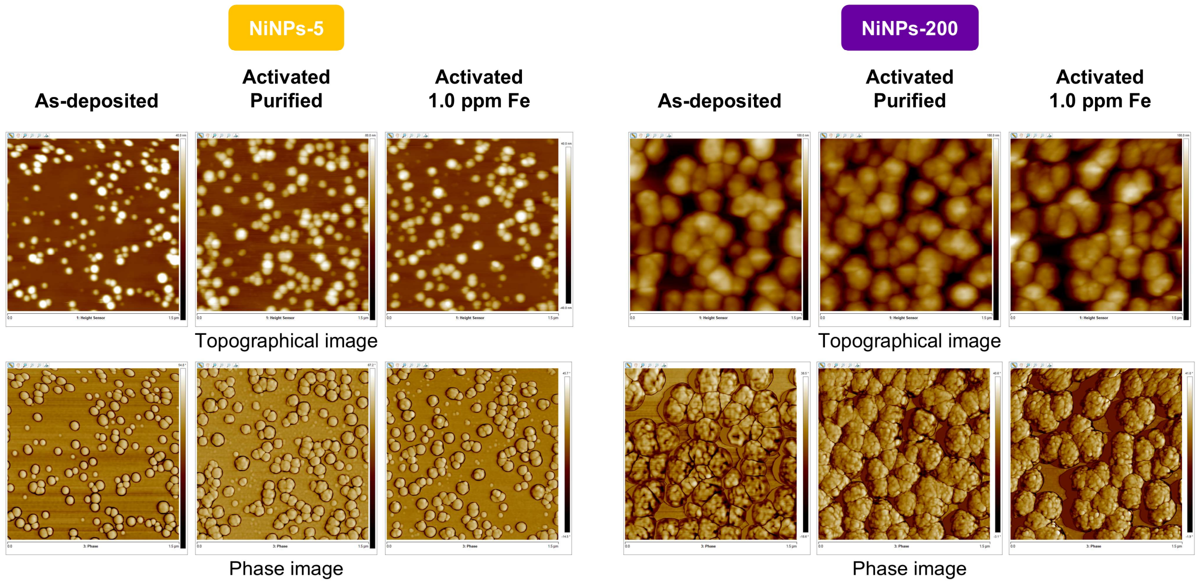

Figure S25. AFM topographical and phase images of NiNPs-5 and NiNPs-200 on n-Si prepared under different conditions.

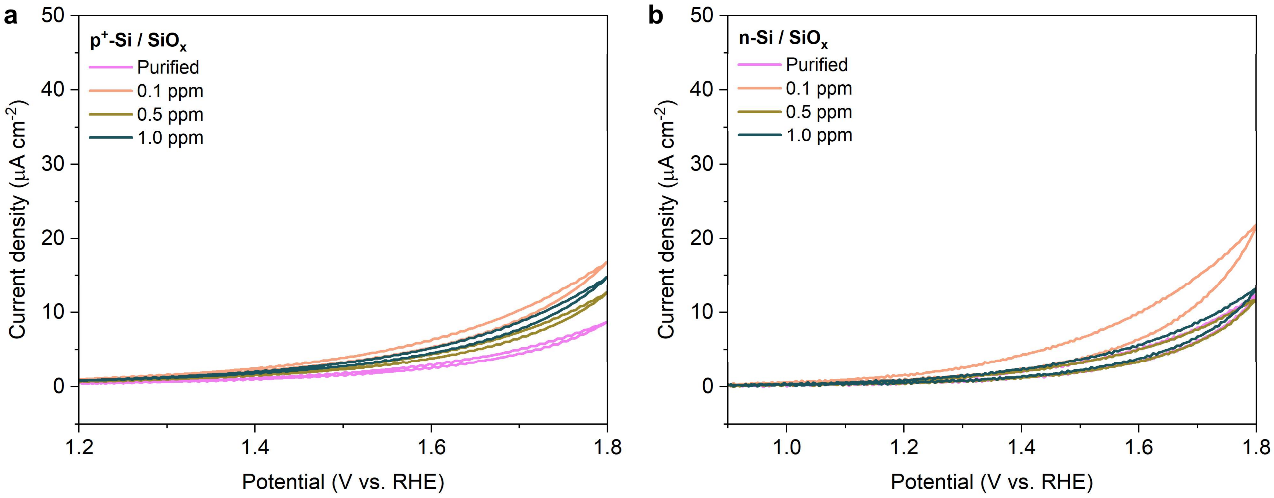

Figure S26. Cyclic voltammograms of (a) bare p⁺-Si in the dark and (b) bare n-Si under 1-sun illumination in different electrolytes. Note that the current density scale is in μA cm^{-2}.

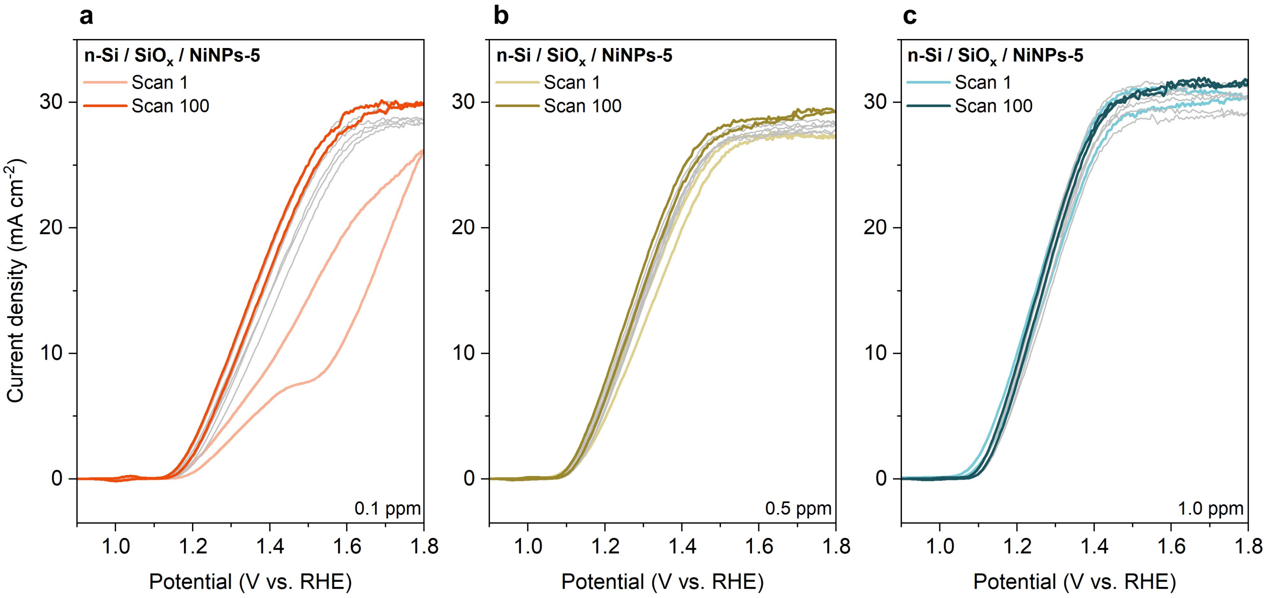

Figure S27. Cyclic voltammograms of n-Si/SiOx/NiNPs-5 under 1-sun illumination in 1 M KOH electrolyte with different Fe concentrations of (a) 0.1 ppm, (b) 0.5 ppm, and 1.0 ppm. Scans #1 and #100 are shown as light and dark colors, respectively. Intermediate scans are shown in grey.

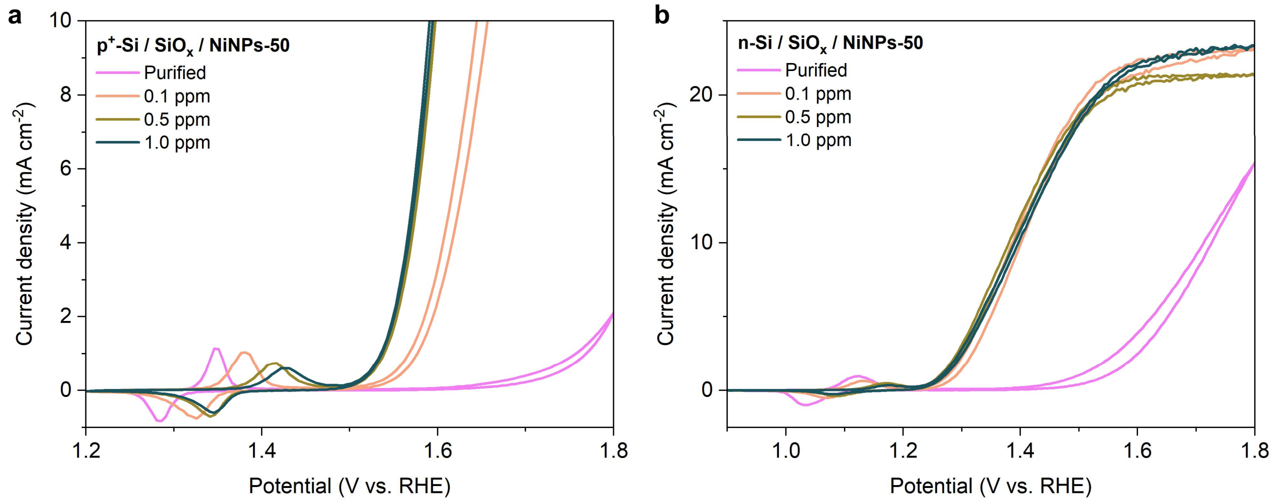

Figure S28. Cyclic voltammograms of (a) p^+-Si/SiOx/NiNPs-50 in the dark and (b) n-Si/SiOx/NiNPs-50 under 1-sun illumination in different electrolytes.

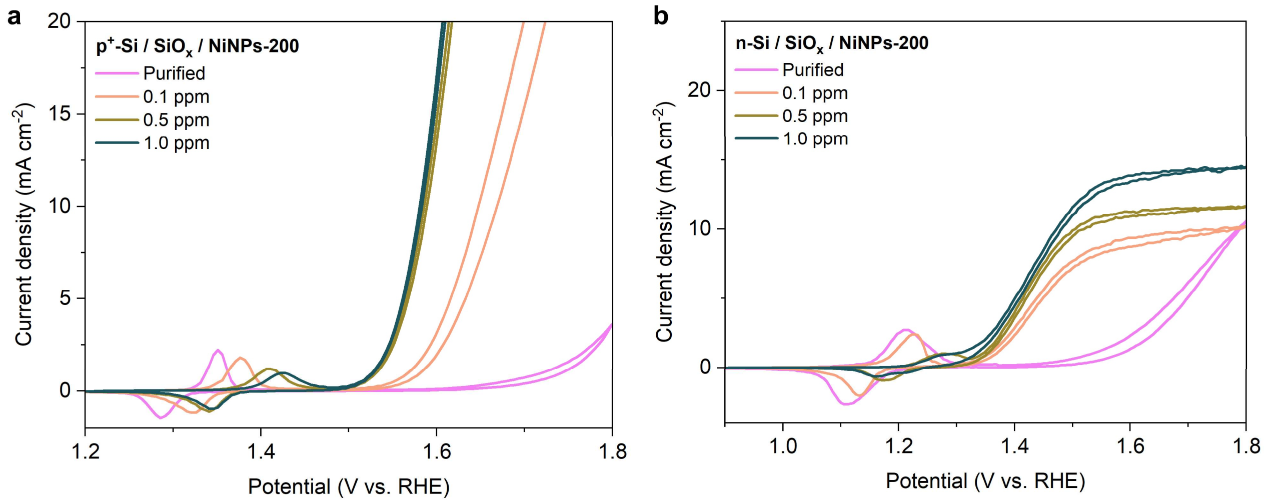

Figure S29. Cyclic voltammograms of (a) p^+-Si/SiO_x/NiNPs-200 in the dark and (b) n-Si/SiO_x/NiNPs-200 under 1-sun illumination in different electrolytes.

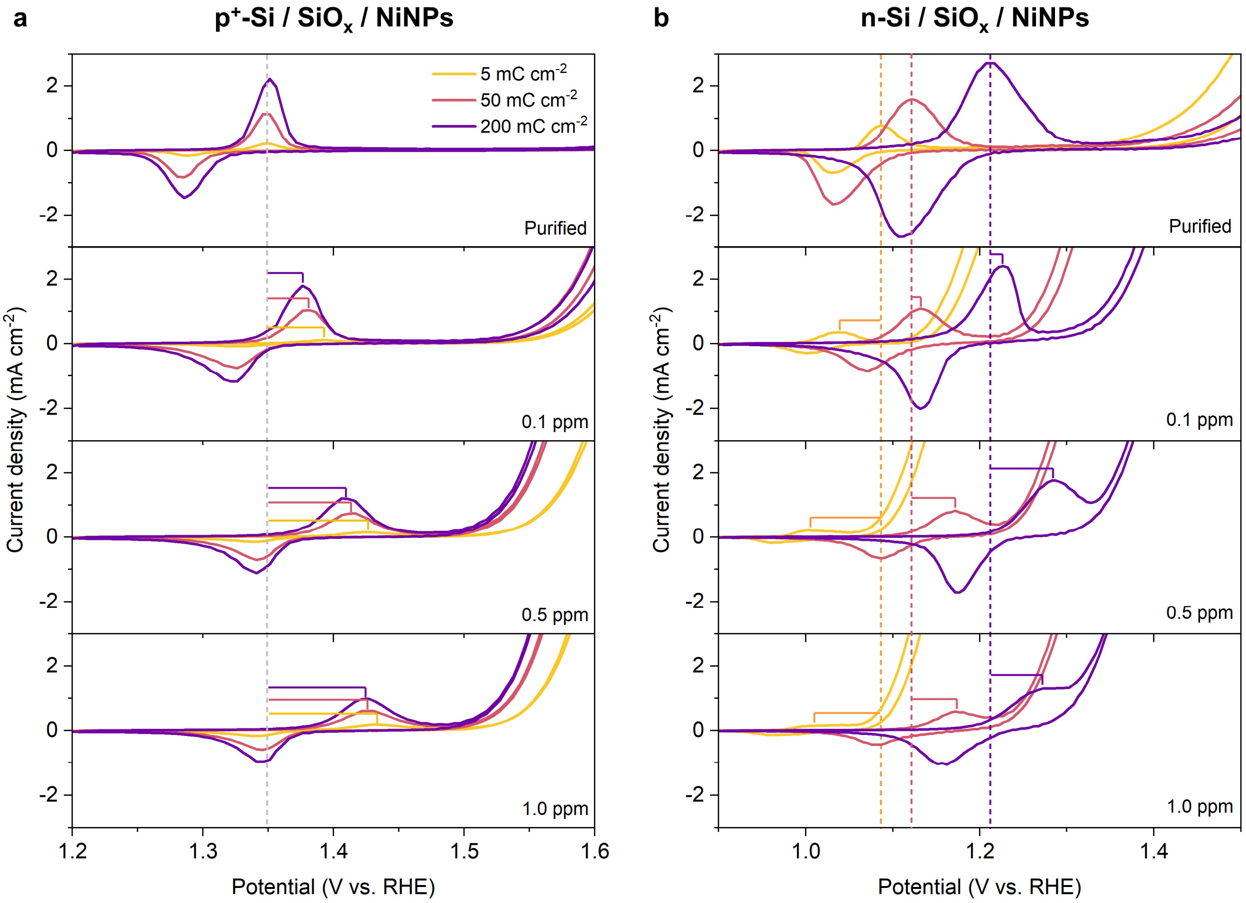

Figure S30. Comparison of the Ni^{III}/Ni^{II} oxidation peak in different electrolytes for (a) p^+-Si/SiO_x/NiNPs in the dark and (b) n-Si/SiO_x/NiNPs under 1-sun illumination.

<u>*Notes on Figure S16:*</u>

For p^+-Si/SiO_x/NiNPs in the purified electrolyte, the Ni^{II} oxidation peak position (E_{peak}) of each sample was located around the same potential. This could indicate similar properties of $Ni(OH)_2$/NiOOH layer since there was no interference from Fe impurities. As the Fe concentration increased, E_{peak} shifted progressively in the anodic direction. Interestingly, the shift was inversely proportional to the size of NPs. With smaller NPs, E_{peak} was at a higher potential, suggesting a more prominent change in the outer layer. This could be because the proportion of Fe-doped shell, i.e., $Ni(Fe)(OH)_2$/Ni(Fe)OOH, of small NPs is higher than that of larger NPs. For n-Si/SiO_x/NiNPs, E_{peak} in the purified electrolyte was located at different potentials due to the different photovoltages. With the increasing Fe concentration, the changes in E_{peak} for NiNPs-50 and NiNPs-200 followed the same trend as observed on p^+-Si. In contrast, for NiNPs-5, E_{peak} was shifted *cathodically*, highlighting the strong photovoltaic enhancement. The latter was achievable only with the presence of Fe and with small NPs.

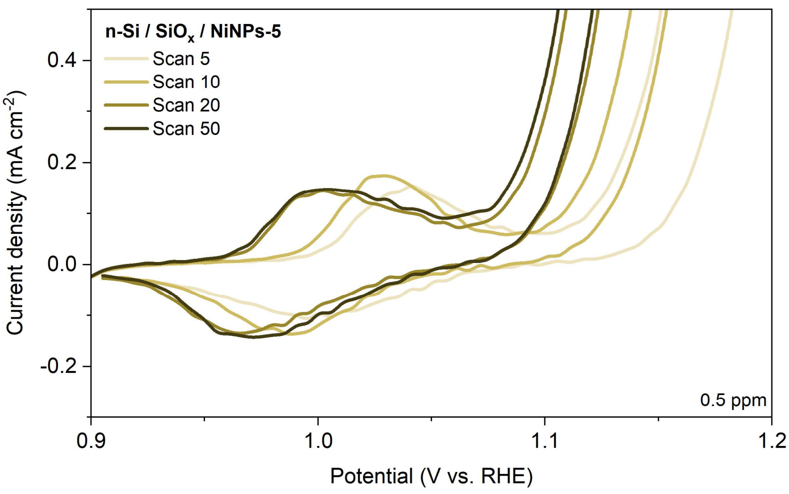

Figure S31. Cyclic voltammograms recorded from n-Si/SiO_x/NiNPs-5 during the activation in 0.5 ppm Fe-containing electrolyte, from the 5th to 50th scan showing the cathodic shift of E_{peak} position during the potential cycling due to Fe incorporation.

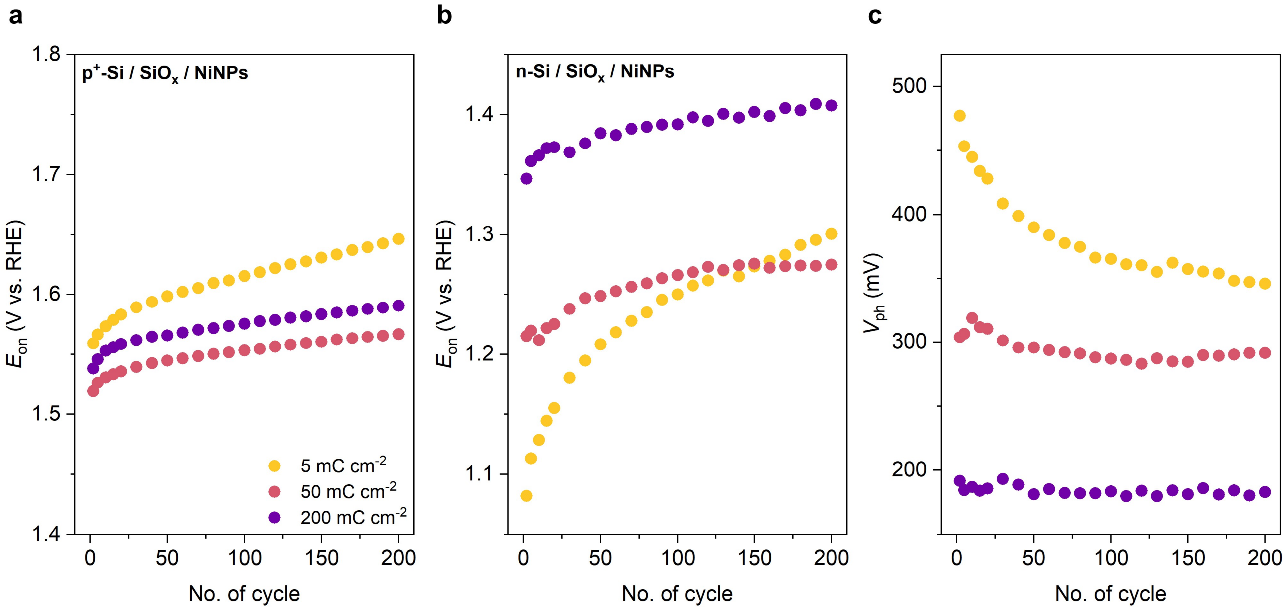

Figure S32. Onset potential as a function of the number of potential cycles for (a) p$^+$-Si/SiO_x/NiNPs in the dark and (b) n-Si/SiO_x/NiNPs under 1-sun illumination in the purified electrolyte *after* activation in 0.5 ppm Fe-containing electrolyte. (c) Photovoltage as a function of the number of potential cycles for samples prepared with different ECDs.

Section 14 - References

(1) Loget, G.; Fabre, B.; Fryars, S.; Mériadec, C.; Ababou-Girard, S. Dispersed Ni Nanoparticles Stabilize Silicon Photoanodes for Efficient and Inexpensive Sunlight-Assisted Water Oxidation. *ACS Energy Lett.* **2017**, *2* (3), 569–573. https://doi.org/10.1021/acsenergylett.7b00034.

(2) Oh, K.; Mériadec, C.; Lassalle-Kaiser, B.; Dorcet, V.; Fabre, B.; Ababou-Girard, S.; Joanny, L.; Gouttefangeas, F.; Loget, G. Elucidating the Performance and Unexpected Stability of Partially Coated Water-Splitting Silicon Photoanodes. *Energy Environ. Sci.* **2018**, *11* (9), 2590–2599. https://doi.org/10.1039/c8ee00980e.

(3) Lee, S. A.; Lee, T. H.; Kim, C.; Lee, M. G.; Choi, M. J.; Park, H.; Choi, S.; Oh, J.; Jang, H. W. Tailored NiO_x/Ni Cocatalysts on Silicon for Highly Efficient Water Splitting Photoanodes via Pulsed Electrodeposition. *ACS Catal.* **2018**, *8* (8), 7261–7269. https://doi.org/10.1021/acscatal.8b01999.

(4) Laskowski, F. A. L.; Oener, S. Z.; Nellist, M. R.; Gordon, A. M.; Bain, D. C.; Fehrs, J. L.; Boettcher, S. W. Nanoscale Semiconductor/Catalyst Interfaces in Photoelectrochemistry. *Nat. Mater.* **2020**, *19*, 69–76. https://doi.org/10.1038/s41563-019-0488-z.

(5) Zhou, X.; Liu, R.; Sun, K.; Papadantonakis, K. M.; Brunschwig, B. S.; Lewis, N. S. 570 MV Photovoltage, Stabilized n-Si/CoO_x Heterojunction Photoanodes Fabricated Using Atomic Layer Deposition. *Energy Environ. Sci.* **2016**, *9* (3), 892–897. https://doi.org/10.1039/C5EE03655K.

(6) Hemmerling, J. R.; Mathur, A.; Linic, S. Characterizing the Geometry and Quantifying the Impact of Nanoscopic Electrocatalyst/Semiconductor Interfaces under Solar Water Splitting Conditions. *Adv. Energy Mater.* **2022**, *12* (11), 2103798. https://doi.org/10.1002/aenm.202103798.

(7) Chuang, C. H.; Kang, P. H.; Lai, Y. Y.; Hou, C. H.; Cheng, Y. J. Junction Engineering in Si Photoanodes for Efficient Photoelectrochemical Water Splitting. *ACS Appl. Energy Mater.* **2022**, *5* (7), 8483–8491. https://doi.org/10.1021/acsaem.2c00974.

(8) Loget, G.; Mériadec, C.; Dorcet, V.; Fabre, B.; Vacher, A.; Fryars, S.; Ababou-Girard, S. Tailoring the Photoelectrochemistry of Catalytic Metal-Insulator-Semiconductor (MIS) Photoanodes by a Dissolution Method. *Nat. Commun.* **2019**, *10*, 3522. https://doi.org/10.1038/s41467-019-11432-1.

(9) Hemmerling, J.; Quinn, J.; Linic, S. Quantifying Losses and Assessing the Photovoltage Limits in Metal–Insulator–Semiconductor Water Splitting Systems. *Adv. Energy Mater.* **2020**, *10* (12), 1903354. https://doi.org/10.1002/aenm.201903354.

(10) Aroonratsameruang, P.; Klahan, K.; Loget, G.; Pattanasattayavong, P. Enhancing Photovoltage of Silicon Photoanodes by a High Work-Function Coordination Polymer. *J. Phys. Chem. C* **2022**, *126* (37), 15587–15595. https://doi.org/10.1021/acs.jpcc.2c04274.

(11) Li, Y.; Ding, C.; Li, Y.; Zeng, J.; Kang, C.; Chen, H.; Wang, L.; He, J.; Li, C. Engineering the Inhomogeneity of Metal–Insulator–Semiconductor Junctions for Photoelectrochemical Methanol Oxidation. *ACS Appl. Mater. Interfaces* **2023**, *15* (51), 59403–59412. https://doi.org/10.1021/acsami.3c12957.

(12) Loget, G. Water Oxidation with Inhomogeneous Metal-Silicon Interfaces. *Curr. Opin. Colloid Interface Sci.* **2019**, *39*, 40–50. https://doi.org/10.1016/j.cocis.2019.01.001.

(13) Xu, G.; Xu, Z.; Shi, Z.; Pei, L.; Yan, S.; Gu, Z.; Zou, Z. Silicon Photoanodes Partially Covered by $Ni@Ni(OH)_2$ Core–Shell Particles for Photoelectrochemical Water Oxidation. *ChemSusChem* **2017**, *10* (14), 2897–2903. https://doi.org/10.1002/cssc.201700825.

(14) Liu, D.; Jiang, T.; Liu, D.; Zhang, W.; Qin, H.; Yan, S.; Zou, Z. Silicon Photoanode Modified with Work-Function-Tuned $Ni@Fe_yNi_{1-y}(OH)_2$ Core-Shell Particles for Water Oxidation. *ChemSusChem* **2020**, *13* (22), 6037–6044. https://doi.org/10.1002/cssc.202002049.

(15) Ratcliff, E. L.; Meyer, J.; Steirer, K. X.; Garcia, A.; Berry, J. J.; Ginley, D. S.; Olson, D. C.; Kahn, A.; Armstrong, N. R. Evidence for Near-Surface NiOOH Species in Solution-Processed NiO_x Selective Interlayer Materials: Impact on Energetics and the Performance of Polymer Bulk Heterojunction Photovoltaics. *Chem. Mater.* **2011**, *23* (22), 4988–5000. https://doi.org/10.1021/cm202296p.

(16) Yamashita, T.; Hayes, P. Analysis of XPS Spectra of Fe^{2+} and Fe^{3+} Ions in Oxide Materials. *Appl. Surf. Sci.* **2008**, *254* (8), 2441–2449. https://doi.org/10.1016/j.apsusc.2007.09.063.

(17) Grosvenor, A. P.; Biesinger, M. C.; Smart, R. S. C.; McIntyre, N. S. New Interpretations of XPS Spectra of Nickel Metal and Oxides. *Surf. Sci.* **2006**, *600* (9), 1771–1779. https://doi.org/10.1016/j.susc.2006.01.041.

(18) Gallenberger, J.; Moreno Fernández, H.; Alkemper, A.; Li, M.; Tian, C.; Kaiser, B.; Hofmann, J. P. Stability and Decomposition Pathways of the NiOOH OER Active Phase of NiO_x Electrocatalysts at Open Circuit Potential Traced by Ex Situ and in Situ Spectroscopies. *Catal. Sci. Technol.* **2023**, *13* (16), 4693–4700. https://doi.org/10.1039/D3CY00674C.

(19) Jensen, D. S.; Kanyal, S. S.; Madaan, N.; Vail, M. A.; Dadson, A. E.; Engelhard, M. H.; Linford, M. R. Silicon (100)/SiO_2 by XPS. *Surf. Sci. Spectra* **2013**, *20* (1), 36–42. https://doi.org/10.1116/11.20121101.

(20) Weidler, N.; Schuch, J.; Knaus, F.; Stenner, P.; Hoch, S.; Maljusch, A.; Schäfer, R.; Kaiser, B.; Jaegermann, W. X-Ray Photoelectron Spectroscopic Investigation of Plasma-Enhanced Chemical Vapor Deposited NiO_x, $NiO_x(OH)_y$, and $CoNiO_x(OH)_y$: Influence of the Chemical Composition on the Catalytic Activity for the Ox. *J. Phys. Chem. C* **2017**, *121* (12), 6455–6463. https://doi.org/10.1021/acs.jpcc.6b12652.

(21) Zhao, Y.; Descamps, J.; Sojic, N.; Loget, G. All-Optical Electrochemiluminescence at Metal-Insulator-Semiconductor Diodes. *J. Phys. Chem. Lett.* **2024**, *15* (1), 148–155.

https://doi.org/10.1021/acs.jpclett.3c03220.

(22) Aroonratsameruang, P.; Pattanasattayavong, P.; Dorcet, V.; Mériadec, C.; Ababou-Girard, S.; Fryars, S.; Loget, G. Structure-Property Relationships in Redox-Derivatized Metal-Insulator-Semiconductor (MIS) Photoanodes. *J. Phys. Chem. C* **2020**, *124* (47), 25907–25916. https://doi.org/10.1021/acs.jpcc.0c08971.

(23) Sivula, K. Mott-Schottky Analysis of Photoelectrodes: Sanity Checks Are Needed. *ACS Energy Lett.* **2021**, *6* (7), 2549–2551. https://doi.org/10.1021/acsenergylett.1c01245.

(24) Hankin, A.; Bedoya-Lora, F. E.; Alexander, J. C.; Regoutz, A.; Kelsall, G. H. Flat Band Potential Determination: Avoiding the Pitfalls. *J. Mater. Chem. A* **2019**, *7* (45), 26162–26176. https://doi.org/10.1039/c9ta09569a.

(25) Yao, T.; Chen, R.; Li, J.; Han, J.; Qin, W.; Wang, H.; Shi, J.; Fan, F.; Li, C. Manipulating the Interfacial Energetics of N-Type Silicon Photoanode for Efficient Water Oxidation. *J. Am. Chem. Soc.* **2016**, *138* (41), 13664–13672. https://doi.org/10.1021/jacs.6b07188.

(26) Mattinen, M.; Schröder, J.; D'Acunto, G.; Ritala, M.; Jaramillo, T. F.; Stevens, M. B.; Bent, S. F. Dynamics of Precatalyst Conversion and Iron Incorporation in Nickel-Based Alkaline Oxygen Evolution Reaction Catalysts. *Cell Reports Phys. Sci.* **2024**, *5* (11), 102284. https://doi.org/10.1016/j.xcrp.2024.102284.

(27) Huang, Z.; Li, P.; Feng, M.; Zhu, W.; Woldu, A. R.; Tong, Q.-X.; Hu, L. Unlocking Fe(III) Ions Improving Oxygen Evolution Reaction Activity and Dynamic Stability of Anodized Nickel Foam. *Inorg. Chem.* **2024**, *63* (33), 15493–15502. https://doi.org/10.1021/acs.inorgchem.4c02646.

(28) Memming, R. *Semiconductor Electrochemistry*; Wiley, 2015. https://doi.org/10.1002/9783527688685.

(29) Lechaptois, L.; Prado, Y.; Pluchery, O. KPFM Visualisation of the Schottky Barrier at the Interface between Gold Nanoparticles and Silicon. *Nanoscale* **2023**, *15* (16), 7510–7516. https://doi.org/10.1039/d3nr00178d.

(30) Tung, R. T. Electron Transport of Inhomogeneous Schottky Barriers. *Appl. Phys. Lett.* **1991**, *58* (24), 2821–2823. https://doi.org/10.1063/1.104747.

(31) Tung, R. T. Electron Transport at Metal-Semiconductor Interfaces: General Theory. *Phys. Rev. B* **1992**, *45* (23), 13509–13523. https://doi.org/10.1103/PhysRevB.45.13509.

(32) Honsberg, C.; Bowden, S. *PVEducation*. https://www.pveducation.org/ (accessed 2025-10-13).

(33) Kresse, G.; Furthmüller, J. Efficient Iterative Schemes for Ab Initio Total-Energy Calculations Using a Plane-Wave Basis Set. *Phys. Rev. B* **1996**, *54* (16), 11169–11186. https://doi.org/10.1103/PhysRevB.54.11169.

(34) Blöchl, P. E. Projector Augmented-Wave Method. *Phys. Rev. B* **1994**, *50* (24), 17953–17979.

https://doi.org/10.1103/PhysRevB.50.17953.

(35) Perdew, J. P.; Burke, K.; Ernzerhof, M. Generalized Gradient Approximation Made Simple. *Phys. Rev. Lett.* **1996**, *77* (18), 3865–3868. https://doi.org/10.1103/PhysRevLett.77.3865.

(36) Steinmann, S. N.; Corminboeuf, C. Comprehensive Benchmarking of a Density-Dependent Dispersion Correction. *J. Chem. Theory Comput.* **2011**, *7* (11), 3567–3577. https://doi.org/10.1021/ct200602x.

(37) Treps, L.; Ermacora, T.; Giacomelli, A.; Michel, C.; Steinmann, S. N. Surface State of NiOOH under Oxidative Conditions: Can Dopants Induce Surface Oxidation? *Electrochim. Acta* **2025**, *524* (1), 145960. https://doi.org/10.1016/j.electacta.2025.145960.

(38) Dudarev, S.; Botton, G. Electron-Energy-Loss Spectra and the Structural Stability of Nickel Oxide: An LSDA+U Study. *Phys. Rev. B - Condens. Matter Mater. Phys.* **1998**, *57* (3), 1505–1509. https://doi.org/10.1103/PhysRevB.57.1505.

(39) Li, Y.; Liu, Z. Structure and Water Oxidation Activity of 3d Metal Oxides. *WIREs Comput. Mol. Sci.* **2016**, *6* (1), 47–64. https://doi.org/10.1002/wcms.1236.

(40) Liao, P.; Keith, J. A.; Carter, E. A. Water Oxidation on Pure and Doped Hematite (0001) Surfaces: Prediction of Co and Ni as Effective Dopants for Electrocatalysis. *J. Am. Chem. Soc.* **2012**, *134* (32), 13296–13309. https://doi.org/10.1021/ja301567f.

(41) Nørskov, J. K.; Rossmeisl, J.; Logadottir, A.; Lindqvist, L.; Kitchin, J. R.; Bligaard, T.; Jónsson, H. Origin of the Overpotential for Oxygen Reduction at a Fuel-Cell Cathode. *J. Phys. Chem. B* **2004**, *108* (46), 17886–17892. https://doi.org/10.1021/jp047349j.

(42) Mathew, K.; Kolluru, V. S. C.; Mula, S.; Steinmann, S. N.; Hennig, R. G. Implicit Self-Consistent Electrolyte Model in Plane-Wave Density-Functional Theory. *J. Chem. Phys.* **2019**, *151* (23), 234101. https://doi.org/10.1063/1.5132354.

(43) Wang, V.; Xu, N.; Liu, J.-C.; Tang, G.; Geng, W.-T. VASPKIT: A User-Friendly Interface Facilitating High-Throughput Computing and Analysis Using VASP Code. *Comput. Phys. Commun.* **2021**, *267*, 108033. https://doi.org/10.1016/j.cpc.2021.108033.

(44) Sundararaman, R.; Goddard, W. A.; Arias, T. A. Grand Canonical Electronic Density-Functional Theory: Algorithms and Applications to Electrochemistry. *J. Chem. Phys.* **2017**, *146* (11), 114104. https://doi.org/10.1063/1.4978411.

(45) Steinmann, S. N.; Sautet, P. Assessing a First-Principles Model of an Electrochemical Interface by Comparison with Experiment. *J. Phys. Chem. C* **2016**, *120* (10), 5619–5623. https://doi.org/10.1021/acs.jpcc.6b01938.

(46) Hörmann, N. G.; Andreussi, O.; Marzari, N. Grand Canonical Simulations of Electrochemical Interfaces in Implicit Solvation Models. *J. Chem. Phys.* **2019**, *150* (4), 041730. https://doi.org/10.1063/1.5054580.

(47) Melander, M. M.; Kuisma, M. J.; Christensen, T. E. K.; Honkala, K. Grand-Canonical Approach to Density Functional Theory of Electrocatalytic Systems: Thermodynamics of Solid-Liquid Interfaces at Constant Ion and Electrode Potentials. *J. Chem. Phys.* **2019**, *150* (4), 041706. https://doi.org/10.1063/1.5047829.

(48) Louie, M. W.; Bell, A. T. An Investigation of Thin-Film Ni-Fe Oxide Catalysts for the Electrochemical Evolution of Oxygen. *J. Am. Chem. Soc.* **2013**, *135* (33), 12329–12337. https://doi.org/10.1021/ja405351s.

(49) Magnier, L.; Cossard, G.; Martin, V.; Pascal, C.; Roche, V.; Sibert, E.; Shchedrina, I.; Bousquet, R.; Parry, V.; Chatenet, M. Fe–Ni-Based Alloys as Highly Active and Low-Cost Oxygen Evolution Reaction Catalyst in Alkaline Media. *Nat. Mater.* **2024**, *23* (2), 252–261. https://doi.org/10.1038/s41563-023-01744-5.

(50) Abidi, N.; Sahu, A.; Raybaud, P.; Steinmann, S. N. Electrochemical Potential-Dependent Stability and Activity of MoS3 during the Hydrogen Evolution Reaction. *ACS Catal.* **2023**, *13* (23), 15290–15300. https://doi.org/10.1021/acscatal.3c03292.

(51) Lee, S. A.; Lee, T. H.; Kim, C.; Choi, M. J.; Park, H.; Choi, S.; Lee, J.; Oh, J.; Kim, S. Y.; Jang, H. W. Amorphous Cobalt Oxide Nanowalls as Catalyst and Protection Layers on N-Type Silicon for Efficient Photoelectrochemical Water Oxidation. *ACS Catal.* **2020**, *10* (1), 420–429. https://doi.org/10.1021/acscatal.9b03899.

(52) Burke, M. S.; Kast, M. G.; Trotochaud, L.; Smith, A. M.; Boettcher, S. W. Cobalt-Iron (Oxy)Hydroxide Oxygen Evolution Electrocatalysts: The Role of Structure and Composition on Activity, Stability, and Mechanism. *J. Am. Chem. Soc.* **2015**, *137* (10), 3638–3648. https://doi.org/10.1021/jacs.5b00281.

(53) Pham, T. H. M.; Shen, T. H.; Ko, Y.; Zhong, L.; Lombardo, L.; Luo, W.; Horike, S.; Tileli, V.; Züttel, A. Elucidating the Mechanism of Fe Incorporation in In Situ Synthesized Co-Fe Oxygen-Evolving Nanocatalysts. *J. Am. Chem. Soc.* **2023**, *145* (43), 23691–23701. https://doi.org/10.1021/jacs.3c08099.

(54) Zhang, T.; Nellist, M. R.; Enman, L. J.; Xiang, J.; Boettcher, S. W. Modes of Fe Incorporation in Co–Fe (Oxy)Hydroxide Oxygen Evolution Electrocatalysts. *ChemSusChem* **2019**, *12* (9), 2015–2021. https://doi.org/10.1002/cssc.201801975.

(55) Mathur, A.; Sert, A.; Linic, S. Common Misconceptions in the Analysis of Critical Figures of Merit for Functioning Electrocatalyst/Semiconductor Photoelectrocatalysts under Solar Water-Splitting Conditions. *ACS Energy Lett.* **2024**, *9* (8), 4136–4146. https://doi.org/10.1021/acsenergylett.4c01365.

(56) King, A. J.; Weber, A. Z.; Bell, A. T. Understanding Photovoltage Enhancement in Metal-Insulator Semiconductor Photoelectrodes with Metal Nanoparticles. *ACS Appl. Mater. Interfaces* **2024**, *16* (28), 36380–36391. https://doi.org/10.1021/acsami.4c05928.
